# A Nonketotic Hyperglycinemia Mouse Shows Wide‐Ranging Biochemical Consequences of Elevated Glycine, Reduced Folate One‐Carbon Charging, and Serine Deficiency

**DOI:** 10.1002/jimd.70137

**Published:** 2026-01-28

**Authors:** Michael A. Swanson, Hua Jiang, Lakshmi Divya Kolora, Rachel Molino, Richard Reisdorph, Cole R. Michel, Katrina A. Doenges, Kit‐Yi Leung, Xiangping Lin, Frank Wong, Samual Lancaster, Basil Michael, Michael Snyder, Daniella H. Hock, David A. Stroud, Tim Wood, Robert Binard, Laura Anderson‐Lehman, Uwe Christians, Erland Arning, Marisa W. Friederich, Roxanne A. Van Hove, Kenneth N. MacLean, Nicholas D. E. Greene, Johan L. K. Van Hove

**Affiliations:** ^1^ Department of Pediatrics, Section of Clinical Genetics and Metabolism University of Colorado Anschutz Medical Campus Aurora Colorado USA; ^2^ Department of Pharmaceutical Sciences, Skaggs School of Pharmacy and Pharmaceutical Sciences University of Colorado Anschutz Medical Campus Aurora Colorado USA; ^3^ Developmental Biology and Cancer Department, UCL Great Ormond Street Institute of Child Health University College London London UK; ^4^ Department of Genetics, Stanford School of Medicine Stanford University Stanford California USA; ^5^ Center for Genomics and Personalized Medicine Stanford University Stanford California USA; ^6^ Department of Biochemistry and Pharmacology, Bio21 Molecular Science and Biotechnology Institute The University of Melbourne Parkville Victoria Australia; ^7^ Murdoch Children's Research Institute Melbourne Victoria Australia; ^8^ Victorian Clinical Genetics Services Murdoch Children's Research Institute Melbourne Victoria Australia; ^9^ Department of Pathology and Laboratory Medicine Children's Hospital Colorado Aurora Colorado USA; ^10^ Department of Anesthesiology, iC42 Clinical Research and Development University of Colorado Anschutz Medical Campus Aurora Colorado USA; ^11^ Institute of Metabolic Disease Baylor Scott and White Research Institute Dallas Texas USA

**Keywords:** D‐serine, glycine, lipidomics, L‐serine, metabolomics, methyl‐tetrahydrofolate, nonketotic hyperglycinemia, proteomics

## Abstract

Nonketotic hyperglycinemia is a severe neonatal epileptic encephalopathy caused by deficient glycine cleavage enzyme activity, for which currently no effective treatment exists. Incomplete understanding of brain biochemistry represents a major knowledge gap to develop new treatments. We examined the biochemistry in blood, liver, cortex, hippocampus, and cerebellum of a mouse model homozygous for the *Gldc* variant p.Ala394Val. Glycine was increased in all compartments and caused increased brain neurotoxic metabolites guanidinoacetate and methylglyoxal, and also N‐acetylglycine and cystathionine. The glycine extruding transporter Slc6a20 was increased. There was reduced one‐carbon folate charging with secondarily reduced methionine in the cortex, and reduced alternative one‐carbon donors L‐serine and formate. Serine deficiency was associated with reduced amounts of sphingosine, sphingomyelin, and ceramide species important for myelination, but not phosphatidylserines. There was a region‐specific deficiency of D‐serine in the cortex and hippocampus. This difference, also present in humans, was strain‐ and age‐related, most evident in young J129X1/SvJ mice, reflecting symptomatology. There was no evidence of oxidative stress or a bioenergetic defect. The biochemistry of the nonketotic hyperglycinemia mouse model can be traced to three components: increased glycine, reduced folate one‐carbon charging, and decreased L‐ and D‐serine. These changes will need to be addressed in new therapeutic approaches.

## 1 Introduction

The glycine cleavage enzyme system (GCS) consists of 4 protein subunits (P, T, H, and L) and breaks down glycine to carbon dioxide and ammonia with generation of 5,10‐methylene‐tetrahydrofolate (THF). It is primarily expressed in the liver and brain, where it is primarily present in astrocytes. Following the recognition of glycine as both a neurotransmitter and as a neuromodulator on the N‐methyl‐D‐aspartate (NMDA) receptor, the function of the GCS was considered to reduce glycine levels to enable this role. The recognition of the requirement for the GCS activity in neural tube closure through its provision of 5,10‐methylene‐THF suggested additional roles, which also appear important in cancer biology. The critical role of the GCS in brain function is emphasized by a severe disorder caused by its deficient activity, nonketotic hyperglycinemia (NKH).

Nonketotic hyperglycinemia is a rare genetic neurometabolic condition characterized by deficient glycine cleavage enzyme system (GCS) activity [1, 2]. Mutations in the *GLDC* gene encoding for the P‐protein (glycine decarboxylase) cause 80% of cases [1]. NKH is clinically classified as severe or attenuated based on the severity of symptomatology. The majority of patients (85%) have the severe phenotype, presenting with neonatal epileptic encephalopathy, and develop absent psychomotor development, spasticity, cortical blindness, and therapy‐resistant epilepsy. Patients with the attenuated form have some residual enzyme activity, as little as 1% [3]. They make a variable degree of developmental progress, have limited or no spasticity, and no cortical blindness, and have treatable or no epilepsy [1, 3, 4].

Current therapy is focused on mitigating the effect of excessive glycine and consists of glycine reduction strategies using either high‐dose benzoate or a ketogenic diet [5, 6, 7, 8]. Treatment aimed at mitigating the overstimulation of the NMDA‐type glutamatergic receptor, of which glycine is an allosteric activator, using either dextromethorphan or ketamine has limited, if any, impact [9]. Glycine reduction treatment results in increased wakefulness and less propensity for epilepsy, but only in attenuated NKH patients is early treatment associated with limited improvement in outcome [4, 10]. No new treatments have been developed since the introduction of dextromethorphan in 1992 [11]. New therapeutic approaches are being developed with gene therapy and new biochemical approaches under development [12]. Evaluation of the impact of these therapies requires well‐characterized animal models for understanding pathophysiology and testing treatment efficacy.

A major knowledge gap to develop new treatments is our incomplete understanding of the biochemistry of nonketotic hyperglycinemia. The elevation of brain glycine is well known [13]. A reduction of both L‐serine and D‐serine has been documented in brain and cerebrospinal fluid (CSF) [14, 15]. Excess glycine can conjugate with various acyl‐coenzyme A esters resulting in increased acylglycines as shown in GLDC‐deficient NKH mice and observed in urine organic acids analysis of NKH patients [16]. Further, an end product deficiency of one‐carbon charging of folate was documented in GLDC‐deficient brain and embryos, impacting the development of the neural tube and fetal brain [17, 18, 19]. Oxidative stress owing to excess glycine has been hypothesized to play a role in NKH based on studies in rats where injection of glycine in the brain caused elevated thiobarbituric acid‐reactive substances (TBARS) [20, 21].

We previously described the generation and phenotype of an NKH mouse model homozygous for the p.Ala394Val missense variant in *Gldc* on two strain backgrounds C57Bl/6J (B6) and 129X1/SvJ (J129) [22], referred to here for brevity as NKH mice. This variant is homologous to the recurring human mutation p.Ala389Val, which causes an attenuated form of NKH [3]. The model showed a reduction in GCS enzyme activity in vivo, and increased glycine levels in blood and brain. Fewer mutant mice were born than predicted, likely reflecting lethal neural tube defects, and mice on the B6 background developed postnatal hydrocephalus. Young mice showed impaired weight gain, particularly after weaning, with a nadir at 5 weeks postnatal age, and later recovery by 10 weeks. Epilepsy was not directly observed, but there was an increase in the spontaneous rate of electrographic spikes on electroencephalography, exacerbated by glycine challenge. Female J129 mice showed signs of encephalopathic behavior. Young J129 mice were primarily studied as the most closely reflecting human symptomatology, but for robustness in additional analyses were compared with adult mice and with the B6 mice.

In this study, we extensively characterized the biochemical changes in a mouse model of the neurologic disease NKH, with primary focus on the brain cortex as the most clinically affected area. We measured key metabolites in multiple brain regions (forebrain cortex, hippocampus, and cerebellum) together with metabolomics and proteomics studies in the brain cortex.

## 2 Methods

### 2.1 Mouse Models

Mouse studies were carried out with approval from the Institutional Animal Care and Use Committee of the University of Colorado Anschutz Medical Campus (IACUC# 00413 and 01520). Mice carrying the *Gldc* p.Ala394Val mutation were generated through homologous recombination in ES cells of a hybrid of C57Bl/6J (B6) and J129X1/SvJ mice. Selection cassettes were removed by Cre‐mediated recombination. The founder mouse was cross bred with marker assisted breeding to generate two congenic lines on the C57Bl/6J (B6) and J129X1/SvJ (J129) background strains as previously described [22]. Mice used in the experiments were from asymptomatic heterozygote intercrosses to avoid negative selection on the phenotype. Unless otherwise stated, young mice were analyzed at 5 weeks of age, and old mice were analyzed at 12.9 weeks of age. Food was removed 4 h before euthanasia to minimize the impact of nutrition on metabolite levels. After euthanasia, mouse tissues (plasma, liver, brain divided into cortex, hippocampus, and cerebellum) were harvested within minutes, snap‐frozen and stored at—80°C until analysis. Immediately before analysis, tissues were homogenized in 2:1 (μL/mg tissue) phosphate buffered saline (PBS) pH 7.4 using 0.5 mm zirconium oxide beads in a Storm Pro Bullet Blender tissue homogenizer (Next Advance, Troy, NY, USA).

### 2.2 Amino Acids

Stereoselective amino acids were measured after chiral derivatization with N‐isobutyryl‐L‐cysteine (IBC) and *o*‐phthaldialdehyde (OPA) by reverse phase high‐pressure liquid chromatography (HPLC) on an XBridge C18 column (3.5 μM, 4.6 × 150 mm, Waters, Milford, MA) with fluorescence detection [15, 23]. Total homocysteine, cysteine, cysteinylglycine, and total glutathione were measured after reduction with TCEP (Thermo Fisher, Waltham, MA) followed by derivatization with 4‐fluoro‐7‐sulfobenzofurazan (SBDF, Alfa Aesar, Averham, MA) using reverse phase HPLC with fluorescence detection using as internal standard N‐(2‐mercaptopropionyl)glycine (Sigma‐Aldrich, St. Louis, MA) [24]. Analysis of 42 amino acids, including γ‐aminobutyric acid (GABA), was measured in brain cortex homogenate using MassTrack amino acid analysis method (Waters, Milford, MA, USA) on a Waters Acuity UPLC system with UV detection [25].

### 2.3 Formate and Folate Metabolites

Formate was measured using stable isotope dilution gas chromatography‐tandem mass spectrometry after derivatization with benzyl alcohol in the presence of methyl chloroformate and pyridine using a previously described method with modifications as described in Supporting Information [26]. Intracellular folate species were measured in brain cortex by ultra‐high‐performance‐liquid chromatography (UPLC)‐tandem mass spectrometry using multiple reaction monitoring [17, 18, 27].

### 2.4 Other Quantified Metabolites

Creatine and guanidinoacetate were measured in homogenates of brain cortex and liver tissue by liquid chromatography–tandem mass spectrometry (LC–MS/MS) as previously described with minor modifications as described in Supporting Information [28, 29]. S‐adenosylmethionine (SAM) and S‐adenosylhomocysteine (SAH) were measured in tissue homogenates by tandem mass spectrometry as described for plasma [30]. Acylcarnitines were measured as their butyl‐esters by LC–MS/MS [31]. Tissue levels of NADPH and NADP^+^ were measured by fluorescence intensity using the NADP^+^/NADPH assay kit (Cell Biolabs, San Diego, CA, MET‐5031); TBARS was measured by fluorescence intensity using the Oxiselect TBARS Assay MDA quantification ELISA kit (STA‐330, Cell Biolabs); and methylglyoxal was measured using the Oxiselect Methylglyoxal Competitive ELISA kit (STA‐811, Cell Biolabs) using a 4PL standard curve analysis.

### 2.5 Metabolomic and Lipidomic Profiling

Metabolites were analyzed using Thermo Q‐Exactive Plus/HF mass spectrometers coupled to high‐performance liquid chromatography systems as further described in Supporting Information [32, 33]. Hydrophobic and hydrophilic metabolites were separated using a reverse phase and a HILIC column respectively [32]. Metabolites were identified based on MS/MS spectra as well as accurate mass and retention time, and quantified based upon ion intensity after normalization to internal standards. Lipidomics profiling was performed on the SCIEX Lipidyzer platform (Sciex, Framingham, MA) which consists of differential ion mobility spectrometry (DMS) combined with a Sciex QTRAP 6500 mass spectrometer, which delivers high levels of sensitivity and robustness for multiple reaction monitoring and allows accurate quantification of species belonging to 13 lipid classes. DMS separates lipids based on their dipole moment, which overcomes the isobaric species overlap. Quantification was done with a mixture of 58 isotopically labeled standards [33]. Individual data were analyzed on Perseus software (https://maxquant.net/perseus), similar to proteomics below. Lipids in the same category were analyzed as a group by comparing the averages of the WT and MUT series using paired testing.

For analysis of phosphatidylserines, samples were extracted with a modified methyl tert‐butyl ether (MTBE) liquid–liquid extraction method, separated by liquid chromatography on a Zorbax SB‐C18 Rapid Resolution High‐Definition column and analyzed by electrospray high‐resolution mass spectrometry on an Agilent 6545 quadrupole time‐of‐flight mass spectrometer (QTOF) as previously described and further detailed in the Supplemental Methods [34].

### 2.6 Proteomics Analysis

Proteomic analysis was performed in mouse brain cortex tissue homogenates as previously described [35]. In brief, proteins were solubilized with sodium dodecyl sulfate and loaded on an S‐trap column (ProtiFi, Newport, NY), disulfide bonds were reduced with TCEP and stabilized using chloroacetamide before overnight digestion with trypsin. After washing, the peptides were eluted and analyzed by reverse phase liquid chromatography and label‐free data‐independent acquisition (DIA) on an OrbiTrap Eclipse Mass Spectrometer (Thermo Fisher Scientific, San Jose, CA) using conditions as previously described [35]. Raw data files were processed on Spectronaut 18.7 software (Biognosys AG, Schlieren, Swtizerland) [36, 37], excluding single hit proteins, and identified on the UniProt mouse database (canonical peptides + isoforms, reviewed, 25 346 entries) for label‐free quantification. The total number of proteins was reviewed for QC, and proteins were then analyzed on Perseus software v.2.0.11 (Max Planck Institute for Biochemistry) [38]. Log2‐transformed label‐free quantification intensities were grouped in WT and MUT groups, two‐sample *t*‐tests run between groups, and Volcano plots were generated using the built‐in scatter plot function with *P*‐value set to 0.05 equivalent (−log_10_ = 1.301).

Individual proteins were quantified by sodium dodecyl‐polyacrylamide gel electrophoresis (SDS‐PAGE) followed by western blot with details described in Supporting Information. To assess Nrf2 activation via nuclear translocation, cytoplasmic and nuclear protein fractions were prepared using the NE‐PER Nuclear and Cytoplasmic Extraction Kit (Thermo Fisher Scientific, Cat# 78833). Nrf2 levels in the nuclear and cytosolic fractions were quantified using the Mouse NRF2 ELISA Kit (Novus Biologicals, Centennial, CO, Cat# NBP2‐76758).

### 2.7 Mitochondrial Enzyme Activities

Respiratory chain enzyme activities were measured in whole brain homogenates in post 600 g supernatant spectrophotometrically at 30°C as previously described [39].

### 2.8 Statistical Analysis

Normality of distribution was first evaluated using Shapiro–Wilk and Kolmogorov–Smirnov statistics. Data were presented as mean and standard deviation if normally distributed, and as median and interquartiles if not normally distributed. Comparisons between two populations were done by Student *t*‐test or by Mann–Whitney‐U statistic if normally distributed or not, respectively. Comparisons between more than two populations were done by one‐way ANOVA or by Kruskal‐Wallis test if normally distributed or not, respectively. Paired testing was done by paired Student's *t*‐test or by paired Wilcoxon test. Correction for multiple testing was done by the Benjamini‐Hochberg procedure. Relationships between two variables were calculated as the Pearson correlation coefficient for normally distributed populations or the Spearman rank correlation for non‐normally distributed populations. Ingenuity pathway analysis (Qiagen, Redwood City, CA, USA) was performed for the proteomics data, for proteins with a *p*‐value cutoff of 0.05 using standard settings [40]. For analysis of the lipidomics data, data were filtered for low variance (RSD > 10%) and low abundance (median intensity > 20%), before normalization by the median, log2 transformation and Pareto scaling. After principal component analysis was done using MetaboAnalyst [41], Biopan analysis was used for lipid networks and fatty acid networks analysis [42], and lipid ontology was generated using LION [43] (see Supplemental Methods).

## 3 Results

### 3.1 Core Metabolites

We first analyzed core metabolites directly related to glycine metabolism. Comparing homozygous p.Ala394Val mutant (MUT) mice with wild type (WT) on a J129 strain background (Table 1), in plasma glycine concentration was doubled, and methionine was borderline decreased by 20%, but homocysteine and cysteine were unchanged. In liver tissue, glycine was increased by 144%, with minor decreases in homocysteine and cysteine. No changes were noted in L‐serine in plasma or liver.

**TABLE 1:** Comparison of core metabolites in 5‐week‐old J129 mice comparing wild type versus mutant mice.

J129 5 weeks old	WT mice *N* = 16		MUT mice *N* = 14		Comparison		Change
Analyte	Mean ± SD	Median (IQR)	Mean ± SD	Median (IQR)	*p*‐value *t*‐test or MWU	Benjamini‐ Hochberg *p*‐value	% change
Plasma analytes in μmol/L							
Glycine	333 ± 34*	324 (311–364)	695 ± 175	722 (533–805)	< 0.001*	**< 0.01**	**+109%**
L‐serine	139 ± 30	132 (117–168)	129 ± 37	123 (94–171)	0.397	0.5525	−7%
Methionine	41.1 ± 9.4*	40.6 (36.0–43.6)	32.7 ± 7.7	30.7 (26.0–41.5)	0.012*	**0.04**	**−20%**
Homocysteine†	4.92 ± 1.11	4.99 (4.15–5.98)	4.76 ± 1.75	4.39 (3.63–6.12)	0.769	0.769	−3%
Cysteine†	128 ± 21	128 (117–135)	133 ± 42	123 (106–165)	0.735	0.769	+4%
Cysteinylglycine†	4.09 ± 0.34	4.08 (3.85–4.29)	3.13 ± 0.91	3.47 (2.26–3.83)	**0.002**	**0.01**	**−23%**
Threonine	115 ± 24	109 (96–129)	101 ± 30*	88 (79–119)	0.110*	0.275	−12%
Glutamate	48.5 ± 12.3	45.6 (40.7–55.4)	45.2 ± 10.2	45.3 (35.7–50.5)	0.525	0.5525	−7%
Glutamine	613 ± 78	609 (549–677)	578 ± 83	557 (502–653)	0.240	0.42	−6%
Glutathione†	74 ± 11*	80 (64–84)	68 ± 14	70 (60–78)	0.137*	0.38	−8%
Cortex analytes in nmol/g			nmol/g				
Glycine	855 ± 58	847 (801–915)	1581 ± 175	1583 (1458–1733)	**< 0.001**	**< 0.00325**	**+85%**
L‐serine	657 ± 52	660 (608–705)	565 ± 64	575 (546–598)	**< 0.001**	**< 0.00325**	**−14%**
D‐serine	302 ± 25	299 (290–313)	238 ± 32*	244 (226–258)	< 0.001*	**< 0.00325**	**−21%**
Methionine	70.8 ± 14.4*	65.5 (60.4–81.5)	53.5 ± 15.5	53.2 (41.5–64.0)	0.004*	**0.0104**	**−24%**
Homocysteine‡	3.80 ± 0.62*	3.80 (3.60–4.20)	3.54 ± 0.44	3.65 (3.20–3.80)	0.116*	0.215	−7%
Cysteine‡	111 ± 35	107 (87–125)	123 ± 13	123 (117–136)	0.253	0.215	+11%
Cysteinylglycine‡	2.81 ± 0.70	3.00 (2.15–3.25)	3.69 ± 0.80	3.45 (2.98–4.30)	**0.006**	**0.013**	**+31%**
Threonine	439 ± 62	437 (408–462)	410 ± 43	417 (376–443)	0.165	0.268	−7%
Glutamate #	16.82 ± 2.39*	17.17 (16.50–18.16)	16.37 ± 1.71	16.67 (15.55–17.66)	0.423*	0.470	−3%
Glutamine #	4.47 ± 1.11*	4.40 (4.12–4.61)	4.53 ± 1.43*	4.04 (3.81–4.87)	0.423*	0.470	+1%
Glutathione‡	558 ± 127	496 (469–668)	544 ± 67*	519 (484–586)	0.793*	0.793	−3%
Guanidinoacetate†	2.78 ± 0.41	2.75 (2.47–3.17)	3.72 ± 0.64	3.67 (3.31–3.99)	**< 0.001**	**< 0.00325**	**+34%**
Creatine†	2499 ± 260	2550 (2325–2691)	2600 ± 401	2630 (2309–2840)	0.434	0.470	+4%
Hippocampus analytes in nmol/g							
Glycine	928 ± 65	913 (877–1002)	2063 ± 162	2057 (1899–2180)	**< 0.001**	**0.0055**	**+122%**
L‐serine	719 ± 44	730 (696–749)	669 ± 40	668 (633–699)	**0.003**	**0.011**	**−7%**
D‐serine	274 ± 23*	274 (267–288)	232 ± 20	227 (211–248)	< 0.001*	**0.0055**	**−15%**
Methionine	83.4 ± 10.5	84.3 (77.2–91.1)	81.3 ± 20.3	80.5 (62.6–101.3)	0.741	0.982	−3%
Homocysteine	3.11 ± 0.56	3.09 (2.61–3.63)	3.08 ± 0.67	3.23 (2.33–3.71)	0.905	0.982	−1%
Cysteine	111 ± 28*	95.1 (89.5–146.1)	98 ± 27	92.8 (78.9–119.4)	0.240*	0.66	−12%
Cysteinylglycine	4.38 ± 1.28	5.13 (2.96–5.57)	4.52 ± 1.33	4.01 (3.30–5.96)	0.770	0.982	+3%
Threonine	334 ± 76	315 (279–364)	338 ± 47	350 (297–371)	0.864	0.982	+1%
Glutamate #	10.13 ± 0.27	10.05 (9.96–10.36)	10.13 ± 0.79	10.29 (9.41–10.81)	0.982	0.982	0%
Glutamine #	4.62 ± 0.48*	4.54 (4.36–4.68)	4.86 ± 1.57*	4.32 (3.99–5.07)	0.473*	0.982	+5%
Glutathione	493 ± 89	529 (406–576)	498 ± 90	492 (403–591)	0.890	0.982	+1%
Cerebellum analytes in nmol/g							
Glycine	917 ± 86	904 (871–965)	3217 ± 301	3212 (3040–3370)	**< 0.001**	**0.0033**	**+251%**
L‐serine	548 ± 79	515 (489–625)	621 ± 71	632 (555–672)	**0.013**	**0.0325**	**+13%**
Methionine	85.2 ± 5.6	84.5 (80.2–89.4)	79.5 ± 15.5	75.6 (70.1–86.6)	0.209	0.393	−7%
Homocysteine	2.53 ± 0.26	2.55 (2.46–2.71)	2.46 ± 0.18*	2.48 (2.36–2.58)	0.275*	0.393	−3%
Cysteine	83.8 ± 22.8	90.2 (81.9–94.8)	85.7 ± 15.3	81.5 (70.9–99.3)	0.794	0.794	+1%
Cysteinylglycine	6.99 ± 0.35*	6.98 (6.72–7.10)	7.26 ± 0.49	7.17 (6.82–7.67)	0.240*	0.393	+4%
Threonine	381 ± 60	396 (350–421)	519 ± 111	526 (444–587)	**< 0.001**	**0.0033**	**+36%**
Glutamate #	9.97 ± 0.48	10.01 (9.60–10.36)	10.10 ± 0.42*	10.26 (9.78–10.45)	0.498*	0.622	+1%
Glutamine #	6.51 ± 0.71*	6.51 (6.08–6.74)	6.97 ± 1.62*	6.50 (6.08–7.16)	0.759*	0.794	+7%
Glutathione	697 ± 29*	692 (674–726)	636 ± 40	642 (617–688)	< 0.001*	**0.0033**	**−9%**
Liver analytes in nmol/g							
Glycine	1851 ± 236	1851 (1717–2028)	4519 ± 771	4546 (3719–5217)	**< 0.001**	**0.012**	**+144%**
L‐serine	397 ± 66	400 (349–448)	386 ± 50	371 (351–428)	0.313	0.417	−3%
Methionine	117.8 ± 15.9	114.9 (107.0–129.1)	128.1 ± 14.7	124.5 (114.4–141.0)	**0.043**	0.1032	+9%
Homocysteine	6.04 ± 0.8	5.71 (5.44–6.90)	5.13 ± 0.79	5.25 (4.33–5.60)	**0.008**	**0.032**	**−15%**
Cysteine	525 ± 44	524 (485–565)	470 ± 40	469 (430–513)	**0.002**	**0.012**	**−10%**
Cysteinylglycine	14.60 ± 1.56	14.53 (13.29–15.78)	14.30 ± 2.10	14.72 (12.81–15.60)	0.667	0.667	−10%
Threonine	231 ± 60	206 (148–246)	195 ± 56	206 (148–246)	0.117	0.200	−16%
Glutamate #	1.24 ± 0.41*	1.12 (0.99–1.38)	1.25 ± 0.36	1.33 (0.88–1.47)	0.603*	0.658	+1%
Glutamine #	5.96 ± 1.07	5.81 (5.41–6.35)	5.40 ± 0.86	5.45 (4.84–6.15)	0.134	0.201	−9%
Glutathione	2125 ± 610	2001 (1647–2674)	2335 ± 566	2242 (1913–2847)	0.355	0.426	+10%
Guanidinoacetate	8.08 ± 2.49	7.34 (6.03–10.40)	10.59 ± 3.38	10.34 (8.15–13.46)	**0.034**	0.102	**+31%**
Creatine	87.73 ± 14.54	86.83 (77.23–94.38)	114.73 ± 61.80*	96.85 (85.40–121.3)	0.054*	0.108	**+31%**

*Note:* Values of metabolites in J129 mice at age 5 weeks are shown comparing wild type (WT) animals with homozygous mutant (MUT) animals. The distribution is shown as mean (AVG) and standard deviation (SD). Values of metabolites in plasma are in μM, and values of the metabolites in forebrain cortex, hippocampus and cerebellum are shown in nmol/g tissue (# except for glutamate and glutamine shown in μmol/g tissue). There were 16 WT and 14 MUT animals except as indicated † *N* = 14 and ‡ *N* = 13 for WT animals, and for liver there were 14 MUT and 14 WT animals. A significant deviation of the normal distribution is indicated by an asterisk*. Comparisons are done by Student *t*‐test or by Mann–Whitney‐U test if any of the two populations deviates from the normal distribution as indicated by an asterisk*, and *p*‐values shown. A Benjamini‐Hochberg corrected *p*‐value is provided for the multiple comparisons within each tissue type. Significant *p*‐values are highlighted in bold. The percentage differences between the MUT in comparison with the WT is given with green values for significant increase and red values for a significant decrease.

In brain cortex, glycine abundance was elevated by 85% in MUT mice compared with WT mice. Similar to humans [14, 15], there was a decrease of L‐serine (−14%) and even more of D‐serine (−21%). There was a positive correlation between glycine and L‐serine (*ρ* = 0.820, *p* < 0.001) and a moderate correlation of L‐serine and D‐serine (*ρ* = 0.556, *p* = 0.039). In one‐carbon metabolism, methionine was decreased (−24%), whereas homocysteine was unchanged.

In the hippocampus, we observed a similar increase in glycine (+122%), and a lesser decrease in L‐serine (−7%) and D‐serine (−15%), but no decrease in methionine. Here, the correlation between glycine and L‐serine (*ρ* = 0.912, *p* < 0.001) and between L‐serine and D‐serine (*ρ* = 0.855, *p* < 0.001) was strong. In the cerebellum, where serine racemase is not expressed, there was a larger increase in glycine (+251%), and, interestingly, here L‐serine was mildly increased (+13%) and positively correlated to glycine (*ρ* = 0.859, *p* < 0.001). This was accompanied by an increase in threonine by 36%, perhaps reflecting the 2‐aminoacetate pathway. In this region, methionine and homocysteine were both unchanged.

In MUT mice, increases in plasma glycine correlated with cerebellar glycine levels (*r* = 0.846, *p* ≤ 0.001) and mildly with liver glycine levels (*r* = 0.632, *p* = 0.015, Figure S1), but not with cortex or hippocampal glycine levels. No other core metabolites showed a correlation between plasma levels and liver levels.

We next evaluated sex‐ and age‐related effects. In young mice of the J129 strain, there were few within‐genotype sex differences (Table S1). In WT females compared with males, plasma L‐serine (+37%) and homocysteine (+37%) were increased. In the cortex, female WT mice had increased threonine and glutamate and decreased glutathione but unchanged glycine, serine, and methionine levels compared with males. In MUT mice, female mice have increased plasma glycine (+15%), L‐serine (+48%), and glutamine, whereas in the cortex and hippocampus, the glycine, serine, and methionine levels were unchanged, and there was only a decrease in glutathione (−19%) in female mice. In the cerebellum, though, females had higher glycine (+12%) and serine (+18%) levels with a decrease in methionine levels (−23%), with similar changes in plasma, but not in liver.

In adult mice at age 12.9 weeks comparing MUT to WT mice (Table 2), all three brain regions showed similar elevation of glycine. However, in one‐carbon metabolism the decrease in methionine was no longer significant, and L‐serine, which donates methyl‐groups via serine hydroxymethyltransferase, was less decreased and no longer significant, and the decrease of D‐serine (−12%) was less pronounced, still significant in cortex but no longer in hippocampus. In contrast, in plasma, in addition to elevated glycine (+303%), at this age, there was a moderate elevation of homocysteine (+59%).

**TABLE 2:** Comparison of core metabolites in mature 12.9‐week‐old J129 mice comparing wild type versus mutant mice.

Metabolite	WT mice		MUT mice		Comparison		Change
	Mean ± SD	Median (IQR)	Mean ± SD	Median (IQR)	*p*‐value student/MWU	*p*‐value Benjamini Hochberg	% change
**J129 12.9 weeks old**	***N* = 14**		***N* = 12**				
Plasma analytes in μmol/L							
Glycine	335 ± 38	337 (303–361)	1016 ± 88	1007 (948–1094)	**< 0.001**	**< 0.010**	**+303%**
L‐serine	155 ± 23*	147 (140–169)	163 ± 40*	149 (140–174)	0.781*	0.974	+5%
Methionine	55.7 ± 12.6	54.2 (45.3–63.7)	67.7 ± 18.4*	57.6 (55.0–83.9)	0.131*	0.352	+25%
Homocysteine	6.1 ± 2.0	5.98 (4.43–7.62)	9.7 ± 3.5	9.57 (7.43–12.33)	**0.002**	**0.010**	**+59%**
Cysteine	223 ± 53	215 (181–273)	224 ± 46	224 (180–273)	0.974	0.974	0%
Cysteinylglycine	1.9 ± 0.2	1.92 (1.70–2.08)	1.8 ± 0.3	1.84 (1.62–1.99)	0.466	0.877	**−**5%
Threonine	167 ± 22	166 (156–190)	186 ± 40	178 (156–206)	0.141	0.352	+11%
Glutamate	40.4 ± 7.9*	39.0 (33.3–46.8)	41.1 ± 11.0	37.4 (36.7–45.7)	0.899*	0.974	+2%
Glutamine	553 ± 71	541 (523–587)	535 ± 50	523 (495–565)	0.526	0.877	−3%
Glutathione	58 ± 21*	54.5 (50.7–73.0)	55 ± 19	58.6 (46.8–68.0)	0.860*	0.974	−5%
Cortex analytes in nmol/g							
Glycine	1175 ± 297*	1103 (1004–1186)	1703 ± 239	1708 (1450–1891)	< 0.001*	**< 0.011**	**+45%**
L‐serine	652 ± 98*	628 (582–680)	595 ± 66	586 (549–631)	0.118*	0.216	−9%
D‐serine	283 ± 24	283 (266–292)	249 ± 28	242 (226–266)	**0.003**	**0.0165**	**−12%**
Methionine	132 ± 37	127 (98–168)	127 ± 57*	124 (73–181)	0.560*	0.560	−4%
Homocysteine	3.23 ± 0.49	3.32 (2.80–3.70)	3.04 ± 0.52	3.06 (2.64–3.50)	0.347	0.424	−6%
Cysteine	126 ± 13	124 (120–133)	112 ± 24	112 (94–131)	0.084	0.216	−11%
Cysteinylglycine	2.94 ± 0.28	2.95 (2.67–3.20)	2.82 ± 0.44	2.76 (2.48–3.21)	0.425	0.467	−4%
Glutamate #	14.25 ± 0.88	14.28 (13.78–14.68)	13.82 ± 1.06	13.89 (13.24–14.24)	0.267	0.367	−3%
Glutamine #	3.94 ± 0.43	3.98 (3.60–4.25)	2.18 ± 0.18*	3.44 (3.27–3.61)	0.017*	0.0623	−45%
Glutathione	430 ± 30	426 (408–442)	408 ± 38	413 (384–440)	0.110	0.216	−5%
Hippocampus analytes in nmol/g							
Glycine	849 ± 72	827 (801–924)	1827 ± 187	1803 (1771–1985)	**< 0.001**	**0.0055**	**+115%**
L‐serine	675 ± 35	669 (652–706)	655 ± 40	649 (630–687)	0.178	0.245	−3%
D‐serine	256 ± 14	256 (247–263)	227 ± 12	228 (219–236)	**< 0.001**	**0.0055**	−11%
Methionine	62.0 ± 8.0	62.2 (56.2–67.4)	63.1 ± 6.0	61.9 (58.4–68.8)	0.687	0.811	+2%
Threonine	300 ± 37*	287 (277–321)	305 ± 16	310 (293–314)	0.160*	0.245	+2%
Homocysteine	3.02 ± 0.31	3.02 (2.76–3.30)	2.99 ± 0.16	2.98 (2.83–3.12)	0.737	0.811	−1%
Cysteine	317 ± 27	314 (302–341)	321 ± 49	317 (275–365)	0.845	0.845	+1%
Cysteinylglycine	5.20 ± 1.11*	5.00 (4.60–5.31)	5.04 ± 1.27*	4.74 (4.44–5.01)	0.176*	0.245	−3%
Glutamate #	12.02 ± 0.46	11.99 (11.80–12.36)	11.51 ± 0.48	11.45 (11.20–11.82)	**0.012**	**0.033**	**−4%**
Glutamine #	3.47 ± 0.32	3.47 (3.17–3.65)	3.18 ± 0.17	3.19 (3.05–3.34)	**0.010**	**0.033**	**−8%**
Glutathione	608 ± 37	613 (581–636)	585 ± 13	587 (579–594)	**0.042**	0.0924	−4%
Cerebellum analytes in nmol/g							
Glycine	849 ± 99	844 (780–899)	2789 ± 923*	2547 (2373.‐2789)	< 0.001*	**< 0.005**	**+228%**
L‐serine	534 ± 56	521 (499–583)	603 ± 69	603 (570–656)	**0.009**	**0.030**	**+13%**
Methionine	80.9 ± 5.1	79.6 (76.9–84.3)	78.6 ± 8.0	80.1 (73.9–84.4)	0.377	0.377	−3%
Threonine	452 ± 49*	444 (419–485)	609 ± 98	628 (577–673)	< 0.001*	**< 0.005**	**+35%**
Homocysteine	1.66 ± 0.28	1.59 (1.54–1.77)	1.91 ± 0.22	1.96 (1.71–2.06)	**0.021**	0.0525	+15%
Cysteine	333 ± 46	325 (296–353)	351 ± 46	352 (308–391)	0.357	0.377	+5%
Cysteinylglycine	4.93 ± 0.57	4.88 (4.42–5.19)	5.30 ± 0.69	5.55 (4.86–5.72)	0.147	0.245	+8%
Glutamate #	12.63 ± 0.37	12.73 (12.34–12.88)	12.26 ± 1.13	12.29 (11.33–13.27)	0.287	0.377	−3%
Glutamine #	5.17 ± 0.53	5.24 (4.71–5.48)	4.69 ± 0.54	4.66 (4.40–5.01)	**0.031**	0.062	−9%
Glutathione	574 ± 75	559 (528–617)	546 ± 71	533 (505–587)	0.344	0.377	−5%

*Note:* Values of metabolites are compared in mature 12.9‐week‐old J129 mice between wild type (WT) and mutant (MUT) mice with nonketotic hyperglycinemia. Values of metabolites in plasma are in μM, and values of the metabolites in forebrain cortex, hippocampus and cerebellum are shown in nmol/g tissue (# except for glutamate and glutamine shown in μmol/g tissue). The distribution in each category is provided with mean (AVG) and standard deviation (SD) given, and as the median and interquartile range (IQR). Comparison is done either as by the Student *t*‐test or by the Mann–Whitney‐U test (MWU) if not normally distributed as indicated by an asterisk*. A Benjamini‐Hochberg corrected *p*‐value is provided for the multiple comparisons within each tissue type. Significant *p*‐values are highlighted in bold. The percentage differences between the MUT in comparison with the WT is given with green values for significant increase and red values for a significant decrease.

To better understand these age‐related differences, when comparing the young 5‐week‐old mice with mature 12.9‐week‐old mice (Table S2), in WT and MUT mice, plasma methionine levels increased, and in MUT mice, plasma homocysteine levels increased. In MUT mice, plasma glycine (+46%), L‐serine, and threonine were increased. In the cortex, in WT animals, the glycine (+37%) and methionine levels (+87%) were increased, with a decrease in homocysteine (−15%), but not in the hippocampus or cerebellum. In MUT mice, in the cortex, glycine, serine, and threonine levels were unchanged, with glycine slightly decreased in the hippocampus and cerebellum. However, in one‐carbon metabolism, cortex methionine was increased markedly (+138%) with a mild decrease in cortex homocysteine (−14%), but not in the hippocampus or cerebellum. In hippocampus and cerebellum, glutamine decreased and glutamate increased in both WT and MUT animals, whereas in cortex, both glutamate and glutamine decreased.

### 3.2 Expanded Metabolites

Methionine is generated by transfer of a methyl group from 5‐methyltetrahydrofolate to homocysteine. The glycine cleavage system mediates a major supply of glycine‐derived one‐carbon units to folate metabolism, and we therefore analyzed the folate profile in the cortex of the 5‐week‐old mice on the B6 strain (Table 3). The distribution of the folate profile showed a significant decrease in the proportion of 5‐methyl‐tetrahydrofolate and a corresponding increase in unmethylated tetrahydrofolate (Table 3). The ratio of 5‐methyl‐tetrahydrofolate to tetrahydrofolate was significantly decreased (Table S3). This indicates a decrease in the end product of one‐carbon charging of folates. Mitochondrial folate metabolism generates formate and can be transferred to the cytosol. Formate can also be an important alternative source to charge folates by generating 10‐formyltetrahydrofolate [44, 45]. In the J129 strain, formate plasma levels were decreased in MUT 22.8 μM (18.5–32.0 μM) (median (IQR)) compared with WT mice (33.5 μM (29.4–43.5 μM), *p* = 0.038). One‐carbon units are passed from the folate cycle to the methionine cycle, but the methionine cycle components SAM and SAH were not changed in abundance (SAM: WT 20.3 ± 0.67 nmol/g tissue MUT 20.4 ± 1.12 nmol/g tissue, *p* = 0.8; SAH: WT 1.64 ± 0.17 nmol/g tissue, MUT 1.68 ± 0.18 nmol/g tissue, *p* = 0.69). Moreover, 5‐methyl‐thioadenosine was not changed in the polyamine synthesis related methionine salvage pathway, which also generates formate [46].

**TABLE 3:** Folate vitamers in mutant mice compared with wild‐type mice.

Metabolite	WT mice		MUT mice		Comparison	
Folate vitamer	Mean ± SD in %	median (IQR) in %	Mean ± SD in %	median (IQR) in %	*p*‐value student/MWU	*p*‐value Benjamini Hochberg
B6 5 weeks old	***N* = 8**		***N* = 8**			
Dihydrofolate	1.77 ± 1.84*	0.89 (0.72–2.39)	0.94 ± 0.45	0.82 v(0.66–1.29)	0.442*	0.442
Tetrahydrofolate	35.96 ± 6.80*	37.11 (34.85–40.87)	42.61 ± 4.45	42.29 (38.87–44.89)	0.015*	**0.045**
5‐methyl‐THF	59.39 ± 6.47	57.81 (54.69–61.12)	52.18 ± 5.17	52.07 (49.18–56.70)	**0.027**	**0.054**
5,10‐methylene‐THF	1.58 ± 0.35	1.58 (1.30–1.87)	1.78 ± 0.49	1.82 (1.38–2.19)	0.343	0.4116
5,10‐methenyl‐THF	0.18 ± 0.11	0.22 (0.08–0.27)	0.42 ± 0.23	0.31 (0.25–0.65)	**0.015**	**0.045**
10‐formyl‐THF	1.77 ± 0.44	1.74 (1.41–2.21)	2.07 ± 0.69	2.13 (1.52–2.60)	0.318	0.4116

*Note:* The distribution of folate vitamer species as a percentage of the total folates is provided in the brain cortex of 5‐week‐old mice in the B6 strain. The distribution in each category is provided with mean (AVG) and standard deviation (SD) given, and as the median and interquartile range (IQR). Comparison is done either as by the Student *t*‐test or by the Mann–Whitney‐U test (MWU) if not normally distributed as indicated by an asterisk*. A Benjamini‐Hochberg corrected *p*‐value is provided for the multiple comparisons within each tissue type. Significant *p*‐values are highlighted in bold.

Glycine conjugates with arginine to generate guanidinoacetate to synthesize creatine. Guanidinoacetate was increased by 34% in cortex, whereas creatine levels were unchanged (Table 1), as previously reported on a GLDC‐deficiency mice on a B6 strain [16]. It was similarly increased in liver (+31%) with borderline significance, with a sex difference in a univariate analysis of variance whereby mutation was significant (*p* = 0.029) and sex borderline (*p* = 0.064) (male higher than female). Within the mutant mice, there was only a weak trend to correlate cortex guanidinoacetate with plasma glycine (*r* = 0.470, *p* = 0.09), but considering MUT and WT mice together, cortex guanidinoacetate correlated mildly with cortex glycine levels *r* = 0.660, *p* < 0.001, and plasma glycine *r* = 0.718 *p* < 0.001 (Figure S2). Expanded amino acid analysis in the cortex of J129 mice (Table S4) showed only an increase in glycine levels as expected. The decrease in the amount of free GABA (−9%) was not significant, only ß‐alanine (a structural analog of glycine) was mildly decreased (−15%).

Glycine acetyl‐CoA transferase, which is strongly expressed in the entire brain [47], can synthesize 2‐aminoacetoacetate (a.k.a. 2‐amino‐3‐ketobutyrate), which in mice can be a substrate for L‐threonine dehydrogenase, which is also expressed in the brain [48] primarily in cerebellum [49]. In the cerebellum, threonine is increased, likely reflecting this pathway. This enzyme is a non‐functional pseudogene in humans [50]. The intermediate 2‐amino‐3‐ketobutyrate non‐enzymatically forms 2‐aminoacetate, which forms methylglyoxalate [51], which is toxic and forms adducts [52], and is detoxified with glutathione by glyoxalase 1 (Glo1) to lactoyl‐glutathione. The levels of Tdh were unchanged on western blots (Figure S3), and the levels of Glo1 and Gcat were unchanged on proteomics. Methylglyoxal adducts were markedly 7‐fold increase in cortex in MUT animals 129.3 (115.9–150.6) compared with WT 18.3 (7.7–29.3) MGO‐BSA/mg tissue, *p* < 0.001, but were unchanged between MUT and WT in liver, hippocampus, or cerebellum (Table S5A). Methylglyoxal can cause oxidative stress, which has been hypothesized in NKH based on studies in glycine‐treated rats where TBARS were elevated [20, 21]. There was no difference between WT and MUT animals in TBARS in cortex (9.04 ± 1.14 vs. 9.50 ± 1.07, *p* = 0.411) or liver (62.3 ± 5.6 vs. 64.1 ± 13.9, *p* = 0.734). Only in glycine‐treated animals was there a small difference in cortex (MUT + GLY 10.22 ± 0.73) (Figure S4). 2‐Aminoacetone could not be detected in either the liver or cortex with a lower limit of detection < 0.8 μM/mg tissue.

### 3.3 Metabolomics

To expand the range of metabolites evaluated, we performed metabolomics analysis covering both water‐soluble metabolomics and lipidomics.

**FIGURE 1:**
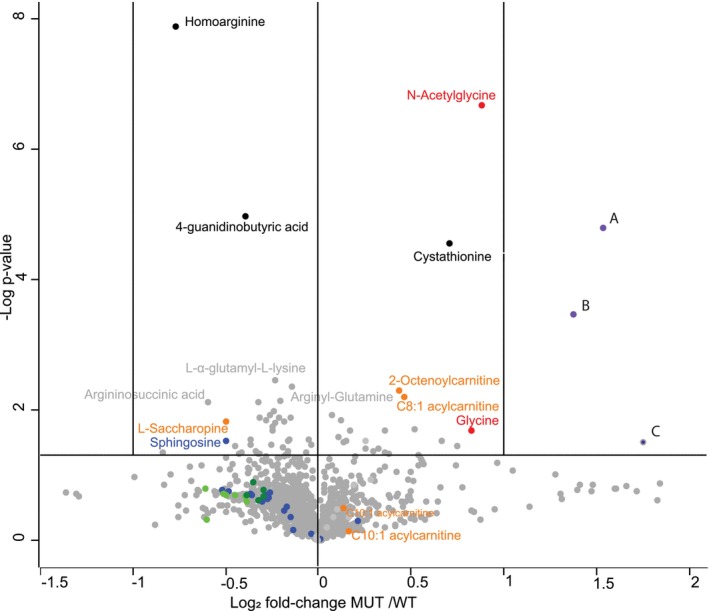
Metabolomics studies in the brain cortex comparing mice with nonketotic hyperglycinemia to wild type mice. The metabolomics studies in the brain cortex of young 5‐week‐old J129 mice with nonketotic hyperglycinemia compared with wild type mice are shown as a volcano plot. Metabolomics analysis shows an increase in glycine level with associated N‐acetylglycine (in red). The amounts of homoarginine and 4‐guanidinobutyrate are decreased, whereas cystathionine is increased (in black). Sphingosine and the sphingomyelins are decreased (in blue), and the ceramides are also decreased (in green). L‐saccharopine is decreased (orange), and octenoylcarnitine (and C8:1) carnitine is increased, but C10:1 acylcarnitine is not (orange). Three compounds' signatures (A, B, C) are markedly increased, but the structure is not known (Figure S7) (purple).

In the water‐soluble metabolomics (Figure 1), we observed an increase in glycine levels, as expected, and additionally an equal increase of N‐acetylglycine, reflecting the high amount of N‐acetylated amino acids in brain tissue. We observed an increase of cystathionine. Cystathionine is formed by cystathionine ß‐synthase by the condensation of homocysteine, which was normal, and L‐serine, which was decreased. It is further metabolized by cystathionine‐γ‐lyase to cysteine, which was unchanged, and α‐ketobutyrate (not identified), using the co‐factor pyridoxal‐phosphate, which was unchanged on metabolomics.

There was a decrease in 4‐guanidinobutyrate. This compound is formed by the enzyme arginine glycine amidinotransferase (*Agat*) using as substrate 4‐amino‐butyrate (GABA) instead of glycine. In this enzyme, the condensation of arginine with glycine competes with that of GABA, and we assume that increased glycine outcompetes GABA for this enzyme. We observed a decrease in homoarginine. Ornithine transcarbamylase condenses ornithine, a 5‐carbon amino acid, with carbamylphosphate to synthesize citrulline and from there to arginine. In this same enzyme reaction, lysine, a 6‐carbon amino acid homolog of ornithine, forms homocitrulline and from there homoarginine [53]. The decrease in homoarginine implies either decreased intra‐mitochondrial lysine or increased citrulline. Intra‐mitochondrial lysine is catabolized by lysine α‐ketoglutarate reductase (LKR) in the presence of NADPH to saccharopine, which was also reduced. This could imply reduce intra‐mitochondrial lysine or reduced NADPH flux. A substantial source of the reduction of intramitochondrial NADP^+^ to NADPH, equal or even greater than the pentose‐phosphate flux, is the oxidation of formyl‐tetrahydrofolate into carbon dioxide by formyl‐tetrahydrofolate dehydrogenase using formate units derived from serine or glycine, which is reduced in NKH [54]. The total cortex tissue NADPH/NADP+ ratio was reduced but not significantly (WT 6.00 (IQR 5.17–8.97, *N* = 13), MUT5.26 (IQR 3.44–7.12, *N* = 15), *p* = 0.363). Finally, an apparent mild increase in C8:1‐acylcarnitine was not replicated in a targeted analysis (Table S6). Only a mild decrease was detected in free carnitine, isovaleryl‐carnitine, C4‐hydroxy‐acylcarnitine, C5‐hydroxyacylcarnitines and C8‐hydroxyacylcarnitines, the biological significance of this is currently unclear.

**FIGURE 2:**
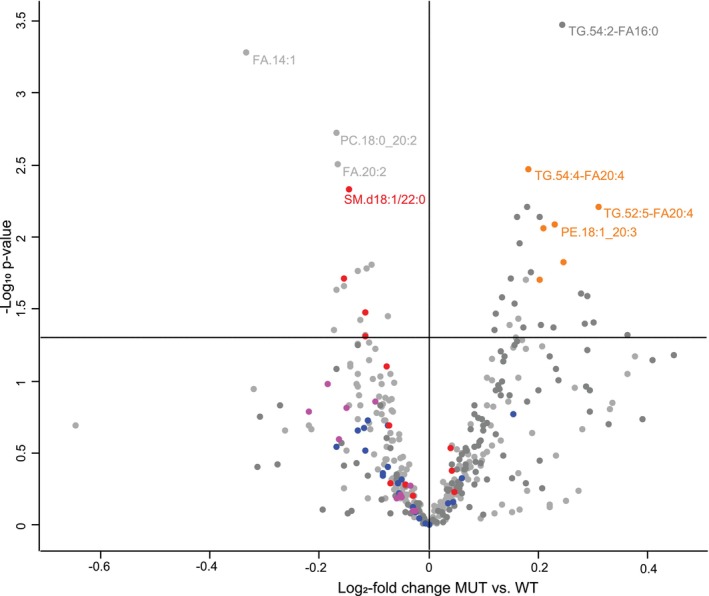
Lipidomics studies in the brain cortex comparing mice with nonketotic hyperglycinemia to wild type mice. The lipidomics studies in the brain cortex of young 5‐week‐old J129 mice with nonketotic hyperglycinemia compared with wild type mice are shown as a volcano plot. In this volcano plot of the lipidomics, the sphingomyelins are identified in red, the ceramides are identified in blue, and the lysophosphatidylethanolamines are identified in pink. These classes of metabolites are significantly decreased, most substantially for the sphingomyelins. In the metabolites that are increased, there are a few polyunsaturated triglycerides and phosphatidylethanolamines identified in orange.

The synthesis of sphingosine starts by the condensation of palmitoyl‐CoA with L‐serine, from which ceramides and sphingomyelins are formed [55, 56]. Metabolomics revealed decreased sphingosine, and multiple sphingosine metabolites and ceramides were all decreased (Figure 1A, blue and green, respectively). In lipidomics analysis, sphingomyelins were decreased (Figure 2). In a group analysis (Table 4), the sphingomyelin species and ceramide species were significantly decreased, but the glycosylated ceramides were unchanged. In contrast, the triglyceride class was increased. The most increased lipid species was TG.54:2‐FA16:0. Interestingly, there were multiple increased polyunsaturated fatty acids such as TG.54:4‐FA20:4 and TG52:5‐FA20:4 (Figure 2). Other lipid classes such as free fatty acids, phosphatidylcholines, phosphatidylethanolamines, and O‐alkyl‐phosphatidylethanolamines (plasmalogens) were unchanged, but lysophosphatidyl‐ethanolamines were decreased (Table 5). Sphingosine‐1‐phosphate, which has signaling properties, was unchanged. Phosphatidylserines are particularly enriched in the brain and are formed from phosphatidylcholines or from phosphatidylethanolamines by exchanging the choline or ethanolamine for serine through phosphatidylserine synthase 1 (*PTDSS1*) and 2 (*PTDSS2*), respectively [57]. Phosphatidylserines were not changed in the cortex of the MUT mice compared with WT mice.

**TABLE 4:** Comparison of lipid classes between WT and MUT mice with nonketotic hyperglycinemia in 5‐week‐old J129 mice brain cortex.

Metabolite classes	Number metabolites	WT mice		MUT mice		Paired testing	Change
		AVG ± SD	Median	AVG ± SD	Median	*t*‐test/Wilcoxon	
Sphingomyelins	12	2.69 ± 2.85	2.47	2.63 ± 2.86	2.33	**0.017**	9−/3+
Ceramides	9	1.80 ± 2.03	0.70*	1.72 ± 2.05	0.59*	0.015*	8−/1+
Hexosylceramides	12	3.91 ± 2.32	3.74	3.89 ± 2.27	3.77	0.395	8−/4+
Fatty acids	26	3.21 ± 2.52	2.68*	3.19 ± 2.53	2.67*	0.096*	18−/8+
Triglycerides	147	−1.25 ± 1.61	−1.46*	−1.18 ± 1.61	−1.41*	< 0.001*	40−/107+
Phosphatidylcholines	64	3.86 ± 2.77	3.54	3.85 ± 2.76	3.52	0.370	38−/26+
Phosphatidylethanolamines	45	2.06 ± 3.40	1.43	2.08 ± 3.41	1.54	0.206	22−/23+
Ether‐linked phosphatidylethanolamines	25	0.22 ± 3.15	−0.70*	0.22 ± 3.18	−0.79*	0.977*	13−/11+
Cholesterol esters	6	0.21 ± 1.10	0.16	0.38 ± 0.97	0.44	0.058	2−/4+
Diacylglycerols	5	0.75 ± 1.48	0.39*	0.73 ± 1.49	0.31*	0.893*	2−/3+
Phosphatidylethanolamine plasmalogens	43	3.14 ± 3.05	2.70	3.14 ± 3.09	2.67	0.915	23−/20+
Lyso‐phosphatidylethanolamines	11	0.009 ± 2.306	−0.937	−0.878 ± 2.319	−1.121	**0.001**	11−/0+
Lyso‐phosphatidylcholines	6	2.07 ± 2.50	2.30	1.92 ± 2.71	2.26	0.229	4−/2+
Phosphatidylserines	64	18.54 ± 1.49	18.528	18.58 ± 1.46	18.616	0.306	29−/35+

*Note:* The various lipid classes were compared between 5‐week‐old wild type (WT) and homozygous mutant (MUT) mice in the brain cortex. Values were compared after Log2(x) transformation with average (AVG) and standard deviation (SD) and Median shown. Comparisons were done by paired Student *t*‐test and by Wilcoxon‐signed‐rank test, with significant differences highlighted in bold. Populations that deviated from the normal distribution are marked with an asterisk*. In each group, the number of analytes that were decreased (−) or increased (+) are given when comparing the MUT animals to the WT animals. Significant decrease was found for the sphingomyelins, ceramides and lyso‐phosphatidylethanolamines, and significant increase for the class of the triglycerides. Significant *p*‐values are highlighted in bold.

**TABLE 5:** Comparison of core metabolites in young 5‐week‐old C57Bl/6J mice comparing mutant mice versus wild type mice.

B6 5 weeks old	WT mice *N* = 14		MUT mice *N* = 9		Analysis		Change
Analyte	Mean ± SD	Median (IQR)	Mean ± SD	Median (IQR)	*p*‐value *t*‐test or MWU	Benjamini‐Hochberg *p*‐value	% change
Plasma analytes in μmol/L							
Glycine	436 ± 97*	430 (401–440)	910 ± 218	1023 (711–1101)	< 0.001*	**< 0.01**	**+108%**
L‐serine	125 ± 17*	123 (115–131)	115 ± 20	122 (102–129)	0.439*	0.488	−8%
Methionine	43.2 ± 9.3	44.9 (34.2–49.7)	33.6 ± 12.4	30.0 (34.2–49.7)	**0.045**	0.150	−22%
Homocysteine	6.14 ± 2.90	5.81 (3.66–7.58)	6.12 ± 1.98	6.04 (4.53–7.93)	0.985	0.985	0%
Cysteine	190 ± 28	184 (171–218)	157 ± 29	150 (134–182)	**0.011**	0.055	−17%
Cysteinylglycine	2.67 ± 0.44	2.69 (2.40–3.00)	2.50 ± 0.46*	2.74 (2.13–2.90)	0.439*	0.488	−6%
Threonine	113 ± 21	114 (90–135)	103 ± 19	101 (88–118)	0.248	0.488	−9%
Glutamate	48.6 ± 10.2	48.0 (39.2–52.9)	44.8 ± 11.6	47.3 (39.1–52.5)	0.412	0.188	−8%
Glutamine	640 ± 66	651 (589–693)	618 ± 54	617 (585–646)	0.413	0.488	−3%
Glutathione	78.7 ± 14.8	70.1 (54.7–79.7)	70.0 ± 15.5	70.1 (57.6–79.7)	0.192	0.480	−11%
Cortex analytes in nmol/g							
Glycine	973 ± 165	913 (835–1065)	1901 ± 386*	1777 (1673–2008)	< 0.001*	**0.011**	**+95%**
L‐serine	569 ± 54	557 (522–623)	587 ± 40*	578 (561–592)	0.369*	0.812	+3%
D‐serine	241 ± 28	247 (216–264)	235 ± 12	236 (230–244)	0.452	0.829	−2%
Methionine	49.3 ± 8.7	47.7 (40.2–57.8)	47.0 ± 31.1*	37.7 (29.5–55.0)	0.096*	0.385	−5%
Homocysteine	2.18 ± 0.45	2.05 (1.80–2.55)	2.24 ± 0.23	2.30 (2.05–2.40)	0.690	0.859	+3%
Cysteine	55.6 ± 5.3	55.8 (53.7–60.2)	54.7 ± 9.7	55.4 (48.9–60.8)	0.758	0.859	−2%
Cysteinylglycine	2.03 ± 0.18	2.05 (1.87–2.20)	2.26 ± 0.39	2.20 (1.95–2.60)	0.135	0.385	+11%
Glutamate #	16.88 ± 0.96	16.76 (16.07–17.79)	17.03 ± 2.28	17.17 (15.75–18.94)	0.859	0.859	+1%
Glutamine #	4.53 ± 0.21	4.50 (4.39–4.63)	5.04 ± 0.91	5.06 (4.25–5.77)	0.140	0.385	+11%
Glutathione	508 ± 199*	384 (368–711)	560 ± 188	628 (369–741)	0.557*	0.859	+10%
Hippocampus analytes in nmol/g							
Glycine	1022 ± 57	1016 (975–1048)	2134 ± 254	2115 (2004–2307)	**< 0.001**	**< 0.011**	**+109%**
L‐serine	606 ± 35	601 (583–640)	607 ± 60	616 (560–658)	0.962	0.962	0%
D‐serine	220 ± 12	220 (217–229)	204 ± 23*	215 (183–221)	0.046*	0.218	−7%
Methionine	67.6 ± 7.8	67.5 (62.1–72.9)	60.7 ± 10.9	61.0 (50.5–72.3)	0.093	0.218	−10%
Homocysteine	3.46 ± 0.20	3.44 (3.32–3.59)	3.55 ± 0.40	3.64 (3.31–3.85)	0.495	0.598	+3%
Cysteine	77.8 ± 5.8	77.2 (73.7–81.4)	82.8 ± 7.2	81.3 (76.3–88.0)	0.079	0.218	+6%
Cysteinylglycine	2.35 ± 0.21	2.40 (2.19–2.53)	2.43 ± 0.29	2.42 (2.25–2.55)	0.491	0.598	+3%
Threonine	207 ± 41	203 (170–244)	218 ± 46	218 (178–252)	0.544	0.598	+5%
Glutamate #	10.34 ± 0.54*	10.36 (10.19–10.81)	10.22 ± 1.45*	10.83 (9.77–11.19)	0.369*	0.598	−1%
Glutamine #	5.31 ± 0.33	5.37 (5.04–5.56)	5.63 ± 0.54	5.54 (5.27–5.92)	0.099	0.218	+6%
Glutathione	616 ± 59	612 (565–655)	616 ± 69*	643 (601–656)	0.516*	0.598	0%
Cerebellum analytes in nmol/g							
Glycine	1126 ± 82	1133 (1082–1189)	3329 ± 519	3409 (2849–3797)	**< 0.001**	**< 0.0025**	**+196%**
L‐serine	469 ± 43	477 (441–498)	569 ± 80	563 (491–634)	**< 0.001**	**< 0.0025**	**+21%**
Methionine	74.7 ± 8.8	74.2 (70.1–79.5)	65.7 ± 11.1	66.5 (54.0–75.1)	**0.041**	0.082	−12%
Homocysteine	2.43 ± 0.23	2.46 (2.25–2.60)	3.04 ± 0.34*	2.85 (2.83–3.35)	< 0.001*	**< 0.0025**	**+25%**
Cysteine	110.3 ± 18.5	113.2 (90.4–121.9)	117.9 ± 22.4	120.0 (100.8–132.2)	0.388	0.485	+7%
Cysteinylglycine	2.48 ± 0.33	2.45 (2.16–2.69)	2.65 ± 0.34	2.59 (2.49–2.88)	0.249	0.356	+7%
Threonine	334 ± 51	325 (291–381)	495 ± 85	477 (427–555)	**< 0.001**	**< 0.0025**	**+48%**
Glutamate #	9.93 ± 0.67	10.12 (9.48–10.37)	9.71 ± 0.66	9.67 (9.25–10.05)	0.446	0.496	−2%
Glutamine #	7.11 ± 0.53	7.01 (6.90–7.37)	7.45 ± 0.69	7.45 (7.01–8.06)	0.200	0.333	+5%
Glutathione	628 ± 58	632 (577–685)	638 ± 49	651 (612–659)	0.677	0.677	+2%

*Note:* Values of metabolites in C576Bl6/J mice at age 5 weeks comparing wild type (WT) and homozygous mutant (MUT) animals. The distribution of values is shown as mean (AVG) and standard deviation (SD) and as median and interquartile range (IQR). Values of metabolites in plasma are in μM, and values of the metabolites in forebrain cortex, hippocampus and cerebellum are shown in nmol/g tissue (# except for glutamate and glutamine shown in μmol/g tissue). There were 14 WT and 9 MUT animals. A significant deviation of the normal distribution is indicated by an asterisk*. Comparisons are done by Student *t*‐test, or by Mann–Whitney‐U test if any of the two populations deviates from the normal distribution as indicated by an asterisk*, with *p*‐values shown. A Benjamini‐Hochberg corrected *p*‐value is provided for the multiple comparisons within each tissue type. Significant *p*‐values are highlighted in bold. The percentage differences between the MUT in comparison with the WT is given with green values for significant increase and red values for a significant decrease.

On PCA analysis of overall lipidomics, MUT and WT mice did not separate (Figure S5A). Lipid ontology enrichment analysis showed an increase in neutral triglycerides and glycerolipid classes of neutral headgroup, which related to increased lipid storage and lipid droplets (Figure S5B). There was some underrepresentation of lower unsaturated species of C20:3, C16:1, and C22:2. The changes in the lipid networks and fatty acid networks are shown in Figure S6. The most marked changes in lipid networks included both a shift from phosphatidylcholines to lyso‐phosphatidylcholines and from phosphoethanolamines to lyso‐phosphoethanolamines (Figure S6A), which correspond with the underrepresentation of the lyso classes in our group analysis. Interestingly, it also indicates a shift from ceramides towards sphingolipids, although both classes had been reduced on group analysis. In the fatty acid network, there tends to be a shift towards long‐chain fatty acids (C22 and C24) and towards polyunsaturated fatty acids (Figure S6B). These fatty acids were also recognized on the volcano plot. They tend to be located in neurons and myelin sheets [58].

Finally, the metabolomics study revealed three unidentified strongly upregulated species, with mass spectra shown in Figure S7.

### 3.4 Proteomics

**FIGURE 3:**
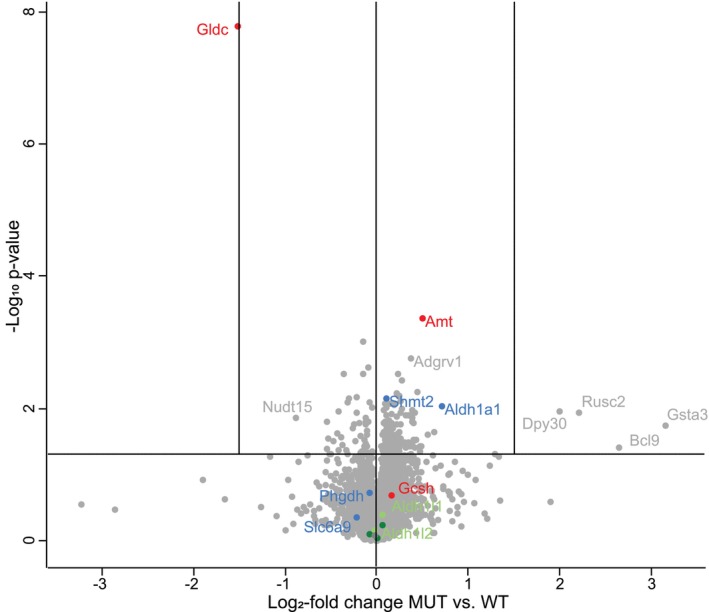
Proteomics studies in the brain cortex comparing mice with nonketotic hyperglycinemia to wild type mice. The proteomics studies in the brain cortex of young 5‐week‐old J129 mice with nonketotic hyperglycinemia compared with wild‐type mice are shown as a volcano plot. Of the glycine cleavage enzyme (marked in red), the Gldc protein is decreased as expected, and the Amt protein is mildly increased, whereas the Gcsh protein is unchanged. The glycine transporter Slc6a9 (Glyt1) is unchanged (blue), and the Shmt2 enzyme level is relatively subtly but statistically significantly increased, whereas the controlling enzyme for the serine synthesis Pgdh is unchanged (in blue). All enzymes in the folate pathway are unchanged including the 10‐formyltetrahydrolate dehydrogenase enzymes Aldh1l1 and Aldh1l2 (in green). Some increased enzymes such as glutathione S‐α‐3 (Gasta3) had only mild significance. The aldehyde dehydrogenase 1A1 (Aldh1a1, in blue) is also mildly increased.

Evaluating the GCS components in the proteomics profile in the same cortex specimens (Figure 3) revealed, as expected, decreased P‐protein (Gldc) at 34.8% of controls (*p* < 10^−7^), with mildly increased T‐protein (Amt) at 141%, and unchanged amount of H‐protein (Gcsh). The GCS resides primarily in astrocytes, and the astrocyte marker protein Gfap was unchanged. The mitochondrial serine hydroxymethyltransferase (Shmt2), which has a key impact on serine and folate metabolism, was barely increased. Equally, the formyl‐THF dehydrogenase enzyme mitochondrial (Aldh1l2) and cytosolic (Aldh1l1) were unchanged, and all other folate interconverting enzymes and the folate transporter proteins were unchanged, as were serine synthesis enzymes (Phgdh, Psat1, Psph). The folate transporter Folr1 was incompletely covered, but on Western blot was unchanged. The protein level of the glycine transporter Glyt1 (Slc6a9) was unchanged on proteomics. The transporter that regulates brain glycine by extrusion, Slc6a20 [59], is primarily expressed on brain endothelial cells including in the choroid plexus and meninges [60, 61], and also on astrocytes and neurons [54]. This glycine/proline transporter was doubled in abundance by Western blot (Figure S8). There was no change in the amino acid transporter Slc25a15 (a.k.a Ornt1), which transports lysine into mitochondria. Important to the 2‐aminoacetate pathway, the proteins Gcat and Tdh were unchanged by Western blot.

Despite increased methylglyoxal, its detoxifying enzyme Glo1 is unchanged. Four proteins showed apparent increases at low significance (0.05 > *p* > 0.01): GAST3, BCL9, RSC2, and DPY30. Validation analysis by western blot did not confirm a significant increase for three of these proteins, including GAST3 (glutathione S‐transferase α‐3, which converts lipid peroxides to glutathione conjugates and an anti‐oxidant protease [62, 63]), BCL9 (B‐cell CLL/lymphoma 9 protein), and RUSC2 (Iporin, isoform 2). The DPY30 protein (dpy‐30 homolog) is a subunit of mammalian COMPASS complexes. In nuclear extracts using HDAC1 as control, DPY30 was increased in MUT (0.68 ± 0.22) compared with WT (0.29 ± 0.12), *p* = 0.003 (Figure S9). DPY30 provides the backbone of SET1/MLL complexes, which are involved in histone 3 lysine 4 methylation (H3K4) [64]. It interacts directly with AAH2L and indirectly with WDR5 and RBBP5 [64]. Since DPY30 does not itself interact with the catalytically active proteins, these interacting proteins should equally be increased if increased DPY30 results in more active complexes, but this was not the case: ASH2L MUT = 105% of WT, *p* = 0.43; RBBP MUT = 99.7% of WT, *p* = 0.96; and WDR5 MUT = 103% of WT, *p* = 0.45 (all proteins well covered). DPY30 also interacts with AKAP95 (a.k.a. AKAP8) [64], which was also unchanged: MUT = 100% of WT, *p* = 0.965; and with BIG1, which was also unchanged: MUT = 99% of WT, *p* = 0.72. Thus, in this explorative proteomics analysis, these proteins changed at low significance did not provide replication of a significant change on orthogonal analyses. Ingenuity pathway analysis after removal of low variance proteins did not identify any causative pathway that was significantly changed.

### 3.5 Mitochondrial Enzyme Activities

We explored whether the changes in the NKH mice would affect the respiratory chain enzyme activities. No changes were identified between the MUT and WT mice (Table S7). On proteomics analysis, the proteins residing in the mitochondria, as annotated from the mitocarta 3.0 database, were mildly upregulated (Figure S10), but when corrected for total mitochondrial abundance, the respiratory chain complex abundances and mitoribosomal subunits were normal (Figure S11). Mitochondrial upregulation can be linked to oxidative stress through Nrf2, but there was no evidence of Nrf2 activation by its nuclear translocation (Figure S12).

### 3.6 Strain Differences

We evaluated if there were strain‐specific differences by comparing these findings in the B6 mice (Table 5). In B6 NKH mice plasma glycine levels were elevated (+108%) and methionine was decreased. In the cerebellum, the increase in glycine (+196%), L‐serine (+21%), and threonine (+48%) was very similar to the J129 findings. However, in the cortex and hippocampus, although there was a similar elevation of glycine by 95% and 109%, respectively, there was no significant decrease in L‐serine or D‐serine in the cortex or hippocampus, and also no significant decrease in methionine in the cortex. Despite no decrease in methionine, the folate profile showed a decrease in 5‐methyl‐tetrahydrofolate. The changes in serine metabolism were not replicated in this strain. Comparing metabolites between the two strains (Table S8), glycine levels were higher in the B6 mice in plasma and in the cortex than in the J129 mice in both WT and MUT. The L‐serine and D‐serine were higher in the cortex of the WT J129 mice than in the WT B6 mice, which may in part explain the differences in the effect of the mutation. Homocysteine, cysteine, and cysteinylglycine levels were lower in WT and MUT B6 mice than in J129 mice.

### 3.7 Glycine Loading

We have shown previously that challenging mice by providing 5% glycine in drinking water increased glycine levels and was associated with growth failure and increased mortality [22]. Here we expand these biochemical observations to core glycine related metabolites.

In WT mice (Table S9), glycine supplementation resulted in increased plasma glycine (+318%), serine (+38%), and homocysteine (+51%). In cortex, there was an increase in glycine (+59%), L‐serine (+53%), and D‐serine (+55%), with a mild increase in glutamine, whereas methionine (−24%) decreased. In hippocampus, this similarly resulted in increases in glycine, L‐serine, and D‐serine but only by 20%, whereas methionine (−48%) decreased more. In the cerebellum, there was an increase in glycine (+23%), and large increases in L‐serine (+111%) and homocysteine (+18%), whereas methionine (−18%) decreased. Thus, in all regions, glycine, serine, and homocysteine increased, and methionine decreased.

In MUT J129 mice (Table 6), these changes were more pronounced. In plasma, glycine increased 11‐fold, and homocysteine levels increased a dramatic 24‐fold, with smaller increases in threonine (+37%) and glutamine (+34%), and a decrease in glutathione (−22%). In liver, homocysteine doubled and methionine halved. In the cortex, glycine increased (+186%), as did L‐serine (+82%) and D‐serine (+29%). In contrast to plasma, in the cortex there was no change in either homocysteine or methionine, and we only observed increased glutamine (+156%) and glutathione (+23%). Similarly, in the hippocampus, glycine (+85%) and L‐serine (+44%) increased, but D‐serine did not change. There was a very mild increase in homocysteine (+26%) and a decrease of methionine (−45%) and cysteine (−31%). Here too glutamine increased by 1.3‐fold with only a small increase in glutamate. In cerebellum, both glycine and L‐serine doubled, with a moderate increase in homocysteine by 66% and a methionine decrease by 31%. There was a more moderate increase in glutamine by 43%.

**TABLE 6:** Impact of glycine 5% challenge on metabolites in young 5‐week‐old homozygous mutant J129 mice with nonketotic hyperglycinemia.

J129 5 weeks old	No treatment *N* = 14		Treatment *N* = 14		Comparison treated vs. untreated mutant mice			Comparison treated MUT to normal mice‡	
Metabolite	AVG ± SD	Median IQR	AVG ± SD	Median IQR	*p*‐value *t*‐test/MWU	BH *p*‐value	% change	% of normal	*p* vs. normal
Plasma analytes in μmol/L									
Glycine	695 ± 175	722 (533–805)	8756 ± 5035*	7834 (5704–8796)	< 0.001*	**< 0.0025**	**+1160%**	**+2522%**	< 0.001*
L‐serine	129 ± 37	123 (95–171)	149 ± 31	154 (120–177)	0.137	0.196	+16%	+7%	0.410
Methionine	32.7 ± 7.7	30.6 (26.0–41.6)	30.3 ± 12.1	28.1 (18.4–40.3)	0.535	0.669	−7%	**−26%**	0.022*
Homocysteine	4.76 ± 1.75	4.39 (3.62–6.12)	119.6 ± 24.4	115.9 (105.1–135.6)	**< 0.001**	**< 0.0025**	**+2400%**	**+2341%**	**< 0.001**
Cysteine	132.7 ± 42.4	123.3 (105.5–164.9)	126 ± 11*	123 (119–134)	0.946	0.946	−5%	−2%	0.603*
Cysteinylglycine	3.13 ± 0.91	3.47 (2.26–3.83)	1.33 ± 0.19	1.29 (1.21–1.51)	**< 0.001**	**< 0.0025**	**−58%**	**−68%**	**< 0.001**
Threonine	101 ± 30	88 (79–119)	139 ± 51*	138 (120–177)	0.008*	**0.0133**	**+37%**	**+21%**	0.034*
Glutamate	45.2 ± 10.2	45.5 (35.7–50.5)	45.7 ± 11.3	43.8 (39.7–52.8)	0.906	0.946	+1%	−6%	0.530
Glutamine	578 ± 83	557 (501.9–653)	776 ± 98	775 (720–816)	**< 0.001**	**< 0.0025**	**+34%**	**+27%**	**< 0.001**
Glutathione	67.9 ± 13.6	69.8 (59.7–78.0)	52.9 ± 13.2	53.2 (40.3–65.7)	**0.006**	**0.012**	**−22%**	**−29%**	< 0.001*
Cortex analytes in nmol/g									
Glycine	1581 ± 175*	1583 (1458–1733)	4518 ± 690*	4225 (3916–5299)	< 0.001*	**< 0.0027**	**+186%**	**+428%**	< 0.001*
L‐serine	565 ± 64	575 (546–598)	1030 ± 212	977 (837–1212)	**< 0.001**	**< 0.0027**	**+82%**	**+43%**	**< 0.001**
D‐serine	238 ± 32	244 (226–258)	307 ± 61*	315 (258–338)	0.002*	**0.0044**	**+29%**	+2%	0.608*
Methionine	53.5 ± 15.5*	53.2 (41.4–64.0)	52.9 ± 27.6	45.1 (38.5–62.1)	0.454*	0.624	−1%	**−25%**	0.001*
Homocysteine	3.54 ± 0.44*	3.65 (3.20–3.85)	3.61 ± 0.36	3.63 (3.36–3.87)	0.804*	0.812	+2%	−5%	0.094*
Cysteine	123.1 ± 13.3	123 (117–136)	112.1 ± 12.4	108.9 (101.9–120.9)	**0.032**	0.0587	−9%	+1%	0.946
Cysteinylglycine	3.69 ± 0.80	3.45 (2.97–4.30)	3.44 ± 0.23	3.44 (3.21–3.64)	0.266	0.418	−7%	**+22%**	**0.008**
Glutamate #	16.4 ± 1.7	16.7 (15.5–17.7)	16.5 ± 0.9	16.7 (15.7–17.1)	0.635	0.812	+1%	−2%	0.154*
Glutamine #	4.53 ± 1.43	4.04 (3.81–4.87)	11.59 ± 4.68*	10.17 (7.7–15.4)	< 0.001*	**< 0.0027**	**+156%**	**+159%**	< 0.001*
Glutathione	544 ± 67*	519 (484–596)	670 ± 40	673 (636–698)	< 0.001*	**< 0.0027**	**+23%**	**+20%**	**0.009**
Hippocampus analytes in nmol/g									
Glycine	2062 ± 162	2057 (1899–2180)	3819 ± 385	3823 (3543–4198)	**< 0.001**	**< 0.0014**	**+85%**	**+311%**	**< 0.001**
L‐serine	669 ± 40	668 (633–700)	965 ± 185	924 (866–1116)	**< 0.001**	**< 0.0014**	**+44%**	**+34%**	**< 0.001**
D‐serine	232 ± 20	228 (211–250)	223 ± 41	215 (189–255)	0.463	0.549	−4%	**−19%**	< 0.001*
Methionine	81.3 ± 20.3	80.5 (62.6–101.3)	45.1 ± 8.5	44.9 (41.0–48.0)	**< 0.001**	**< 0.0014**	**−45%**	**−46%**	**< 0.001**
Homocysteine	3.08 ± 0.67	3.23 (2.33–3.71)	3.88 ± 0.44	3.88 (3.61–4.20)	**0.001**	**0.0014**	**+26%**	**+25%**	**< 0.001**
Cysteine	97.9 ± 26.7	92.8 (78.9–119.4)	67.6 ± 8.8*	64.2 (62.1–74.7)	< 0.001*	**< 0.0014**	**−31%**	**−39%**	< 0.001*
Cysteinylglycine	4.52 ± 1.33	4.01 (3.30–5.96)	4.77 ± 0.35	4.62 (4.54–5.00)	0.499	0.549	−30%	+9%	0.250
Threonine	338 ± 47	350 (297–371)	237 ± 51	237 (198–261)	**< 0.001**	**< 0.0014**	**−30%**	**−29%**	**< 0.001**
Glutamate #	10.13 ± 0.79	10.29 (9.41–10.81)	11.88 ± 1.10	12.04 (10.98–12.84)	**< 0.001**	**< 0.0014**	**+17%**	**+18%**	**< 0.001**
Glutamine #	4.86 ± 1.57*	4.32 (3.99–5.07)	11.16 ± 3.98	11.03 (7.05–15.04)	< 0001*	**< 0.0014**	**+127%**	**+142%**	< 0.001*
Glutathione	498 ± 90	492 (403–591)	511 ± 49	517 (472–550)	0.623	0.623	+3%	+4%	0.488
Cerebellum analytes in nmol/g									
Glycine	3218 ± 301	3212 (3040–3370)	6473 ± 803	6735 (5808–6874)	**< 0.001**	**< 0.002**	**+101%**	**+606%**	**< 0.001**
L‐serine	621 ± 71	632 (555–672)	1266 ± 405*	1130 (906–1621)	< 0.001*	**< 0.002**	**+104%**	**+131%**	< 0.001*
Methionine	79.5 ± 15.5	75.6 (70.1–86.6)	54.7 ± 7.4	52.3 (50.7–61.3)	**< 0.001**	**< 0.002**	**−31%**	**−36%**	**< 0.001**
Homocysteine	2.46 ± 0.18	2.48 (2.36–2.58)	4.09 ± 0.45*	427 (3.54–4.44)	< 0.001*	**< 0.002**	**+66%**	**+62%**	< 0.001*
Cysteine	85.7 ± 15.3	81.5 (70.9–99.3)	94.3 ± 12.5	96.1 (83.6–100.7)	0.118	0.1686	+10%	+13%	0.140
Cysteinylglycine	7.26 ± 0.49	7.17 (6.82–7.67)	7.05 ± 0.27	7.11 (6.90–7.21)	0.178	0.2225	−3%	+1%	0.583
Threonine	519 ± 111	526 (444–587)	523 ± 83	535 (446–584)	0.929	0.929	+1%	**+37%**	< 0.001*
Glutamate #	10.10 ± 0.42*	10.26 (9.78–10.45)	9.14 ± 0.75	9.12 (8.58–9.49)	< 0.001*	**< 0.002**	**−10%**	**−8%**	**0.002**
Glutamine #	6.97 ± 1.62*	6.50 (6.08–7.16)	9.95 ± 3.73	8.92 (6.71–12.93)	0.019*	**0.0317**	**+43%**	**+53%**	0.002*
Glutathione	636 ± 40	642 (617–668)	616 ± 67	630 (564–670)	0.365	0.406	−3%	**−12%**	< 0.001*
Liver analytes in nmol/g									
Glycine	4519 ± 771	4546 (3719–5217)	48 120 ± 13 224	44 568 (38142–61 195)	**< 0.001**	**< 0.0013**	**+965%**	**+2450%**	**< 0.001**
L‐serine	386 ± 50	371 (351–428)	295 ± 62	298 (254–332)	**< 0.001**	**< 0.0013**	**−24%**	**−27%**	**< 0.001**
Methionine	128.1 ± 14.7	124.5 (114.4–141.0)	73.3 ± 36.6*	64.9 (48.5–76.9)	< 0.001*	**< 0.0013**	**−43%**	**−38%**	< 0.001*
Homocysteine	5.13 ± 0.79	5.25 (4.33–5.60)	9.67 ± 2.54	8.72 (8.04–11.50)	**< 0.001**	**< 0.0013**	**+88%**	**+60%**	**< 0.001**
Cysteine	470 ± 40	469 (430–513)	383 ± 66*	373 (331–447)	< 0.001*	**< 0.0013**	**−19%**	**−27%**	< 0.001*
Cysteinylglycine	14.30 ± 2.10	14.72 (12.81–15.60)	5.13 ± 0.79	7.92 (7.48–8.67)	**< 0.001**	**< 0.0013**	**−64%**	**−65%**	**< 0.001**
Glutamate #	1.25 ± 0.36	1.33 (0.88–1.47)	0.915 ± 0.408*	0.831 (0.526–1.301)	0.056	0.056	**−**27%	**−26%**	0.031*
Glutamine #	5.40 ± 0.86	5.45 (4.84–6.15)	3.07 ± 1.69	2.63 (1.93–4.45)	**< 0.001**	**< 0.0013**	**−43%**	**−48%**	**< 0.001**
Glutathione	2335 ± 566	2242 (1913–2847)	1694 ± 422	1578 (1414–2007)	**0.002**	**0.0022**	**−27%**	**−20%**	**0.039**

*Note:* Values of metabolites in J129 mice at age 5 weeks are shown comparing homozygous mutant mice without treatment to homozygous mutant mice treated with glycine 5% in drinking water administered for 1 week. The distribution in the untreated and the treated mice is shown as mean (AVG) and standard deviation (SD). Values of metabolites in plasma are in μM, and values of the metabolites in forebrain cortex, hippocampus and cerebellum are shown in nmol/g tissue (# except for glutamate and glutamine shown in μmol/g tissue). There were 14 untreated and 14 treated mice. A significant deviation of the normal distribution is indicated by an asterisk*. Comparisons are done by Student *t*‐test or by Mann–Whitney‐U test if any of the two populations deviated from the normal distribution as indicated by an asterisk*, and *p*‐values shown. A Benjamini‐Hochberg corrected *p*‐value is provided for the multiple comparisons within each tissue type. Significant *p*‐values are highlighted in bold. The percentage differences between the untreated and the treated mice is given with green values for significant increase and red values for a significant decrease. In the last two columns the treated mutant mice are compared with baseline untreated wild type mice‡ (with data shown in Table 1) and the percentage change of this comparison is given with statistically significant increases given in green and decreases in red, and the *p*‐value of this comparison given from either the Student *t*‐test of the Mann–Whitney‐U test if not normally distributed indicated by an asterisk*.

The observed changes with glycine loading indicate that excess glycine is converted to increased L‐serine, which in the cortex resulted in increased D‐serine. Since the increase in D‐serine was larger in WT than in MUT mice, following glycine loading the D‐serine was even more decreased in MUT mice compared with WT mice: cortex MUT 307 ± 61 vs. WT 469 ± 51, *p* < 0.001 –35% (vs. a decrease of −21% without glycine loading), hippocampus MUT 223 ± 41 vs. WT 298 ± 28, *p* < 0.001, −25% (vs. a decrease of −15% without glycine loading). These results also indirectly indicate that the folate one‐carbon charging worsened, resulting in increased homocysteine and decreased methionine, more so in cerebellum than in cortex or hippocampus, but most dramatically so in blood, even more than in liver. The minor changes present in normal mice were strongly exaggerated in mutant mice.

## 4 Discussion

**FIGURE 4:**
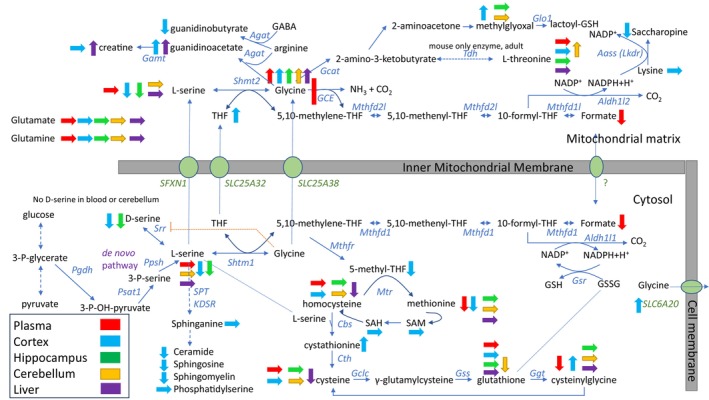
Overview of metabolism changes in 5‐week‐old J129 mice comparing nonketotic hyperglycinemia MUT mice with WT mice. The diagram illustrates the core metabolism of glycine and the changes identified when comparing mutant mice for nonketotic hyperglycinemia with wild type mice. The changes in blood are indicated by a red arrow, in cortex by a blue arrow, in hippocampus by a green arrow, in cerebellum by an orange arrow, and in liver by a purple arrow. A vertical up arrow indicates a significant increase in mutant mice, a down arrow indicates a significant decrease in mutant mice, and a horizontal arrow indicates no significant change was observed. The enzymes are identified by the gene name, except for the multiprotein enzyme complexes GCE = glycine cleavage enzyme, and SPT = serine palmitoyltransferase. GABA, γ‐aminobutyrate; GSH, reduced glutathione; GSSG, oxidized glutathione; THF, tetrahydrofolate. Serine racemase is only expressed in cortex and hippocampus and is absent in cerebellum; hence, D‐serine was not identified in cerebellum. The glycine transporter Slc6a20 is present on the cell membrane.

In this study, we document a range of metabolic changes induced by the deficiency of glycine cleavage enzyme activity in brain, the origin of key symptomatology in NKH, and which were often different from blood (Figure 4). Theoretically, absent GCS activity results in accumulation of its substrate glycine, and insufficiency of its end‐product 5,10‐methylene‐tetrahydrofolate.

Indeed, excess glycine accumulated in all three brain regions regardless of sex or age, and was highest in cerebellum similar to observations in human patients [13, 65, 66]. There was increased abundance of the glycine transporter Slc6a20, which is known to decrease cerebral glycine levels and mitigate glycine increase [59]. Excessive glycine has a neurotoxic effect [67, 68], but the mechanism has not been clarified. Neurophysiologically, glycine is an activator at the allosteric site of the NMDA‐type glutamatergic receptor [69], an agonist at the NR1/NR3 type glutamatergic receptors [70], and on glycinergic receptors which are widespread in the brain cortex mostly in the fetal period [71]. We also identified several glycine‐derived compounds of which two were neurotoxic: guanidinoacetate and methylglyoxal. Methylglyoxal, which markedly accumulated specifically in brain cortex, has known neurotoxicity [66, 67, 68, 72, 73, 74]. In leukemic cells, clearance of glycine is essential for survival due to excessive accumulation of aminoacetone and methylglyoxal, illustrating a direct significance in human cell biology [75, 76]. Ketone bodies like acetoacetate can mitigate methylglyoxal accumulation and its negative effect, including during ketogenic diet [77, 78], which adds a new benefit to this therapeutic modality in NKH [8]. In the presence of normal amount of arginine: glycine amidinotransferase (Agat), increased glycine by substrate effect formed increased guanidinoacetate in brain and liver [16], outcompeting an alternate substrate γ‐aminobutyrate with decreased guanidinobutyrate. Guanidinoacetate is a potent GABA_A_ receptor agonist [79], is neurotoxic, and its accumulation has been linked to cognitive dysfunction, movement disorder, and epilepsy in the genetic disorder of guanidinoacetate methyltransferase (*Gamt*) deficiency [80]. An increase in γ‐glutamylglycine, formed by γ‐glutamyltransferase (*Ggt*), in brain has been noted in another NKH mouse model [16].

Acetylated amino acids are prevalent in brain, and we identified a strong increase in N‐acetylglycine. N‐acetylglycine can be formed in the liver by the glycine‐N‐acyltransferase (*Glyat1*) and is present in blood and urine together with other acylglycines in another NKH mouse model [16]. It may also be locally synthesized in brain from N‐acetylated amino acids on brain proteins [81]. No direct toxicity was noted for N‐acetylglycine when provided enterally [82]. Glycine is an inhibitor of serine racemase [15, 83], and indeed a greater decrease of D‐serine was found in cortex and hippocampus than the decrease in its precursor L‐serine, similar to findings in human patients [14, 15]. D‐serine has neurotransmitter roles as activator of the allosteric activation site of the NMDA‐type glutamatergic receptor and as direct agonist on the NR1/NR3 type glutamatergic receptors [9]. There is a strain effect, and the young J129 mouse without glycine loading biochemically best approached the enantiomeric serine findings in human NKH patients [22]. Since these metabolite changes are flux and concentration derived in brain, it appears likely that reducing brain glycine levels may result in reduction in methylglyoxal, guanidinoacetate, N‐acetylglycine, γ‐glutamylglycine, and reverse the decrease in D‐serine and guanidinobutyrate. This emphasizes reducing brain glycine levels as a key therapeutic target.

The decreased production of 5,10‐methylenetetrahydrofolate in NKH had several biochemical consequences. Folate species can interconvert, and as previously shown in GLDC‐deficient mouse embryos [17, 18] and brain [12], the decrease was most evident in the reduced relative amount of 5‐methyltetrahydrofolate and increased relative amount of uncharged tetrahydrofolate [17, 18]. This primarily resulted in a decrease in methionine in brain tissues but, surprisingly, without changes in homocysteine, SAM, or SAH. This effect was more pronounced in the cortex than in the hippocampus, and was not noted in the cerebellum. Methionine significantly increased with age resolving its deficiency in adult animals. These changes correlate with the age‐dependent clinical effect of NKH. In our mouse model, we showed a growth retardation most present after weaning at age 5 weeks, and recovering in adult mice, similar to the level of methionine [22]. In human patients with NKH, a similar specific early vulnerable period has also been proposed based on clinical observation [84], and a greater effect of treatment has been noted in infancy compared with later age [10, 85]. Primary brain methionine deficiency in methionine synthase deficiency has been related to disturbances in brain development, which early treatment in neonates and infants can reverse [86, 87]. Thus, treatment with methionine substitution could be considered a therapeutic target to be initiated in early infancy.

10‐Formyltetrahydrofolate is oxidized to carbon dioxide via 10‐formyl‐tetrahydrofolate dehydrogenase (Aldh1l1 and Aldh1l2) while reducing NADP to NADPH [44, 45], contributing substantially to the reduced NADPH pool in mitochondria [54]. In mouse brain, both enzymes are abudantly expressed, Aldh1l1 more than Aldh1l2, whereas the common alternative source of intramitochondrial NADPH through the isocitrate‐α‐ketoglutarate shuttle shows low brain expression of the key isocitrate transporter *Slc25a1* [88]. Direct measurement of NADPH in whole cortex homogenate only identified a trend to decrease, but low saccharopine provided indirect evidence of possible NADPH insufficiency.

Next to glycine [89], brain tissue has two alternative donors for one‐carbon charging of folate: primarily serine [90, 91] and formate [39, 44]. Formate was deficient in plasma. Prenatal supplementation with formate has prevented or reduced neural tube defects and hydrocephalus in mouse models of NKH [17, 18, 19]. L‐serine can compensate to provide one‐carbons to the folate system through the serine hydroxymethyltransferase system, mainly via its mitochondrial enzyme (*Shmt2*). Indeed, L‐serine was reduced in areas where methionine was most decreased, the cortex of young animals. L‐serine was also decreased in human CSF samples [15]. L‐serine is the starting point for the synthesis of sphingosine, sphingomyelin and ceramides, and indeed, these classes of metabolites were decreased. These metabolites are crucial in the formation and stability of myelin [52, 92]. Lipids typically found in myelin membranes such as sphingomyelins specifically of long chain length and galactoceramides were reduced. The lipidome was modified towards longer chain species as is typically seen in myelin [58]. However, these were primarily present in neutral lipid species as typically associated with lipid droplets, and the polyunsaturated lipids are often associated with neural membranes, rather than being incorporated into the myelin membranes. Spongiosis with unraveling of the myelin sheet is a typical early pathological finding in NKH [93, 94, 95] spreading during infancy over the entire cortex [96]. We hypothesize that this pathologically well recognized myelin disturbance may fundamentally be related to the consequences of serine deficiency on the lipidome, which makes restoring serine a new key therapeutic target. Despite these metabolite changes, there were no changes in the abundances of the pertinent enzymes serine hydroxymethyltransferases (Shmt1, *Shmt2*) or of 10‐formyltetrahydrofolate synthetase (*Mthfd1L*) in brain tissue, thus these changes likely result from altered metabolite fluxes, which may be amenable to metabolite based approaches. Finally, the lipidome also identified decreased lyso‐phosphatidylcholines and lyso‐phosphoethanolamines. The generation of these lipids requires the action of the Patatin‐like Phospholipase Domain containing Protein A (PNPLA) class of lipases, which require interaction with lipid droplets, which may have been changed in MUT mice [97]. Deficiency of lysophosphatidic acid and associated lipids in dysfunction of PNPLA lipases has been linked to severe neurodevelopmental conditions [98, 99].

We found no evidence for other previously suggested pathophysiological mechanisms. Lipid oxidative stress was identified after glycine administration into the rat brain as elevated TBARS and reduced glutathione levels [20, 21]. In this NKH mouse model, we did not identify changes in TBARS or in glutathione levels, which were only mildly decreased in cerebellum, and there was no activation of Nrf2, a typical oxidative stress response, providing no evidence for oxidative stress. Multiple biochemical changes could affect mitochondrial bioenergetics and the respiratory chain. In mitochondrial protein synthesis, 10‐formylmethionine is essential for the formation of formylmethionine‐tRNA^Met^ which initiates protein synthesis [100], and 5,10‐methylenetetrahydrofolate is essential for base modification of mitochondrial tRNA^Leu(UUR)^ and tRNA^Lys^, without which mitochondrial translation is impaired [101]. Impairment of mitochondrial translation would affect the respiratory chain complex abundances, but no deficiencies in respiratory chain complex abundances or of respiratory chain enzyme activities were found (Table S7 and Figure S11). This does not exclude that bioenergetic deficiencies could exist in a single cell type such as the astrocyte, although it appears unlikely.

Challenging the mouse model with glycine, which was associated with a worse growth defect [22], increased glycine levels, worsened the D‐serine decrease, and perturbed the one‐carbon flow in the liver, doubling the homocysteine level and decreasing the methionine level. In plasma, homocysteine levels increased massively, but not in cortex and only mildly so in other brain regions, illustrating a tissue‐specific effect limited to liver and plasma. Older mice already have mildly elevated plasma homocysteine levels. It is unclear to what extent this relates to the human disease, which shows only mildly elevated CSF homocysteine but not of plasma homocysteine [102].

Some surprising metabolic signatures were found. In the transsulfuration pathway, cystathionine was significantly increased in brain tissue while homocysteine did not increase, and cysteine did not decrease. This thus reflects decreased activity of cystathionine γ‐lyase (*Cgl*), although the protein amount of this enzyme was not reduced on proteomics, and its cofactor pyridoxal‐5′‐phosphate was unchanged, and homocysteine, which can reduce cystathionine γ‐lyase activity [103] was unchanged. While the reason for this decreased activity is currently unclear, we hypothesize interference by accumulating toxic metabolites. Propargylglycine is a known inhibitor and raises cystathionine in brain regions, and an as yet unrecognized glycine derivative could be responsible [104]. Cystathionine has not been reviewed in human NKH disease. The known hepatic cytoprotective effects of cystathionine may contribute to the lack of liver involvement in NKH [105]. Finally, the metabolomics study identified signatures of unidentified compounds that are substantially increased in the brain. When identified, they could provide significant biomarkers of NKH or help elucidate pathophysiological mechanisms.

Limitations of this study include the evaluation of a single *Gldc* mutation with residual activity, which should be replicated in a more severe *Gldc* variant and in *Amt* variants. Evaluation of core metabolites will need to be replicated in human NKH brain, but the reports of increased brain glycine levels, decreased CSF L‐serine and D‐serine, and increased CSF homocysteine are encouragingly similar [13, 15, 102].

In summary, these studies illustrate that the biochemistry of nonketotic hyperglycinemia is complex but can be brought back to the consequences of both an increase in glycine and a deficiency of one‐carbon charging of folates, L‐serine deficiency contributing to disturbed lipid metabolism important for myelin, and deficiency of neurophysiologically important D‐serine. The evaluation of any treatment intervention should take all four elements into account, not solely glycine levels. The clinical correlates of these biochemical changes still need to be experimentally validated. Current findings suggest a relation of deficient folate one‐carbon charging to growth failure, and to brain development, consistent with rescue of fetal brain malformations in GLDC‐deficient mice by formate or methionine supplementation [17, 18]. The excess glycine likely relates to seizure propensity and encephalopathic effects as previously documented in human patients [5, 6]. The young J129 mouse best reflected the human biochemistry in key elements such as serine metabolism. The strain differences point to the presence of modifier elements to the biochemical disturbance with the potential to influence clinical outcome in the human disease. This more complete biochemical picture of NKH will allow effective evaluation of newly developed treatments.

## Author Contributions

Study concept and design: J.L.K.V.H., M.A.S. Animal study data: M.A.S., H.J., K.N.M., L.D.K. Biochemical and genetic studies: M.A.S., L.D.K., R.M., T.W., R.B., L.A.‐L., U.C., K.L., N.D.E.G., E.A., R.A.V.H., M.W.F., L.D.K., N.D.E.G. Proteomics studies: C.R.M., R.R., D.H.H., D.A.S., M.W.F. Metabolomics studies: F.W., S.L., B.M., M.S., R.R., D.A.S. Statistical analysis: J.L.K.V.H., X.L. First draft writing: J.L.K.V.H., M.A.S. Critical rewriting: M.A.S., J.L.K.V.H., N.D.E.G., K.N.M., E.A., U.C., T.W., R.R., K.A.D., N.D.E.G. Final responsibility, guarantor, and communicating author: J.L.K.V.H.

## Funding

Funding for this study was received from the NKH Crusaders, Brodyn's Friends, Nora Jane Almany Foundation, the Dickens Family Foundation, the Lucas John Foundation, Les Petits Bourdons, Joseph's Goal, the Barnett Family, Maud & Vic Foundation, Madi's Mission NKH fund, from Dr. and Ms. Shaw, and the University of Colorado Foundation NKH research fund to J.L.K.V.H., N.D.E.G., and K.L. were funded by the MRC (W00500X) and the Biological Mass Spectrometry Centre at UCL GOS Institute of Child Health. The work performed in the lab of R.R. is supported by NIH/NCCR 1S10 OD028538‐01A1 to Nichole Reisdorph. K.N.M. gratefully acknowledges financial support from the William R. Hummel Homocystinuria Research Fund and holds the Ehst‐Hummel‐Kaufmann Family Endowed Chair in Inherited Metabolic Disease. All funding sources had no role in the design or execution of the study, the interpretation of data, or the writing of the study.

## Ethics Statement

Mouse studies were carried out with approval from the Institutional Animal Care and Use Committee of the University of Colorado Anschutz Medical Campus (IACUC# 00413 and 1520).

## Conflicts of Interest

The University of Colorado (J.L.K.V.H., M.A.S., K.N.M., H.J.) has filed intellectual property protection for certain biochemical treatments of NKH. K.L. and N.D.E.G. are named on patents filed by UCL Business regarding treatment for nonketotic hyperglycinemia. Otherwise, the authors declare that they had no interests that might be perceived as posing a conflict or bias to this subject matter.

## Supporting information


**Data S1:** Supporting Information.

## Data Availability

The data that support the findings of this study are available from the corresponding author upon reasonable request.
