# Supplementary material for: A Nonketotic Hyperglycinemia Mouse Shows Wide‐Ranging Biochemical Consequences of Elevated Glycine, Reduced Folate One‐Carbon Charging, and Serine Deficiency
Source: J Inherit Metab Dis. 2026 Jan 28;49(1):e70137. doi: 10.1002/jimd.70137 (PMC12848846; doi:10.1002/jimd.70137)
Supplement: Supplementary file 1 — Data S1: Supporting Information. [file JIMD-49-0-s001.pdf]

Supplemental Materials

A nonketotic hyperglycinemia mouse shows wide-ranging biochemical consequences of elevated glycine, reduced folate one-carbon charging and serine deficiency

Michael A. Swanson[1], Hua Jiang[1], Lakshmi Divya Kolora[1], Rachel Molino[1], Richard Reisdorph[2], Cole R. Michel[2], Katrina A. Doenges[2], Kit-Yi Leung[3], Xiangping Lin[4], Frank Wong[4,5], Samual Lancaster[4], Basil Michael[4], Michael Snyder[4,5], Daniella H. Hock[6,7,8], David A. Stroud[6,7,8], Tim Wood[1,9], Robert Binard[9], Laura Anderson-Lehman[9], Uwe Christians[10], Erland Arning[11], Marisa W. Friederich[1,9], Roxanne A. Van Hove[1], Kenneth N. MacLean[1], Nicholas D.E. Greene[3], Johan L.K. Van Hove[1,9]

Supplemental Methods:

Formate analysis: Formate was measured in plasma or brain homogenates by addition of 2:1 (v:v) $^{13}C,^{2}H$-formate (M+2) (Sigma-Aldrich) (1 mM) internal standard solution in water, and derivatized with benzyl-alcohol (Sigma-Aldrich) and methyl chloroformate (Sigma-Aldrich) as detailed in Meiser et al.[26] After extraction in hexane, derivatized samples were analyzed using a Schimadzu GC system (2010 Plus) with a Phenomenex DB-5MS column (30 m x 0.25 mm), coupled to a TQ 8050 triple quadrupole MS system. Samples of 1 µL were injected into the GC-MS/MS in split mode (1:10, 18.3 mL/min) and helium gas flow was held constant at 1.48 ml/min. The oven was held at 60ºC for 1 min, then increased linearly by 30ºC/min to 230ºC followed by a 1 min hold for a run time of 7.67 min. The inlet temperature was 280ºC, the interface temperature was 230ºC, and the quadrupole temperature was 200º C; with electron ionization voltage set at 60 eV. The mass spectrometer was operated in multiple reaction monitoring mode with transitions 136.05→108.05 and 138.05 →108.05 for formate and internal standard respectively. Recorded data were processed using the LabSolution software (Shimadzu).

Creatine and guanidinoacetate: Creatine and guanidinoacetate were measured as previously described with minor modifications.[28,29] Briefly, 10 µL of lysate was added to 395 µL methanol containing d3-creatine (Cambridge Isotope Labs) and d2-guanidinoacetate (CDN Isotopes). The samples were centrifuged at 13000 rpm for 3 minutes. 306 µL resulting supernatant was removed and dried under nitrogen. Then, 50 µL 3.0 N HCl in n-butanol (Regis Technologies, Inc.) was added and the sample was incubated for 15 minutes at 65° C. The sample was dried under nitrogen, and resuspended in 200 µL 50% acetonitrile with 0.1% formic acid for analysis. Analyses were performed using an Agilent 6470 LC/QQQ system with a Poroshell 120 SB18 column (1.9 µm, 2.1x150 mm). Buffer A was water with 0.1% formic acid, and buffer B was acetonitrile with 0.1% formic acid, with a gradient starting with 99% buffer A going to 95% buffer B linearly over 5 minutes, followed by a 4 minute gradient wash to 99% buffer A. Retention times for guanidinoacetate and creatine and the corresponding stable isotopes were 3.2 minutes and 3.4 minutes, respectively. Multiple reaction monitoring transitions were creatine (188.1>90), d3-creatine (191.1>93), guanidinoacetate (174>101) and d3-guanidinoacetate (176>103). Quantification was performed using standard curves for each analyte. Unfortunately, insufficient plasma remained available for analysis in this tissue.

Metabolomics and lipidomic profiling:

For tissue extraction, 40 mg sections of frozen mouse brain tissue were homogenized in 500 µl of cold methanol by bead beating (MP Biosciences cat# 6913-100, Solon, OH) at 4°C (3 x 45s). Metabolites and lipids were extracted using a liquid-liquid biphasic separation with cold methyl tert-butyl ether (MTBE), methanol, and water. To begin, 1 mL MTBE was added to 300 µL of homogenate and spiked with 40 µL of deuterated lipid internal standards (Sciex, cat# 5040156, lot# LPISTDKIT-103). The samples were sonicated (3 x 30s) and agitated at 4°C for 30 minutes. After the addition of 250 µL cold water, samples were vortexed for 1 minute then centrifuged at

14,000 g for 5 minutes at 4º C. The upper organic phase contains the lipids while the lower aqueous phase contains metabolites and proteins were precipitated at the bottom of the tube.

1) Metabolites: To further precipitate proteins, 500 µL 1:1:1 acetone: acetonitrile: methanol spiked with 16 labeled metabolite internal standards was added to 300 µL of the aqueous phase and 200 µL of the organic phase and incubated overnight at -20º C. After centrifugation at 14,000 g for 10 min at 4°C, the metabolic extracts were dried down to completion under a stream of nitrogen gas and resuspended in 100 µl 50:50 methanol/water (v:v) for LC-MS.
2) Complex lipids: 700 µl of the organic phase was dried down under a stream of nitrogen and resolubilized in 200 µl of methanol for storage at -20° C until analysis. The day of the analysis, samples were dried down, resuspended in 300 µl of 10 mM ammonium acetate in 90:10 methanol/toluene (v:v), and centrifuged at 14,000 g for 5 min at 4° C.

Data acquisition

Metabolite extracts were analyzed using a broad-spectrum untargeted LC-MS platform[33] while complex lipids were quantified using a targeted MS-based approach.[34]

1) Untargeted Metabolomics by Liquid Chromatography (LC)-MS. Metabolic extracts were analyzed four times using HILIC and RPLC separation in both positive and negative ionization modes. Data were acquired on a Thermo Q Exactive HF mass spectrometer for HILIC (Thermo Fisher Scientific, Bremen, Germany) and a Thermo Q Exactive mass spectrometer for RPLC (Thermo Fisher Scientific, Bremen, Germany). Both instruments were equipped with a HESI-II probe and operated in full MS scan mode. MS/MS data were acquired on quality control samples (QC) consisting of an equimolar mixture of all samples in the study. HILIC experiments were performed using a ZIC-HILIC column 2.1 x 100 mm, 3.5 µm, 200Å (Merck Millipore, Darmstadt, Germany) and mobile phase solvents consisting of 10 mM ammonium acetate in 50/50 acetonitrile/water (A) and 10 mM ammonium acetate in 95/5 acetonitrile/water (B). RPLC

experiments were performed using a Zorbax SBaq column 2.1 x 50 mm, 1.7 μm, 100Å (Agilent Technologies, Palo Alto, CA) and mobile phase solvents consisting of 0.06% acetic acid in water (A) and 0.06% acetic acid in methanol (B). Data quality was ensured by (i) injecting 6 and 12 pool samples to equilibrate the LC-MS system prior to running the sequence for RPLC and HILIC, respectively, (ii) injecting a pooled sample every 10 injections to control for signal deviation with time, and (iii) checking mass accuracy, retention time and peak shape of internal standards in each sample.

2) Targeted Lipidomics using the Lipidyzer Platform. Lipid extracts were analyzed using the Lipidyzer platform that comprises a 5500 QTRAP system equipped with a SelexION differential mobility spectrometry (DMS) interface (Sciex) and a high flow LC-30AD solvent delivery unit (Shimadzu, Columbia, MD). Briefly, lipid molecular species were identified and quantified using multiple reaction monitoring (MRM) and positive/negative ionization switching. Two acquisition methods were employed covering 13 lipid classes; method 1 had SelexION voltages turned on while method 2 had SelexION voltages turned off. Data quality was ensured by i) tuning the DMS compensation voltages using a set of lipid standards (cat# 5040141, Sciex) after each cleaning, more than 24 hours of idling or 3 days of consecutive use, ii) performing a quick system suitability test (QSST) (cat# 5040407, Sciex) before each batch to ensure acceptable limit of detection for each lipid class, and iii) triplicate injection of lipids extracted from a reference plasma sample at the beginning of the batch.

Data Processing

Metabolomics: Data from each mode were independently analyzed using Progenesis QI software (v2.3) (Nonlinear Dynamics, Durham, NC). Metabolic features from blanks and those that didn't show sufficient linearity upon dilution in QC samples (r<0.6) were discarded. Only metabolic features present in >2/3 of the samples were kept for further analysis. Missing values

were imputed by drawing from a random distribution of low values in the corresponding sample. Intensity drift was corrected using LOESS (locally estimated scatterplot smoothing)[33]. Data quality post-normalization was verified by ensuring clustering of pooled sample replicates on a principal component analysis plot (Supplementary Figure?). Data from each mode were merged metabolic features were annotated as follows. Peak annotation was first performed by matching experimental m/z, retention time, and MS/MS spectra to an in-house library of analytical-grade standards. Remaining peaks were identified by matching experimental m/z and fragmentation spectra to publicly available databases including HMDB, MoNA, and MassBank using the R package ‘metID’ (v0.2.0). We used the Metabolomics Standards Initiative (MSI) level of confidence to grade metabolite annotation confidence (level 1 - level 3). Level 1 represents formal identifications where the biological signal matches accurate mass, retention time, and fragmentation spectra of an authentic standard run on the same platform. For level 2 identification, the biological signal matches accurate mass and fragmentation spectra available in one of the public databases listed above. Level 3 represents putative identifications that are the most likely name based on previous knowledge.

Targeted Lipidomics: Lipidyzer data were reported by the Lipidomics Workflow Manager (LWM, v1.0.5.0) software which calculates concentrations for each detected lipid as average intensity of the analyte MRM/average intensity of the most structurally similar internal standard (IS) MRM multiplied by its concentration. Lipids detected in less than 2/3 of the samples were discarded and missing values were imputed by drawing from a random distribution of low values class-wise in the corresponding sample. Data quality was verified by ensuring clustering of the quality control replicates analyzed on a principal component analysis plot. We detected lipid species belonging to 13 classes (e.g. CE, CER, DAG, FFA, HCER, LCER, DCER, LPE, LPC, PC, PE, SM, TAG) and their abundance was reported as concentrations in nmol/g.

Lipidomics data were filtered using a low-variance filter with a relative standard deviation (RSD) cutoff set of 10%, and a low-abundance filter with a median intensity threshold of 20%. The filtered data were normalized by the median values, log2 transformed, and Pareto-scaled (mean centered and divided by the square root of the standard deviation of each variable). The resulting dataset was then subjected to principal component analysis (PCA), and a PCA score plot was generated using MetaboAnalyst 6.0[43]. For lipidome metabolic pathways, including lipid networks and fatty acid networks analysis, BioPAN was used (https://www.lipidmaps.org/biopan/)[44]. For lipid ontology enrichment, LION was employed[45]. Visualizations were generated in R 9version 4.5.2, 2025-10-31) using ggplot2. From the resulting dataset.

Glycerophosphoserines analysis:

Sample preparation. A modified methyl tert-butyl ether (MTBE) liquid-liquid extraction method was used to separate hydrophobic and hydrophilic fractions as previously described[35]. Briefly, brain samples were bead homogenized in PBS at 100 mg/mL and 100 µL of homogenate was transferred to a new microfuge tube. An internal standard mix was spiked into each sample (Avanti Polar Lipids, Alabaster, AL) for quality control. Then, 400 µL ice-cold methanol was added, samples were vortexed and spun at 18,000 x g, 0°C for 15 minutes. The supernatant was transferred to glass culture tubes and dried under nitrogen at 35°C. A volume of 2.5 mL of MTBE was added to the dried residue and vortexed, then 750 µL water was added and the mixture was vortexed and centrifuged at 1,000 x g for 10 min. The upper MTBE layer was transferred to a new glass culture tube and the lower aqueous layer was re-extracted with 3.0 mL MTBE as above. The second MTBE extraction layer was combined with that of the first. The combined MTBE layers were dried under nitrogen at 35°C and the dried residue was resuspended in 200 µL methanol for analysis.

LC-MS data acquisition. Samples were analyzed by liquid chromatography mass spectrometry (LC-MS) using an Agilent 6545 quadrupole time-of-flight mass spectrometer (QTOF, Agilent Technologies, Santa Clara, CA, USA) as previously described[35]. Samples were injected onto an Agilent Zorbax SB-C18 Rapid Resolution High Definition, 1.8 µm (2.1 mm × 100 mm) analytical column attached to an Agilent 1290 series LC pump. LC flow rate was 700 µL/min with a maximum pressure of 1000 bar. The mobile phases for separation consisted of A) water with 0.1 % formic acid and B) isopropyl alcohol/acetonitrile/water (60:36:4) with 0.1 % formic acid as the aqueous and lipid phases, respectively. Initial composition was 30 % B then increased linearly using the following gradient: 0.00–1.00 min to 70 % B, 1.00–7.92 min to 100 % B, then held at 100 % B from 7.92 to 10.40 min. Initial conditions were resumed at 10.5 minutes and held until 15.1 min for column equilibration.

Analysis was performed using electrospray ionization (ESI) in positive ion mode. MS parameters were as follows: scan rate of 2 spectra/s, mass range 75–1700 *m/z*, drying gas temperature of 300°C with flow rate of 12 L/min, nebulizer pressure 35 psi, sheath gas temperature of 275°C with flow rate of 12 L/min, skimmer voltage 65 V, capillary voltage 3.5 kV, nozzle voltage 250 V, and Fragmentor voltage 100 V. A reference mix with masses of 121.0509 and 922.0098 was used for external calibration (Agilent Technologies, Santa Clara, CA, USA).

Western blot analysis

Individual protein expression levels were assessed by sodium dodecyl sulfate-polyacrylamide gel electrophoresis (SDS-PAGE) using a 4–20% gradient gel, followed by Western blotting. Proteins were transferred onto a polyvinylidene difluoride (PVDF) membrane (Immobilon-P) and blocked with 5% Bovine Serum Albumin(BSA) in Tris-buffered saline with 0.1% Tween-20 (TBS-T) for 1 hour at room temperature to minimize non-specific binding. Membranes were then incubated overnight at 4°C with primary antibodies specific to the target proteins as listed in the table below. After thorough washing in TBS-T, membranes were incubated with appropriate

horseradish peroxidase (HRP)-conjugated secondary antibodies for 1 hour at room temperature. Detection was performed using the ECL Prime Western Blotting Detection Reagent (Amersham,Cytiva) according to the manufacturer's instructions. Chemiluminescent signals were visualized using a digital imaging system (ChemiDoc MP, Bio-Rad), and band intensities were quantified using ImageJ software (NIH), normalized to housekeeping protein. The specifics of the antibodies used are listed in the table.

Target protein	Manufacturer	Catalog#	Type	Dilution
BCL9	ProteinTech	22947-1-AP	Polyclonal rabbit IgG	1:1,000
DPY30	ProteinTech	16281-1-AP	Polyclonal rabbit IgG	1:1,000
FLOR1	ProteinTech	29472-1-AP	Polyclonal rabbit IgG	1:5,000
HDAC1	Cell Signaling	5356S	Monoclonal mouse IgG	1:5,000
TDH	Invitrogen	PA5-90554	Polyclonal rabbit IgG	1:3,000
SLC6A20	Invitrogen	PA5-68332	Polyclonal rabbit IgG	1:1000
GAST3	Invitrogen	PA5-121943	Polyclonal rabbit IgG	1:3,000
GAPDH	Proteintech	60004-1-1g	Monoclonal mouse IgG	1:5000
RUSC2	Novus Biolog.	NBP2-81786	Polyclonal rabbit IgG	1:1,000
Tubulin	Cell signaling	2148S	Polyclonal Rabbit IgG	1:5000
Citrate synthase	Abcam	Ab129095	Polyclonal rabbit IgG	1:2000

Supplemental Tables

Supplemental Table 1. Sex differences in core metabolites in 5-week-old J129 wild type and mutant mice with nonketotic hyperglycinemia

Analyte	Mean±SD	Median (IQR)	Mean±SD	Median (IQR)	p-value t-test or MWU	Benjamini-Hochberg p-value	% change
**WT J129 5 wk old**	**MALE N=9**		**FEMALE N=7**				
**PLASMA** analytes in µmol/L							
Glycine	325±29*	324 (305-329)	344±39	330 (311-376)	0.408*	0.51	+6%
L-Serine	120±11	118 (113-132)	164±27	171 (138-178)	**0.004**	**0.025**	**+37%**
Threonine	120±17	124 (105-135)	109±32	98 (92-126)	0.400	0.51	-9%
Methionine	39.7±4.2	41.5 (37.4-42.7)	42.8±13.8	37.1 (33.1-47.8)	0.536	0.596	+8%
Glutamate	52.4±14.7	54.9 (42.2-60.0)	43.4±5.8	43.1 (39.5-45.6)	0.149	0.418	-17%
Glutamine	589±62	577 (534-633)	644±90	657 (600-697)	0.168	0.418	+9%
Homocysteine	4.16±0.98	4.24 (3.68-4.40)	5.68±0.61	5.47 (5.04-6.32)	**0.005**	**0.025**	**+37%**
Cysteine	134±19	126 (122-140)	123±23	130 (103-132)	0.358	0.51	-8%
Cysteinylglycine	4.12±0.45	4.09 (3.71-4.58)	4.06±0.22	4.07 (3.87-4.25)	0.766	0.766	-1%
Glutathione	77.8±8.5*	83.2 (71.1-84.8)	70.9±13.5*	79.5 (59.5-81.0)	0.209*	0.418	-9%
**CORTEX** analytes in nmol/g							
Glycine	840±61	829 (789-913)	875±52	871 (837-924)	0.256	0.331	+4%
L-Serine	633±53	615 (589-677)	688±30	680 (662-717)	**0.027**	0.0616	**+9%**
D-serine	290±18	293 (278-301)	317±26	312 (291-352)	**0.028**	0.0616	**+9%**
Threonine	458±79	449 (423-502)	420±34	431 (387-441)	0.271	0.331	-8%
Methionine	73.4±14.5	67.1 (60.6-88.7)	67.4±14.7	65.3 (59.1-69.6)	0.430	0.473	-8%
Glutamate #	15.51±2.25*	16.73 (13.29-16.95)	18.49±1.29	18.34 (17.43-19.19)	**<0.001***	**<0.00367**	**+19%**
Glutamine #	4.09±0.74	4.20 (3.51-4.58)	4.97±1.35	4.46 (4.25-4.88)	0.119	0.211	+22%
Homocysteine	3.52±0.77	3.65 (3.07-4.02)	4.04±0.36	4.10 (3.70-4.30)	0.134	0.211	+15%
Cysteine	110±49	100 (77-136)	113±20.0	122 (98-126)	0.905	0.905	+3%
Cysteinylglycine	2.22±0.45	2.15 (1.80-2.55)	3.33±0.38	3.20 (3.00-3.70)	**<0.001**	**<0.00367**	**+50%**
Glutathione	676±81	676 (610-717)	456±33	469 (430-478)	**<0.001**	**<0.00367**	**-32%**
**MUT J129 5 wk**	**MALE N = 7**		**FEMALE N=7**				
**PLASMA** analytes in µmol/L							
Glycine	565±117	536 (507-608)	824±117	787 (738-984)	**0.001**	**<0.0033**	**+15%**
L-Serine	104±17	97 (90-123)	154±33	171 (123-178)	**0.004**	**0.01**	**+48%**
Threonine	107±37*	83 (77-157)	95±20	94 (79-111)	0.902*	0.902	-11%
Methionine	33.8±9.9	29.1 (24.6-44.0)	31.6±5.4	32.2 (27.2-33.6)	0.617	0.804	-7%
Glutamate	44.8±9.9	45.2 (36.2-48.6)	45.7±11.2	46.0 (34.4-52.9)	0.880	0.902	+2%
Glutamine	506±23	505 (489-522)	651±44	648 (630-673)	**<0.001**	**<0.0033**	**+29%**
Homocysteine	4.14±0.80	3.88 (3.80-4.78)	5.37±2.27	5.92 (2.84-7.36)	0.201	0.402	+30%
Cysteine	127±31	117 (100-164)	138±53	127 (107-169)	0.643	0.804	+9%
Cysteinylglycine	3.87±0.41	3.73 (3.50-4.14)	2.38±0.58	2.31 (2.08-2.59)	**<0.001**	**<0.0033**	**-38%**
Glutathione	71.6±9.8	73.8 (61.6-78.7)	64.3±16.5	68.9 (59.3-74.8)	0.333	0.555	-10%
**CORTEX** analytes in nmol/g							

Glycine	1486±96	1489 (1370-1582)	1675±191*	1709 (1642-1818)	**0.026***	0.0953	+13%
L-Serine	553±44	546 (543-588)	577±81*	590 (566-613)	0.209*	0.383	+4%
D-serine	245±21	246 (229-261)	230±40*	242 (217-250)	0.456*	0.557	-6%
Threonine	404±53	413 (360-447)	417±33	431 (379-443)	0.613	0.6897	+3%
Methionine	55.6±15.2	49.9 (42.1-75.3)	51.3±16.7	53.3 (29.9-60.7)	0.627	0.6897	-8%
Glutamate #	15.92±0.67	15.88 (15.36-16.53)	16.82±2.33*	17.64 (16.82-17.88)	**0.038***	0.1045	+6%
Glutamine #	4.77±1.90*	3.93 (3.64-4.88)	4.28±0.80	4.15 (3.87-4.87)	1.00*	1.00	-10%
Homocysteine	3.36±0.52	3.30 (3.00-3.70)	3.73±0.28	3.80 (3.60-4.00)	0.119	0.262	+11%
Cysteine	120±17	123 (120-135)	126±8	124 (105-138)	0.420	0.557	+5%
Cysteinylglycine	4.20±0.72	4.20 (3.50-4.90)	3.19±0.53*	3.00 (2.90-3.40)	**0.011**	0.0605	-24%
Glutathione	601±46	586 (567-652)	486±12	484 (475-502)	**<0.001**	**<0.011**	**-19%**
**HIPPOCAMPUS** analytes in nmol/g							
Glycine	2031±112	2052 (1907-2111)	2094±205	2061 (1875-2304)	0.484	0.734	+3%
L-Serine	672±31	673 (642-699)	665±50	649 (626-700)	0.760	0.836	-1%
D-serine	235±21	239 9211-252)	229±21	223 (211-243)	0.636	0.777	-3%
Threonine	320±44	313 (289-355)	356±46	366 (332-392)	0.156	0.286	+11%
Methionine	72.3±16.6	71.4 (59.3-81.7)	90.3±20.7	90.0 (79.3-103.3)	0.098	0.216	+25%
Glutamate #	10.27±0.78	10.36 (9.99-10.77)	9.99±0.84	9.89 (9.42-10.92)	0.534	0.734	-3%
Glutamine #	5.17±2.11*	4.43 (3.97-5.28)	4.56±0.80	4.21 (3.99-4.71)	1.000*	1.00	-12%
Homocysteine	3.49±0.60*	3.70 (3.61-3.77)	2.68±0.50	2.56 92.27-3.17)	**0.017***	**0.0467**	**-23%**
Cysteine	82±17	83.4 (64.7-95.6)	114±25	115.6 (85.4-132.7)	**0.016**	**0.0467**	**+39%**
Cysteinylglycine	5.68±0.71*	5.94 (5.46-6.10)	3.35±0.41	3.34 (2.89-3.72)	**<0.001***	**<0.0055**	**-41%**
Glutathione	573±49	585 (515-611)	423±44*	406 (393-479)	**<0.001***	**<0.0055**	**-26%**
**CEREBELLUM** analytes in nmol/g							
Glycine	3029±247	3056 (2954-3203)	3406±227	3318 (3222-3550)	**0.012**	**0.020**	**+12%**
L-Serine	569±55	664 (637-724)	673±41	558 (540-605)	**0.002**	**0.010**	**+18%**
Threonine	585±91	578 (490-693)	453±90	476 (375-541)	**0.018**	**0.0257**	**-23%**
Methionine	89.0±15.0	83.5 (75.6-104.0)	70.0±9.3	71.4 (65.9-75.5)	**0.007**	**0.014**	**-21%**
Glutamate #	9.91±0.49	9.83 (9.38-10.37)	10.29±0.24	10.35 (10.13-10.52)	0.101	0.126	+4%
Glutamine #	7.17±2.26*	6.40 (5.92-7.46)	6.77±0.70	6.63 (6.10-7.05)	0.535*	0.594	-6%
Homocysteine	2.45±0.25	2.51 (2.28-2.63)	2.48±0.09	2.45 (2.40-2.57)	0.743	0.743	+1%
Cysteine	99±9	98.8 (94.2-106.7)	73±5	71.2 (69.3-79.3)	**<0.001**	**<0.010**	**-26%**
Cysteinylglycine	6.93±0.30	6.86 (6.67-7.06)	2.45±0.25	7.67 (7.28-7.90)	**0.006**	**0.014**	**-65%**
Glutathione	608±36	618 (566-641)	663±19	666 (643-682)	**0.004**	**0.013**	**+9%**
**LIVER** analytes in nmol/g							
Glycine	4113±586	4122 (3603-4600)	4926±748	5198 (4106-5592)	**0.043**	0.43	+20%
L-Serine	379±50	360 (343-432)	392±53	382 (357-427)	0.632	0.771	-3%
Threonine	216±36	227 (182-247)	174±66	158 (114-245)	0.172	0.653	-19%
Methionine	128.7±15.0	125.7 (114.4-140.4)	127.6±15.6	123.3 (114.3-142.6)	0.895	0.895	-1%
Glutamate #	1.198±0.339	1.396 (0.763-1.461)	1.205±0.404	1.263 (0.920-1.751)	0.596	0.771	+1%
Glutamine #	5.49±074	5.51 (4.79-6.16)	5.30±1.02	5.40 (5.00-6.15)	0.686	0.771	-3%
Homocysteine	5.37±0.86	5.46 (4.34-6.05)	4.88±0.70	5.22 (4.18-5.40)	0.261	0.653	-9%
Cysteine	460±32	462 (428-482)	481±48	502 (431-517)	0.354	0.708	+5%
Cysteinylglycine	14.06±2.58	14.87 (11.44-15.77)	14.53±1.67	14.62 (13.42-15.54)	0.694	0.771	+3%

Glutathione	2519y±546	2400 (2007-3144)	2159±563	2085 (1622-2788)	0.237	0.653	-14%

Legend: The comparison in core metabolites between females compared to male mice is shown for wild type mice WT or for homozygous mutant mice MUT. Values of metabolites at age 5 weeks in plasma as shown as mean and SD in µM. Values of the metabolites in forebrain cortex, hippocampus and cerebellum are shown in nmol/g tissue (# except for glutamate and glutamine shown in µmol/g tissue). There were 9 male and 7 female WT mice and 7 male and 7 female MUT mice. A significant deviation of the normal distribution is indicated by an asterisk *. Comparisons are done by Student t-test, or by Mann-Whitney-U test if any of the two populations significantly deviated from the normal distribution as indicated by an asterisk *, and p-values are shown. A Benjamini-Hochberg corrected p-value is provided for the multiple comparisons within each tissue type. The % differences between the female mice in comparison with the male mice are given with green values for significant increase and red values for a significant decrease.

Supplemental Table 2. Age differences in core metabolites comparing 5-week-old J129 mice with adult 12.9-week-old mice, wild type and mutant for nonketotic hyperglycinemia

	Young 5-week-old mice		Mature 12.9-week-old mice		Comparison		Change
Analyte	AVG±SD	Median (IQR)	AVG±SD	Median (IQR)	p-value Student / MWU	p-value Benjamini-Hochberg	% change
**WT MICE J129**	N = 16		N = 14				
**PLASMA**							
Glycine	334±34*	324 (311-364)	335±38	337 (303-361)	0.697*	0.697	0%
L-Serine	139±30	132 (117-168)	155±23	147 (140-169)	0.126	0.140	+11%
Threonine	115±24	109 (96-129)	167±22	166 (156-190)	**<0.001**	**<0.0025**	**+45%**
Methionine	41.1±9.4*	40.6 (36.0-43.6	55.7±12.6	54.2 (45.3-63.7)	**<0.001***	**<0.0025**	**+36%**
Glutamate	48.5±12.3	45.5 (40.7-55.4)	40.5±7.9	39.0 (33.3-46.8)	**0.045**	0.0642	-16%
Glutamine	613±78	609 (549-677)	553±71	541 (523-587)	**0.037**	0.0617	-10%
Homocysteine	4.9±1.1	4.99 (4.15-5.98)	6.1±2.0	5.98 (4.43-7.62)	0.070	0.0875	+24%
Cysteine	128±22	128 (117-135)	223±53	215 (181-273)	**<0.001**	**<0.0025**	**+74%**
Cysteinylglycine	4.1±0.3	4.08 (3.85-4.29)	1.9±0,2	1.92 (1.70-2.08)	**<0.001**	**<0.0025**	**-54%**
Glutathione	74.3±11.4	79.7 (63.3-83.9)	58.0±21.4	54.5 (50.8-71.4)	**0.019**	0.038	-22%
**CORTEX** analytes in nmol/g							
Glycine	855±58	847 (801-915)	1175±297*	1103 (1004-1186)	**<0.001***	**<0.00367**	**+37%**
L-Serine	657±52	660 (608-705)	652±98*	628 (582-680)	0.275*	0.336	0%
D-serine	302±25	299 (290-313)	283±24	283 (266-292)	**0.048**	0.0754	-6%
Methionine	70.8±14.4*	65.4 (60.4-81.5)	132.3±36.9	127.4 (97.7-167.6)	**<0.001***	**<0.00367**	**+87%**
Glutamate	16.81±2.39*	17.17 (16.50-18.16)	14.24±0.87	14.28 (13.78-14.68)	**<0.001***	**<0.00367**	**-15%**
Glutamine	4.47±1.11*	4.40 (4.11-4.61)	3.93±0.43	3.98 (3.60-4.25)	**0.025***	**0.0458**	**-12%**
Homocysteine	3.80±0.62*	3.80 (3.60-4.25)	3.23±0.48	3.32 (2.80-3.70)	**0.008***	**0.0176**	**-15%**
Cysteine	111±35	107 (87-125)	126±13	124 (120-133)	0.144	0.198	+14%
Cysteinylglycine	2.82±0.70	3.00 (2.15-3.25)	2.94±0.28	2.95 (2.67-3.20)	0.547	0.602	+4%
Glutathione	558±127	496 (456-676)	430±30	426 (408-442)	**0.004**	**0.011**	**-23%**
**HIPPOCAMPUS** analytes in nmol/g							
Glycine	928±65	913 (877-1002)	849±72	827 (801-924)	**0.004**	**0.0056**	**-9%**
L-serine	719±44	730 (696-749)	675±35	669 (652-706)	**0.006**	**0.0082**	**-6%**
D-serine	274±23*	274 (267-288)	256±14	256 (247-263)	**<0.001***	**<0.0018**	**-7%**
Methionine	83.4±10.5	84.3 (77.2-91.1)	62.0±8.0	62.2 (56.2-67.4)	**<0.001**	**<0.0018**	**-26%**
Threonine	334±76	315 (279-364)	300±37*	287 (277-321)	0.166*	0.203	-10%
Homocysteine	3.11±0.56	3.08 (2.61-3.62)	3.02±0.31	3.02 (2.76-3.30)	0.604	0.637	-3%
Cysteine	111±28	95 (90-146)	317±27	314 (302-341)	**<0.001**	**<0.0018**	**+185%**
Cysteinylglycine	4.38±1.28*	5.13 (2.96-5.57)	5.20±1.11*	5.00 (4.60-5.31)	0.637*	0.637	+19%
Glutamate #	10.13±0.27	10.05 (9.96-10.36)	12.02±0.46	11.99 (11.80-12.36)	**<0.001**	**<0.0018**	**+19%**
Glutamine #	4.62±0.48*	4.54 (4.36-4.68)	3.47±0.32	3.47 (3.17-3.65)	**<0.001**	**<0.0018**	**-25%**
Glutathione	493±89*	529 (406-576)	608±37	613 (581-636)	**<0.001***	**<0.0018**	**+23%**
**CEREBELLUM** analytes in nmol/g							
Glycine	917±86	904 (871-965)	849±99	844 (780-899)	0.053	0.059	-7%

L-serine	548±79	515 (489-625)	534±56	521 (499-583)	0.601	0.601	-3%
Methionine	85.2±5.6	84.5 (80.2-89.4)	80.9±5.1	79.6 (76.9-84.3)	**0.036**	0.045	-5%
Threonine	381±60	396 (350-421)	452±49*	444 (419-485)	**0.002***	**0.0029**	**+19%**
Homocysteine	2.53±0.26	2.55 (2.46-2.71)	1.66±0.28	1.59 (1.54-1.77)	**<0.001**	**<0.0017**	**-34%**
Cysteine	84±23*	90 (82-95)	333±46	325 (296-353)	**<0.001***	**<0.0017**	**+296%**
Cysteinylglycine	6.99±0.35	6.98 (6.73-7.10)	4.93±0.57	4.88 (4.42-5.19)	**<0.001**	**<0.0017**	**-29%**
Glutamate #	9.97±0.48	10.01 (9.60-10.36)	12.63±0.37	12.73 (12.34-12.88)	**<0.001**	**<0.0017**	**+27%**
Glutamine #	6.51±0.71*	6.51 (6.08-6.74)	5.17±0.53	5.24 (4.71-5.48)	**<0.001***	**<0.0017**	**-21%**
Glutathione	697±29	692 (674-726)	574±75	559 (528-617)	**<0.001**	**<0.0017**	**-18%**
**MUT MICE**		N = 14		N = 12			
**PLASMA**							
Glycine	695±175	722 (533-805)	1016±88	1007 (948-1094)	**<0.001**	**<0.00167**	**+46%**
L-Serine	129±37	123 (95-171)	163±40*	149 (140-174)	**0.031***	**0.0443**	**+26%**
Threonine	101±30*	88 (78-119)	186±40	178 (156-206)	**<0.001***	**<0.00167**	**+84%**
Methionine	32.7±7.7	30.6 (26.0-41.6)	67.7±18.4*	57.6 (55.0-83.9)	**<0.001***	**<0.00167**	**+107%**
Glutamate	45.2±10.2	45.2 (35.7-50.5)	41.1±11.0	37.4 (36.7-45.7)	0.326	0.326	-9%
Glutamine	578±83	557 (502-653)	535±50	523 (495-565)	0.114	0.1267	-7%
Homocysteine	4.76±1.75	4.39 (3.62-6.12)	9.72±3.48	9.57 (7.43-12.33)	**<0.001**	**<0.00167**	**+104%**
Cysteine	133±42	123 (106-165)	224±46	224 (180-273)	**<0.001**	**<0.00167**	**+68%**
Cysteinylglycine	3.13±0.91	3.47 (2.26-3.83)	1.82±0.32	1.84 (1.62-1.99)	**<0.001**	**<0.00167**	**-42%**
Glutathione	67.9±13.6	69.8 (59.9-77.7)	54.6±19.3	58.6 (46.8-67.5)	**0.049**	0.0612	-20%
**CORTEX** analytes in nmol/g							
Glycine	1581±175	1583 (1458-1733)	1704±239	1708 (1450-1891)	0.144	0.226	+8%
L-Serine	565±64	575 (546-598)	595±66	587 (549-631)	0.252	0.308	+5%
D-serine	238±32*	244 (226-258)	249±28	242 (226-266)	0.860*	0.860	+5%
Methionine	53.4±16.5	53.2 (41.4-64.0)	126.9±56.9*	123.7 (72.7-181.2)	**<0.001***	**0.00275**	**+138%**
Glutamate	16.37±1.71	16.67 (15.55-17.66)	13.82±1.06	13.89 (13.24-14.24)	**<0.001**	**0.00275**	**-16%**
Glutamine	4.53±1.43*	4.04 (3.81-4.87)	3.31±0.89*	3.44 (3.27-3.61)	**<0.001***	**0.033**	**-27%**
Homocysteine	3.54±0.44	3.65 (3.20-3.80)	3.04±0.52	3.06 (2.67-3.49)	**0.014**	**0.02567**	**-14%**
Cysteine	123±13	123 (117-136)	112±24	112 (94-131)	0.181	0.249	-9%
Cysteinylglycine	3.69±0.80	3.45 (2.97-4.30)	2.83±0.44	2.76 (2.48-3.22)	**0.002**	**0.0044**	**-23%**
Glutathione	544±67*	519 (484-596)	408±38	413 (385-438)	**<0.001***	**0.00275**	**-25%**
**HIPPOCAMPUS** analytes in nmol/g							
Glycine	2063±162	2057 (1899-2180)	1827±187	1803 (1771-1985	**0.002**	**0.0055**	**-11%**
L-serine	669±40	668 (633-699)	655±40	649 (630-687)	0.387	0.4257	-2%
D-serine	232±20	227 (211-248)	227±12	228 (219-236)	0.472	0.472	-2%
Methionine	81.3±20.3	80.5 (62.6-101.3)	63.1±6.0	61.9 (58.4-68.8)	**0.006**	**0.011**	**-22%**
Threonine	338±47	350 (297-371)	305±16	310 (293-314)	**0.024**	**0.0377**	**-10%**
Homocysteine	3.08±0.67*	3.23 (2.33-3.71)	2.99±0.16	2.98 (2.83-3.12)	0.347*	0.4257	-3%
Cysteine	98±27	93 (79-119)	328±43	317 (275-365)	**<0.001**	**<0.0037**	**+235%**
Cysteinylglycine	4.52±1.33	4.01 (3.30-5.96)	5.04±1.27*	4.74 (4.44-5.01)	0.374*	0.4257	+12%
Glutamate #	10.13±0.79	10.29 (9.41-10.81)	11.51±0.48	11.45 (11.20-11.82)	**<0.001**	**<0.0037**	**+14%**
Glutamine #	4.86±1.57*	4.32 (3.99-5.07)	3.18±0.17	3.19 (3.05-3.34)	**<0.001**	**<0.0037**	**-35%**
Glutathione	498±89	492 (403-591)	585±13	587 (579-594)	**0.003**	**0.0066**	**+17%**

**Cerebellum** analytes in nmol/g							
Glycine	3217±301	3212 (3040-3370)	2789±923*	2547 (2373.-2789)	**<0.001***	**<0.0014**	**-13%**
L-serine	621±71	632 (555-672)	603±69	603 (570-656)	0.526	0.584	-3%
Methionine	79.5±15.5	75.6 (70.1-86.6)	78.6±8.0	80.1 (73.9-84.4)	0.854	0.854	-1%
Threonine	519±111	526 (444-587)	609±98	628 (577-673)	**0.040**	**0.05**	**+17%**
Homocysteine	2.46±0.18	2.48 (2.36-2.58)	1.91±0.22	1.96 (1.71-2.06)	**<0.001**	**<0.0014**	**-22%**
Cysteine	86±15	81 (71-99)	351±46	352 (308-391)	**<0.001**	**<0.0014**	**+308%**
Cysteinylglycine	7.26±0.49	7.17 (6.82-7.67)	5.30±0.69	5.55 (4.87-5.72)	**<0.001**	**<0.0014**	**-27%**
Glutamate #	10.10±0.42*	10.26 (9.78-10.45)	12.26±1.13	12.29 (11.33-13.27)	**<0.001***	**<0.0014**	**+21%**
Glutamine #	6.97±1.62*	6.50 (6.08-7.16)	4.69±0.54	4.66 (4.40-5.01)	**<0.001***	**<0.0014**	**-33%**
Glutathione	636±40	642 (617-688)	546±71	533 (505-587)	**<0.001**	**<0.0014**	**-14%**

Legend: Values of metabolites are compared between young mice 5-weeks-old were compared to adults age-12.9-weeks for wild type (WT) mice and for homozygous mutant (MUT) mice. The distribution in each category is provided with mean (AVG) and standard deviation (SD) given, and as the median and interquartile range (IQR). Comparison is done either as by the Student t-test or by the Mann-Whitney-U test (MWU) if not normally distributed as indicated by an asterisk *. A Benjamini-Hochberg corrected p-value is provided for the multiple comparisons within each tissue type. Significant p-values are highlighted in bold. The percentage differences between the old mice in comparison with the young mice is given with green values for significant increase and red values for a significant decrease.

Supplemental Table 3: Folate species in the cortex of B6 mice corrected for protein

**Metabolite**	**WT mice**		**MUT mice**		
**Folate vitamer in nmol/mg protein**	**Mean±SD**	**Median (IQR) in %**	**Mean±SD in %**	**Median (IQR) in %**	**p-value** Student / MWU*
**B6 5 wk old**	**N = 8**		**N = 8**		
Dihydrofolate	0.0079±0.0056	0.0056 (0.0043-0.0101)	0.0053±0.0027	0.0043 (0.0039-0.0065)	0.267
Tetrahydrofolate	0.244±0.079	0.233 (0.169-0.311)	0.246±0.043	0.260 (0.196-0.284)	0.960
5-methyl-THF	0.408±0.129	0.378 (0.298-0.532)	0.304±0.071	0.301 (0.245-0.347)	0.065
5,10-methylene-THF	0.011±0.005	0.0102 (0.0081-0.0128)	0.010±0.003	0.0102 (0.0088-0.0129)	0.697
5,10-methenyl-THF	0.0013±0.0003	0.0012 (0.0005-0.0020)	0.0025±0.0016*	0.0016 (0.0014-0.0039)	0.105*
10-formyl-THF	0.0120±0.0041*	0.0109(0.0094-0.0148)	0.0124±0.0054	0.0137 (0.0072-0.0155)	0.798*
Total folates	0.684±0.191	0.651 (0.558-0.774)	0.580±0.111	0.558 (0.489-0.659)	0.203
5-methyl-THF/THF	1.774±0.753*	1.558 (1.328-1.754)	1.246±0.234	1.228 (1.098-1.483)	**0.021***

Legend: The concentration of the folate vitamers is expressed as nmol/mg protein. The ratio of 5-methyl-THF over THF is also provided.
Abbreviation: THF = tetrahydrofolate. * = not normally distributed and statistic by the Mann-Whitney-U test (MWU).

Supplemental Table 4. Other amino acid metabolites comparing mutant mice to wild type mice

J129 5 wk old mice	WT mice N=14		MUT mice N=13		Comparison		Change
**Metabolite nmol/g**	**Mean±SD**	**Median (IQR)**	**Mean±SD**	**Median (IQR)**	**p-value t-test/MWU**	**Benjamini Hochberg p**	**%change**
**CORTEX** analytes in nmol/g							
Histidine	189±37	193 (168 – 208)	183±26	181 (164-190)	0.607	0.84	-3%
Phosphoethanolamine	2,045±223	2,077 (1,900-2,207)	2,051±131	2,055 (1,982-2,132)	0.939	0.98	0%
Asparagine	156±20	157 (142 – 167)	142±13	142 (130 – 152)	0.058	0.42	-9%
Taurine	11,442±1,430	11,489 (10,752-12,204)	11,259±1,101	11,064 (10,925-11,502)	0.715	0.496	-2%
Carnosine	55.8±13.7	60.1 (49.6-63.2)	55.7±17.1	60.4 (41.1-66.7)	0.995	0.99	0%
Arginine	419±93	430 (383 – 458)	390±60	399 (345-432)	0.364	0.728	-7%
Ethenolamine	449±52	452 (418-486)	418±25	414 (398-433)	0.060	0.42	-7%
Aspartate	4,983±924	4945 (4385-5527)	4,869±482*	4668 (4524-5455)	0.650*	0.84	-2%
ß-Alanine	74.5±16.0	78.7 (61.3-86.0)	63.1±9.3	62.4 (56.7-69.1)	**0.033**	0.42	**-15%**
Threonine	439±62	437 (408-462)	410±43	417 (376-443)	0.165	0.496	-7%
Alanine	1,287±236	1297 (1055-1487)	1309±230	1338 (1124-1503)	0.809	0.91	+2%
γ-aminobutyric acid	3,773±617	3904 (3217-4201)	3,432±508	3444 (2999-3637)	0.131	0.496	-9%
α-aminoadipic acid	56.2±12.6	56.8 (47.6-67.7)	50.2±14.6	52.7 (42.1-60.0)	0.265	0.650	-11%
Proline	264±42	268 (244-283)	255±24	255 (229-275(	0.551	0.84	-3%
Ornithine	19.2±7.9	20.0 (10.6-23.8)	18.9±8.1*	14.9 (12.8-24.5)	0.943	0.98	-2%
Lysine	506±59	500 (491-539)	480±31	488 (451-504)	0.174	0.496	-5%
Tyrosine	200±45	200 (164-222)	193±37	190 (163-222)	0.661	0.84	-3%
Valine	210±36	214 (189-231)	200±25	188 (186-228)	0.415	0.775	-5%
Isoleucine	126±24	126 (118-138)	118±14	115 (105-133)	0.302	0.650	-6%
Leucine	281±53*	281 (271-300)	263±33	255 (238-301)	0.302*	0.650	-6%
Phenylalanine	178±37	184 (158-198)	170±24	160 (151-194)	0.526	0.84	-4%
Tryptophan	51.8±11.7	50.3 (43.5-59.0)	44.7±11.2	44.8 (37.4-49.4)	0.124	0.496	-14%

Legend:

Amino acid analysis in brain homogenates of young 5-week-old J129 mice comparing mutant (MUT) mice to wild type (WT) mice. The distribution in each category is provided with mean (AVG) and standard deviation (SD), and as the median and interquartile range (IQR). Comparison is done either by the Student t-test or by the Mann-Whitney-U test (MWU) if not normally distributed as indicated by an asterisk *. A Benjamini-Hochberg corrected p-value is provided for the multiple comparisons. Significant p-values are highlighted in bold.

Supplemental Table 5. Methylglyoxal adducts of 5-week-old J129 mice comparing WT and MUT mice

J129 5 wk old	WT mice N=12	MUT mice N=12	MWU-test
	**Median (IQR)**	**Median (IQR)**	**p-value**
Liver†	92.0 (84.6-124.9)	88.1 (46.6-114.5)	0.394
Cortex	18.3 (7.7-29.3)	129.3 (115.9-150.6)	<0.001
Hippocampus	49.2 (43.6-53.2)	53.8 (50.5-59.2)	0.160
Cerebellum	54.2 (50.0-78.0)	65.1 (56.2-90.4)	0.887

Legend: The comparison is made between wild type (WT) and mutant (MUT) animals for the amount of methylglyoxal protein adducts measured by ELISA and expressed as mg methylglyoxal-adduced bovine serum albumin/mg tissue. In cortex tissue, the methylglyoxal adducts were significantly increased with a trend to increase in hippocampus, but no difference was found in liver or cerebellum. † in liver only 6 mice were analyzed.

Supplemental Table 6. Acylcarnitines in cortex of 5-week-old J129 mice comparing WT and MUT mice

J129 5 wk old	WT mice	MUT mice	Student t-test	Benjamini-Hochberg corrected p-value
	N = 6	N = 6		
**Analyte**	**Mean±SD**	**Mean±SD**	**p-value**	
**C0-acylcarnitine**	3.73±0.51	2.92±0.31	**0.007**	**0.0297**
C2-acylcarnitine	0.261±0.054	0.255±0.035	0.815	0.558
C3-acylcarnitine	0.032±0.002	0.031±0.004	0.331	0.558
C4-acylcarnitine	0.014±0.002	0.0139±0.0025	0.888	0.888
**C4-hydroxyacylcarnitine**	0.0094±0.0026	0.0066±0.0011	**0.034**	0.1156
**C5 (isovaleryl)-acylcarnitine**	0.0513±0.0059	0.0370±0.008	**0.007**	**0.0297**
C5(2-methylbutyryl)-carnitine	0.0077±0.0010	0.0069±0.0014	0.301	0.558
C5:1-acylcarnitine	0.0021±0.0002	0.0021±0.0002	0.501	0.608
**C5-hydroxyacylcarnitine**	0.0595±0.0053	0.0545±0.0050	**<0.001**	**0.0085**
C6-acylcarnitine	0.0037±0.0022	0.0030±0.0004	0.495	0.608
C6-hydroxyacylcarnitine	0.0031±0.0001	0.0030±0.0001	0.073	0.207
C8-acylcarnitine	0.0054±0.0088	0.0021±0.0009	0.392	0.558
C8:1-acylcarnitine	0.0018±0.0003	0.0017±0.0002	0.394	0.558
**C8-hydroxyacylcarnitine**	0.007±0.0004	0.0059±0.0003	**<0.001**	**0.0085**
C10-hydroxyacylcarnitine	0.0015±0.0001	0.0015±0.0001	0.111	0.270
Succinylcarnitine	0.0055±0.006	0.0051±0.003	0.300	0.558
Methylmalonylcarnitine	0.0027±0.0004	0.0028±0.0002	0.803	0.866

Legend: The comparison is made between wild type (WT) and mutant (MUT) animals for the concentration of the acylcarnitines (µmol/mg tissue) measured by tandem mass spectrometry in the cortex. Results are shown as average and standard deviation and the comparison is done by Student t-test, with the Benjami-Hochberg corrected p-value for multiple comparisons shown. The concentrations of free carnitine, Isovaleryl-acylcarnitine, C4-hydroxyacylcarnitine, C5-hydroxyacylcarnitine and C8-hydroxyacylcarnitine were significantly decreased.

Supplemental Table 7. Mitochondrial respiratory chain enzyme activities in brain cortex of 5-week-old mice comparing WT and MUT mice for nonketotic hyperglycinemia

J129 5 wk old	WT mice		MUT mice		Student t-test Or MWU
	N = 7		N = 7		
	**Mean±SD**	**Median (IQR)**	**Mean±SD**	**Median (IQR)**	**p-value**
Complex I	66.6±16.5	70 (49-79)	70.5±31.7	81 (65-119)	0.778
Complex II	105.6±8.3	108 (102-114)	123.9±26.8	128 (114-140)	0.110
Complex III†	82.2±34.5	97 (55-106)	122.9±40.2	120 (86-163)	0.126
Complex II-III	207.8±81.9	209 (146-312)	217.9±35.6*	236 (221-245)	0.805*
Complex IV	68.0±22.0	59 (56-95)	82.1±17.9*	88 (74-111)	0.128*
Citrate synthase	358±63	387 (314-422)	368±111*	343 (302-546)	0.805*
**Ratios over CS**					
Complex I / CS	190±56	175 (166-216)	189±54	184 (150-237)	0.976
Complex II / CS	300±42	303 (274-338)	350±87*	385 (257-408)	0.165*
Complex III / CS	213±75	249 (174-252)	329±132	307 (217-464)	0.111
Complex II-III / CS	571±174	466 (456-766)	616±131	616 (520-728)	0.591
Complex IV / CS	189±49	190 (133-236)	230±49	226 (206-251)	0.142

Legend: In 5-week-old mice a comparison is provided between the wild type (WT) mice and the mutant (MUT) mice for the respiratory chain enzyme complex activities. The activities of complexes I, II, combined II-III, and citrate synthase (CS) are expressed as $nmol.min^{-1}.protein^{-1}$. The activities of complexes III and IV are shown as first order rate constants expressed as $nmol.min^{-1}$. Activities are also shown as ratios to the activity of citrate synthase (CS) (multiplied by 1000). The distribution in each category is provided with mean (AVG) and standard deviation (SD) given, and as the median and interquartile range (IQR). Comparison is done either as by the Student t-test or by the Mann-Whitney-U test (MWU) if not normally distributed as indicated by an asterisk *. No statistically significant differences were observed for the activities or for the ratios over citrate synthase or for the ratios over complex 2 (not shown).

Supplemental Table 8. Comparison of core metabolites in young 5-week-old J129 mice versus young 5-week-old B6 mice

5 wk old	J129X1/SvJ		C57Bl6/J		J129 vs B6	
**WT mice**	N=16		N=14			
**Analytes**	**AVG±SD**	**Median (IQR)**	**AVG±SD**	**Median (IQR)**	**p-value t-test or MWU**	**p-value Benjamini-Hochberg**
**PLASMA** analytes in µmol/L						
Glycine	333±34*	324 (311-364)	436±97*	430 (401-440)	**<0.001***	**<0.0033**
L-serine	139±30	132 (117-168)	125±17*	123 (115-131)	0.166*	0.415
Methionine	41.1±9.4*	40.6 (36.0-43.6)	43.2±9.3	44.9 (34.2-49.7)	0.294*	0.522
Homocysteine†	4.92.±1.11	4.99 (4.15-5.98)	6.14±2.90*	5.81 (3.66-7.58)	0.511	0.730
Cysteine†	128±21	128 (117-135)	190±28	184 (171-218)	**<0.001**	**<0.0033**
Cysteinylglycine†	4.09±0.34	4.08 (3.85-4.29)	2.67±0.44	2.69 (2.40-3.00)	**<0.001**	**<0.0033**
Threonine	115±24	109 (96-129)	113±21	114 (90-135)	0.801	0.890
Glutamate	48.5±12.3	45.6 (40.7-55.4)	48.6±10.2	48.0 (39.2-52.9)	0.976	0.976
Glutamine	613±78	609 (549-677)	640±66	651 (589-693)	0.313	0.522
Glutathione†	74.3±11.4*	79.7 (64.2-83.6)	78.7±14.8	78.7 (67.0-88.0)	0.734*	0.890
**CORTEX** analytes in nmol/g						
Glycine	855±58	847 (801-915)	973±165	913 (835-1065)	**0.012**	**0.019**
L-serine	657±52	660 (608-705)	569±54	557 (522-623)	**<0.001**	**<0.0018**
D-serine	302±25	299 (290-313)	241±28	247 (216-264)	**<0.001**	**<0.0018**
Methionine	70.8±14.4*	65.5 (60.4-81.5)	49.3±8.7	47.7 (40.2-57.8)	**<0.001***	**<0.0018**
Homocysteine‡	3.80±0.62*	3.80 (3.60-4.20)	2.18±0.45	2.05 (1.80-2.55)	**<0.001***	**<0.0018**
Cysteine‡	111.4±34.7	107 (87-125)	55.6±5.3	55.8 (53.7-60.2)	**<0.001**	**<0.0018**
Cysteinylglycine‡	2.82±0.70	3.00 (2.15-3.25)	2.03±0.18	2.05 (1.87-2.20)	**<0.001**	**<0.0018**
Glutamate #	16.82±2.39*	17.17 (16.50-18.16)	16.88±0.96	16.76 (16.07-17.79)	0.552*	0.552
Glutamine #	4.47±1.11*	4.40 (4.12-4.61)	4.53±0.21	4.50 (4.39-4.63)	0.400*	0.440
Glutathione‡	558±127	496 (469-668)	508±199*	384 (368-711)	0.141*	0.172
**HIPPOCAMPUS** analytes in nmol/g						
Glycine	928±65	913 (877-1002)	1022±57	1016 (975-1048)	**<0.001**	**<0.0012**
L-serine	719±44	730 (696-749)	606±35	601 (583-640)	**<0.001**	**<0.0012**
D-serine	274±23*	274 (267-288)	220±12	220 (217-229)	**<0.001***	**<0.0012**
Methionine	83.4±10.5	84.3 (77.2-91.1)	67.6±7.8	67.5 (62.1-72.9)	**<0.001**	**<0.0012**
Homocysteine	3.11±0.56	3.09 (2.61-3.63)	3.46±0.20	3.44 (3.32-3.59)	**0.033**	**0.0363**
Cysteine	111±28*	95.1 (89.5-146.1)	77.8±5.8	77.2 (73.7-81.4)	**<0.001***	**<0.0012**
Cysteinylglycine	4.38±1.28	5.13 (2.96-5.57)	2.35±0.21	2.40 (2.19-2.53)	**<0.001**	**<0.0012**
Threonine	334±76	315 (279-364)	207±41	203 (170-244)	**<0.001**	**<0.0012**
Glutamate #	10.13±0.27	10.05 (9.96-10.36)	10.34±0.54*	10.36 (10.19-10.81)	**0.043***	**0.0430**
Glutamine #	4.62±0.48*	4.54 (4.36-4.68)	5.31±0.33	5.37 (5.04-5.56)	**<0.001***	**<0.0012**
Glutathione	493±89	529 (406-576)	616±59	612 (565-655)	**<0.001**	**<0.0012**
**CEREBELLUM** analytes in nmol/g						
Glycine	917±86	904 (871-965)	1126±82	1133 (1082-1189)	**<0.001**	**<0.002**
L-serine	548±79	515 (489-625)	469±43	477 (441-498)	**0.002**	**0.0028**
Methionine	85.2±5.6	84.5 (80.2-89.4)	74.7±8.8	74.2 (70.1-79.5)	**<0.001**	**<0.002**

Homocysteine	2.53±0.26	2.55 (2.46-2.71)	2.43±0.23	2.46 (2.25-2.60)	0.317	0.352
Cysteine	83.8±22.8	90.2 (81.9-94.8)	110.3±18.5	113.2 (90.4-121.9)	**0.002**	**0.0028**
Cys-Gly	6.99±0.35*	6.98 (6.72-7.10)	2.48±0.33	2.45 (2.16-2.69)	**<0.001***	**<0.002**
Threonine	381±60	396 (350-421)	334±51	325 (291-381)	**0.029**	**0.0362**
Glutamate #	9.97±0.48	10.01 (9.60-10.36)	9.93±0.67	10.12 (9.48-10.37)	0.860	0.860
Glutamine #	6.51±0.71*	6.51 (6.08-6.74)	7.11±0.53	7.01 (6.90-7.37)	**0.001***	**0.002**
Glutathione	697±29*	692 (674-726)	628±58	632 (577-685)	**<0.001***	**<0.002**
**MUT Mice**	N=14		N=9			
**PLASMA**	**AVG±SD**	**Median (IQR)**	**AVG±SD**	**Median (IQR)**	**p-value t-test or MWU**	**p-value Benjamini-Hochberg**
Glycine	695±175	722 (533-805)	910±218	1023 (711-1101)	**0.016**	0.160
L-serine	129±37	123 (94-171)	115±20	122 (102-129)	0.265	0.442
Methionine	32.7±7.7	30.7 (26.0-41.5)	33.6±12.4	30.0 (34.2-49.7)	0.837	0.918
Homocysteine†	4.76±1.75	4.39 (3.63-6.12)	6.12±1.98	6.04 (4.53-7.93)	0.099	0.360
Cysteine†	133±42	123 (106-165)	157±29	150 (134-182)	0.153	0.360
Cysteinylglycine†	3.13±0.91	3.47 (2.26-3.83)	2.50±0.46*	2.74 (2.13-2.90)	0.124*	0.360
Threonine	101±30*	88 (79-119)	103±19	101 (88-118)	0.600*	0.857
Glutamate	45.2±10.2	45.3 (35.7-50.5)	44.8±11.6	47.3 (39.1-52.5)	0.918	0.918
Glutamine	578±83	557 (502-653)	618±54	617 (585-646)	0.180	0.360
Glutathione†	68±14	69.8 (59.9-77.7)	70.0±15.5	70.1 (57.6-79.7)	0.740	0.918
**CORTEX** analytes in nmol/g						
Glycine	1581±175	1583 (1458-1733)	1901±386*	1777 (1673-2008)	**0.011***	**0.030**
L-serine	565±64	575 (546-598)	587±40*	578 (561-592)	0.516*	0.6127
D-serine	238±32*	244 (226-258)	235±12	236 (230-244)	0.224*	0.352
Methionine	53.5±15.5	53.2 (41.5-64.0)	47.0±31.1*	37.7 (29.5-55.0)	0.124*	0.227
Homocysteine‡	3.54±0.44	3.65 (3.20-3.80)	2.24±0.23	2.30 (2.05-2.40)	**<0.001**	**0.00367**
Cysteine‡	123±13	123 (117-136)	54.7±9.7	55.4 (48.9-60.8)	**<0.001**	**0.00367**
Cysteinylglycine‡	3.69±0.80	3.45 (2.98-4.30)	2.26±0.39	2.20 (1.95-2.60)	**<0.001**	**0.00367**
Glutamate #	16.37±1.71	16.67 (15.55-17.66)	17.03±2.28	17.17 (15.75-18.94)	0.435	0.598
Glutamine #	4.53±1.43*	4.04 (3.81-4.87)	5.04±0.91	5.06 (4.25-5.77)	**0.039***	0.0858
Glutathione‡	544±67*	519 (484-586)	560±188	628 (369-741)	0.829*	0.829
**HIPPOCAMPUS** analytes in nmol/g						
Glycine	2063±162	2057 (1899-2180)	2134±254	2115 (2004-2307)	0.416	0.416
L-serine	669±40	668 (633-699)	607±60	616 (560-658)	**0.007**	**0.0154**
D-serine	232±20	227 (211-248)	204±23*	215 (183-221)	**0.039***	0.0612
Methionine	81.3±20.3	80.5 (62.6-101.3)	60.7±10.9	61.0 (50.5-72.3)	**0.011**	**0.020**
Homocysteine	3.08±0.67	3.23 (2.33-3.71)	3.55±0.40	3.64 (3.31-3.85)	**0.050**	0.0687
Cysteine	98±27	92.8 (78.9-119.4)	82.8±7.2	81.3 (76.3-88.0)	0.062	0.0758
Cysteinylglycine	4.52±1.33	4.01 (3.30-5.96)	2.43±0.29	2.42 (2.25-2.55)	**<0.001**	**<0.0055**
Threonine	338±47	350 (297-371)	218±46	218 (178-252)	**<0.001**	**<0.0055**
Glutamate #	10.13±0.79	10.29 (9.41-10.81)	10.22±1.45*	10.83 (9.77-11.19)	0.369*	0.4059
Glutamine #	4.86±1.57*	4.32 (3.99-5.07)	5.63±0.54	5.54 (5.27-5.92)	**0.005***	**0.0137**

Glutathione	498±90	492 (403-591)	616±69*	643 (601-656)	**0.003**	0.011
**Cerebellum** analytes in nmol/g						
Glycine	3217±301	3212 (3040-3370)	3329±519	3409 (2849-3797)	0.570	0.640
L-serine	621±71	632 (555-672)	569±80	563 (491-634)	0.119	0.170
Methionine	79.5±15.5	75.6 (70.1-86.6)	65.7±11.1	66.5 (54.0-75.1)	**0.031**	0.0775
Homocysteine	2.46±0.18*	2.48 (2.36-2.58)	3.04±0.34*	2.85 (2.83-3.35)	**<0.001***	**<0.0033**
Cysteine	85.7±15.3	81.5 (70.9-99.3)	117.9±22.4	120.0 (100.8-132.2)	**<0.001**	**<0.0033**
Cysteinylglycine	7.26±0.49	7.17 (6.82-7.67)	2.65±0.34	2.59 (2.49-2.88)	**<0.001**	**<0.0033**
Threonine	519±111	526 (444-587)	495±85	477 (427-555)	0.576	0.640
Glutamate #	10.10±0.42*	10.26 (9.78-10.45)	9.71±0.66	9.67 (9.25-10.05)	0.096*	0.160
Glutamine #	6.97±1.62*	6.50 (6.08-7.16)	7.45±0.69	7.45 (7.01-8.06)	**0.039***	0.078
Glutathione	636±40	642 (617-688)	638±49	651 (612-659)	0.898	0.898

Legend: Values of metabolites are compared between young 5-week-old mice of the J129X1/SvJ strain versus the C57Bl6/J strain for wild type (WT) mice and for homozygous mutant (MUT) mice. The distribution in each category is provided with mean (AVG) and standard deviation (SD), and as the median and interquartile range (IQR). Comparison is done either by the Student t-test or by the Mann-Whitney-U test (MWU) if not normally distributed as indicated by an asterisk *. A Benjamini-Hochberg corrected p-value is provided for the multiple comparisons within each tissue type. Significant p-values are highlighted in bold.

Supplemental Table 9. Impact of glycine 5% treatment on metabolites in young 5-week-old wild type J129 mice

Metabolite	No treatment	N=14	Treatment	N=14			
J129 5 wk old	Average±SD	Median IQR	Average±SD	Median IQR	p-value t-test / MWU	Benjamini p-value	% change
Glycine 5%							
PLASMA	N=16/14†			N=14			
Glycine	334±34*	326 (307-375)	1395±790*	1094 (1000-1280)	**<0.001***	**<0.0025**	**+318%**
L-serine	139±30	133 (116-172)	192±42	189 (162-214)	**<0.001**	**<0.0025**	**+38%**
Methionine	41.1±9.4*	39.4 (35.2-43.4)	39.6±7.5	40.7 (33.6-45.6)	0.886*	0.886	-4%
Homocysteine	4.9±1.1	5.0 (4.2-6.0)	7.4±1.8	7.7 (5.3-8.5)	**< 0.001**	**<0.0025**	**+51%**
Cysteine	128±21	128 (117-135)	146±28	136)(127-170)	0.059	0.118	+14%
Cysteinylglycine	4.1±0.3	4.1 (3.9-4.3)	2.7±0.4	2.6 (2.4-2.9)	**<0.001**	**<0.0025**	**-34%**
Threonine	115±24	106 (94-129)	114±20	113 (99-128)	0.882	0.886	-1%
Glutamate	48.5±12.3	44.0 (40.2-54.1)	51.6±19.2	46.1 (37.7-63.2)	0.599	0.749	+6%
Glutamine	613±78	616 (538-687)	685±131	683 (574-786)	0.073	0.122	+12%
Glutathione	74±11*	79.7 (63.3-83.9)	70±6	68.2 (65.4-73.9)	0.164*	0.234	-5%
CORTEX	N=16/13‡			N=14			
Glycine	855±58	829 (792-893)	1356±171	1338 (1219-1429)	**<0.001**	**<0.0018**	**+59%**
L-serine	657±52	659 (601-691)	1004±127*	986 (938-1028)	**<0.001***	**<0.0018**	**+53%**
D-serine	302±25*	293 (188-313)	469±51	293 (288-313)	**<0.001***	**<0.0018**	**+55%**
Methionine	70.8±14.4*	63.5 (59.6–68.3)	54.0±12.4	51.6 (43.4-61.6)	**0.001***	**0.0018**	**-24%**
Homocysteine	3.80±0.62*	3.80 (3.60-4.25)	2.81±0.71	2.97 (2.08-3.47)	**<0.001***	**<0.0018**	**-26%**
Cysteine	111±35	107 (87-125)	87±29*	84.8 (60-114)	0.068*	0.0831	-22%
Cysteinylglycine	2.82±0.70	3.00 (2.15-3.25)	3.84±0.41	3.85 (3.51-4.20)	**<0.001**	**<0.0018**	**+36%**
Glutamate #	16.8±2.4	1.73 (1.68-1.84)	17.0±1.4	1.73 (1.56-1.78)	0.813	0.813	+1%
Glutamine #	4.47±1.11*	4.46 (4.21-4.60)	5.16±0.76	5.04 (4.49-5.60)	**0.005***	**0.0079**	**+15%**
Glutathione	558±127	496 (456-676)	688±172*	665 (519-849)	**0.025***	**0.034**	**+23%**
HIPPOCAMPUS	N=16			N=13			
Glycine	928±65	913 (877-1002)	1110±119	1101 (1081 (1178)	**<0.001**	**<0.0022**	**+20%**
L-serine	719±44	730 (696-749)	861±84*	881 (835-914)	**<0.001***	**<0.0022**	**+20%**
D-serine	274±23*	274 (267-288)	298±28	310 (277-314)	**0.007***	**0.0126**	**+9%**
Methionine	83.4±10.5	84.3 (77.2-91.1)	43.0±5.5	42.4 (38.8-47.7)	**<0.001**	**<0.0022**	**-48%**
Homocysteine	3.11±0.56	3.09 (2.61-3.63)	3.43±0.33	3.50 (3.23-3.66)	0.081	0.111	+10%
Cysteine	111±28*	95.1 (89.5-146.1)	51±3*	50.1 (47.9-51.7)	**<0.001***	**<0.0022**	**-54%**
Cysteinylglycine	4.38±1.28*	5.13 (2.96-5.57)	4.47±0.97*	4.62 (4.49-4.91)	0.475*	0.553	+2%
Glutamate #	10.1±0.3	10.05 (9.96-10.36)	11.2±1.2	11.04 (10.63-12.07)	**0.008**	**0.0126**	**+11%**
Glutamine #	4.61±0.48	4.54 (4.36-4.68)	4.46±0.78*	4.43 (3.97-4.69)	0.268*	0.553	-3%
Glutathione	493±89*	529 (406-576)	507±25	514 (482-526)	0.779*	0.553	+3%
CEREBELLUM	N=16			N=14			
Glycine	917±86	904 (871-965)	1125±108	1124 (1048-1172)	**<0.001**	**<0.002**	**+23%**
L-serine	548±79	515 (489-625)	1156±172	1151 (991-1300)	**<0.001**	**<0.002**	**+111%**
Methionine	85.3±5.6	84.5 (80.2-89.3)	57.4±12.2	58.0 (47.3-67.3)	**<0.001**	**<0.002**	**-33%**
Homocysteine	2.53±0.26*	2.55 (2.46-2.71)	2.98±0.20	3.03 (2.79-3.12)	**<0.001***	**<0.002**	**+18%**

Cysteine	83.8±22.8*	90.2 (81.9-94.8)	72.5±15.3	71.1 (58.2-82.6)	**0.012***	**0.015**	-13%
Cysteinylglycine	6.99±0.35	6.98 (6.73-7.10)	7.40±0.32*	7.47 (7.25-7.65)	**0.003***	**0.005**	+6%
Threonine	381±60	396 (350-421)	319±47	305 (282-368)	**0.004**	**0.006**	-16%
Glutamate #	9.97±0.48	10.01 (9.60-10.36)	9.54±0.71	9.54 (9.21-10.25)	0.061	0.068	-4%
Glutamine #	6.51±0.71*	6.51 (6.08-6.74)	5.20±0.55	5.04 (4.80-5.67)	**< 0.001***	**<0.002**	-20%
Glutathione	697±29	692 (674-726)	691±35	688 (663-723)	0.621	0.621	-1%

Legend: Values of metabolites in J129 mice at age 5 weeks are shown comparing wild type mice without treatment to wild type mice challenged with glycine 5% in drinking water administered for one week. The distribution in the untreated and the treated mice is shown as mean (AVG) and standard deviation (SD). Values of metabolites in plasma are in µM, and values of the metabolites in forebrain cortex, hippocampus and cerebellum are shown in nmol/g tissue (# except for glutamate and glutamine shown in µmol/g tissue). For stereoselective amino acids, there were 16 untreated samples and 14 treated samples except for the hippocampus where there were only 13 samples. For the sulfur metabolites, there were always 14 treated mouse samples except for the hippocampus where there were 13 samples. For the untreated mice, there were 14 plasma samples†, 13 cortex samples‡, and 16 hippocampus and cerebellum samples. A significant deviation of the normal distribution is indicated by an asterisk *. Comparisons are done by Student t-test or by Mann-Whitney-U test if any of the two populations deviates from the normal distribution as indicated by an asterisk *, and p-values are shown. A Benjamini-Hochberg corrected p-value is provided for the multiple comparisons within each tissue type. The percentage differences between the untreated and the treated mice are given with green values for significant increase and red values for a significant decrease.

Supplementary Figures

Supplemental Figure 1. Relation between glycine levels in plasma and liver

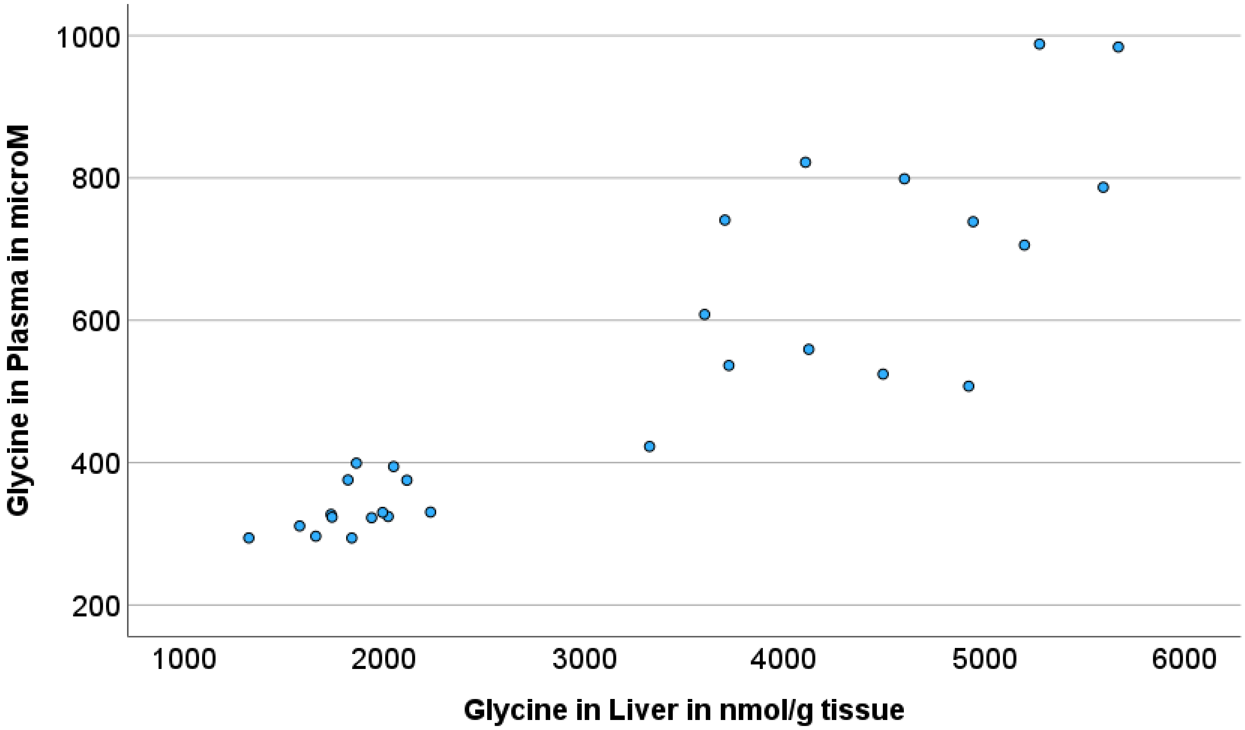

Legend: The graph shows the levels of glycine in liver in relation to the glycine levels in plasma for the WT mice grouped on the left-hand side and for the MUT mice grouped on the upper right hand of the graph. Within the group of the MUT mice, there is a significant moderate positive correlation (r^2=0.632 p=0.015).

Supplemental Figure 2. Cortex guanidinoacetate as a relation of glycine levels in cortex and plasma

A.

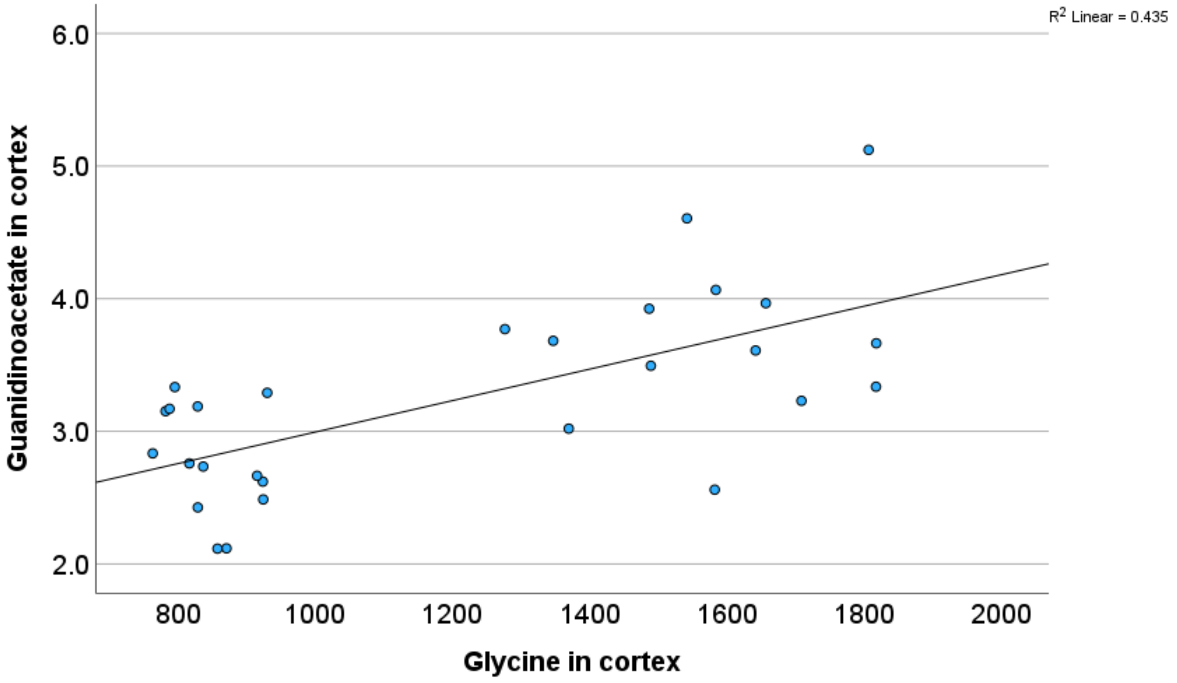

B.

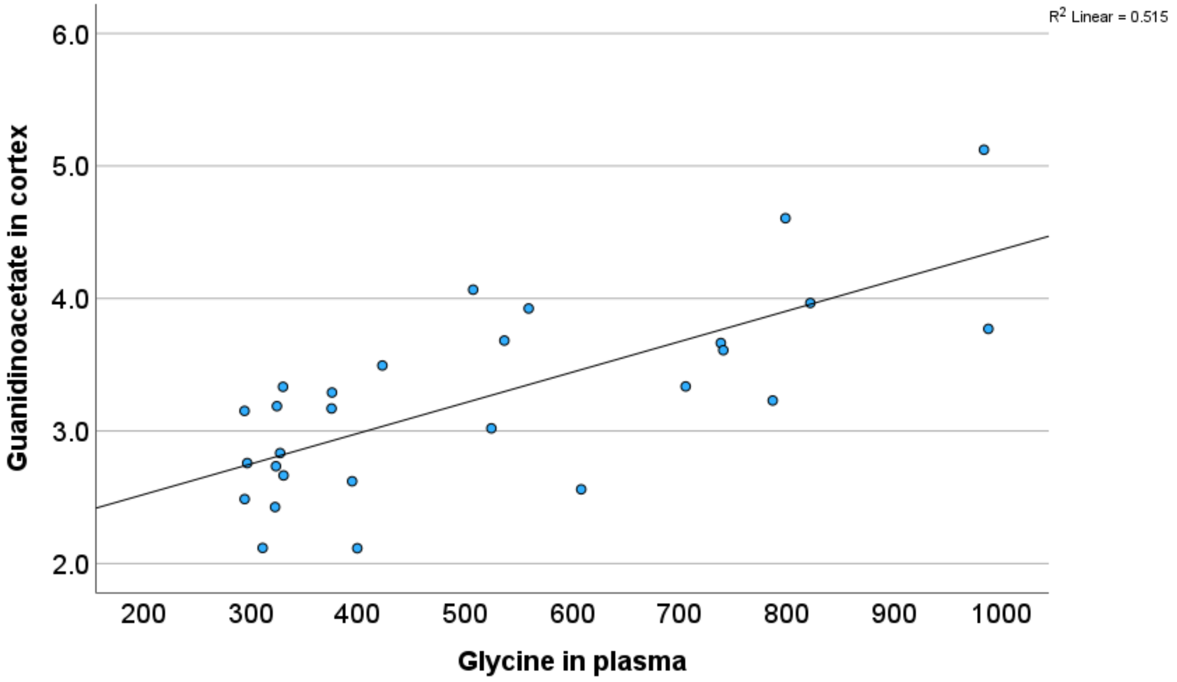

Legend: The guanidinoacetate level in cortex in nmol/g tissue is shown in relation to (A) glycine in cortex in nmol/g tissue and (B) to glycine in plasma in µM.

Supplemental Figure 3. L-threonine dehydrogenase in cortex of B6 mice

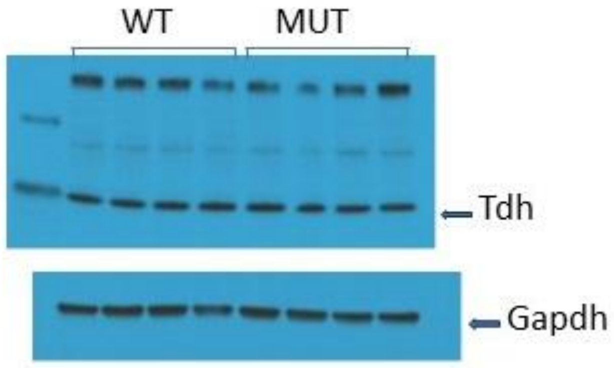

Legend: The intensity of the signal for L-threonine dehydrogenase protein (Tdh) is unchanged between wild type B6 mice and mutants for nonketotic hyperglycinemia in the cortex. Gapdh was used as a loading control.

Supplemental Figure 4. Levels of TBARS in the cortex of mice with NKH

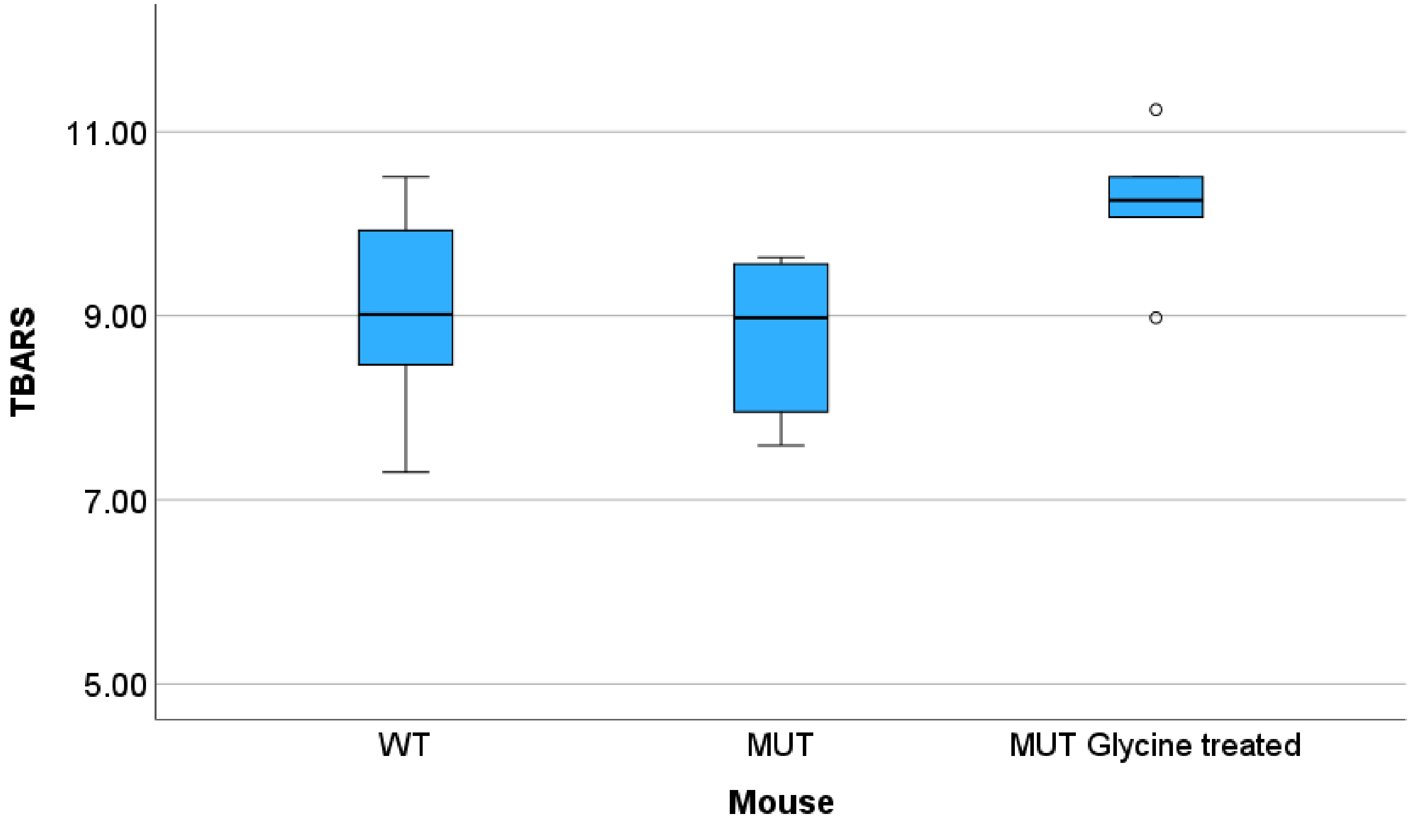

Legend: Levels of TBARS in the cortex of wild type mice (WT), mutant mice with NKH (MUT) and mutant mice treated with glycine (MUT Glycine treated). This shows a statistically significant difference between the MUT mice and the MUT mice treated with glycine, but not between MUT and WT mice.

Supplemental Figure 5: Analysis of the lipidomics data for MUT and WT mice

A. Principal Component analysis

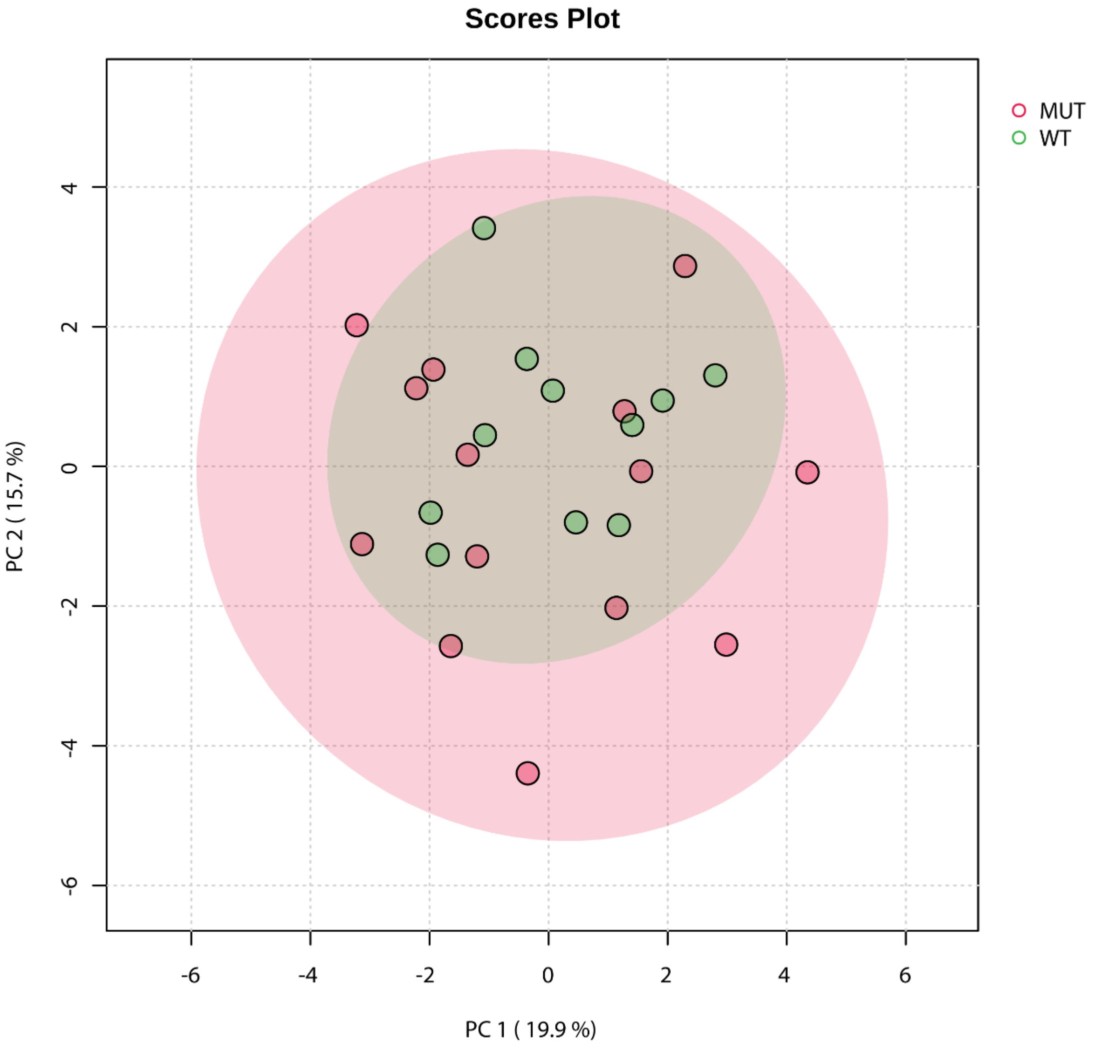

B. Lipid ontology analysis

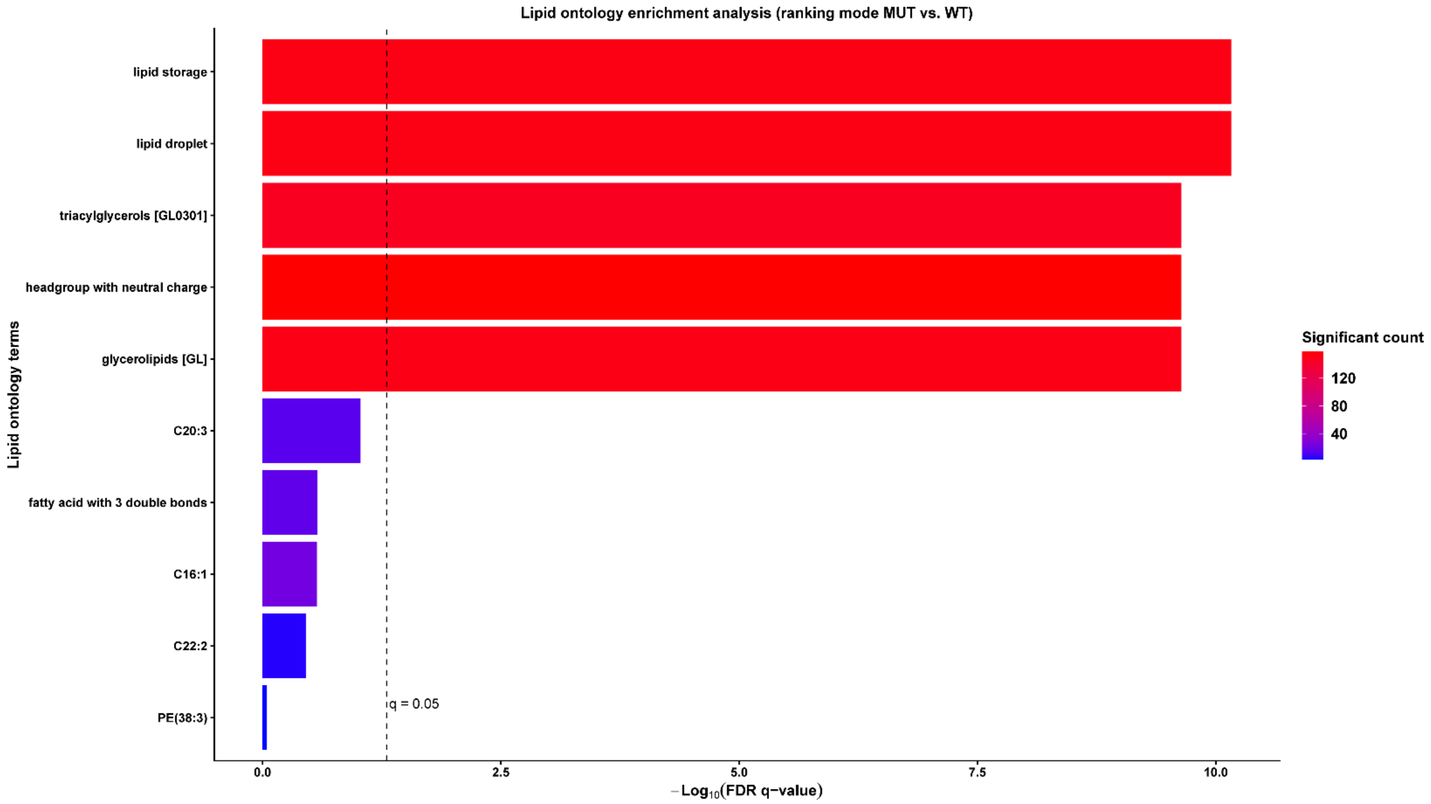

Legend: Lipidomics data of 5-week-old J129 mice were analyzed. A. Principal component analysis of the data did not separate the MUT mice from WT mice. B. Lipid ontology shows increased representation of lipid classes for lipid storage in lipid droplets such as triacylglycerols, glycerolipids, and lipids with a neutral charge. It also showed decreased representation of certain lipids with low unsaturation such as C18:1, 20:3, and C22:2.

Supplemental Figure 6: Network analysis of the lipidomics data in the brain cortex

A. Lipid networks comparing MUT versus WT mice

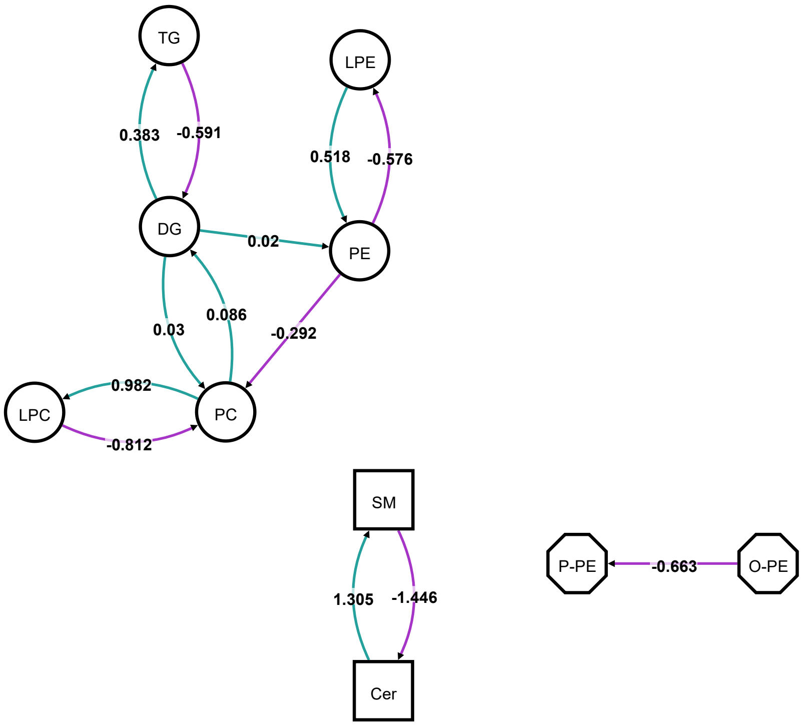

B. Fatty acid networks comparing MUT versus WT mice

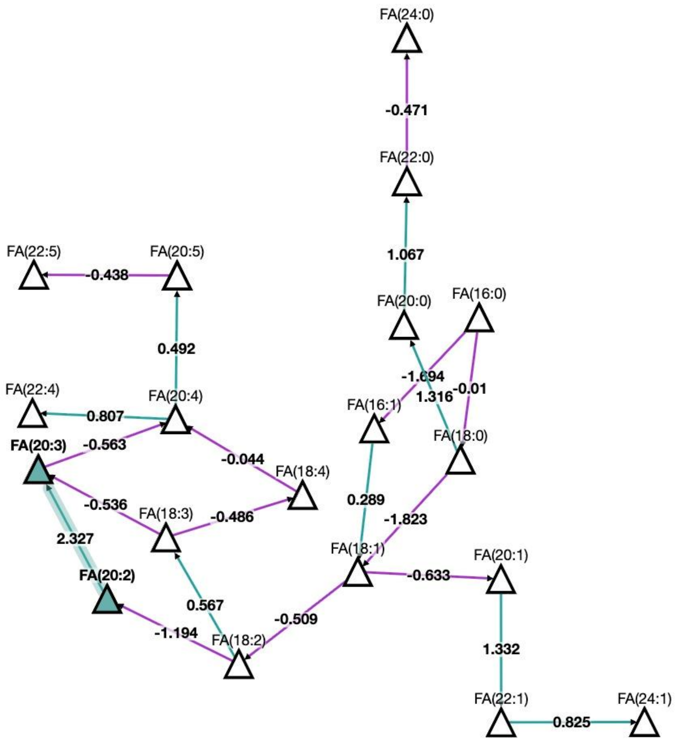

Legend: Lipidomics data were obtained from brain cortex of 5-week-old J129 mice comparing MUT versus WT mice. Network analysis was done by BioPAN and the results shown for (A) the lipid networks, and (B) the fatty acid networks. Green data nodes correspond to active lipids and green shaded arrows to active pathways. Reactions with a positive Z-score have green arrows while negative Z-scores are purple colored. The data were generated with a p-value of 0.05 and no paired-data. (A) In the lipid network we notice a shift from lyso-phosphatidylcholines (or ethanolamines) to phosphatidylcholines (ethanolamines, respectively), and a shift from ceramides towards sphingomyelines. (B) In the fatty acid network, we see a shift towards elongated fatty acids of chain lengths 20 to 22, and towards poly-unsaturated species with 2 to 4 unsaturated bonds.

Supplemental Figure 7A:

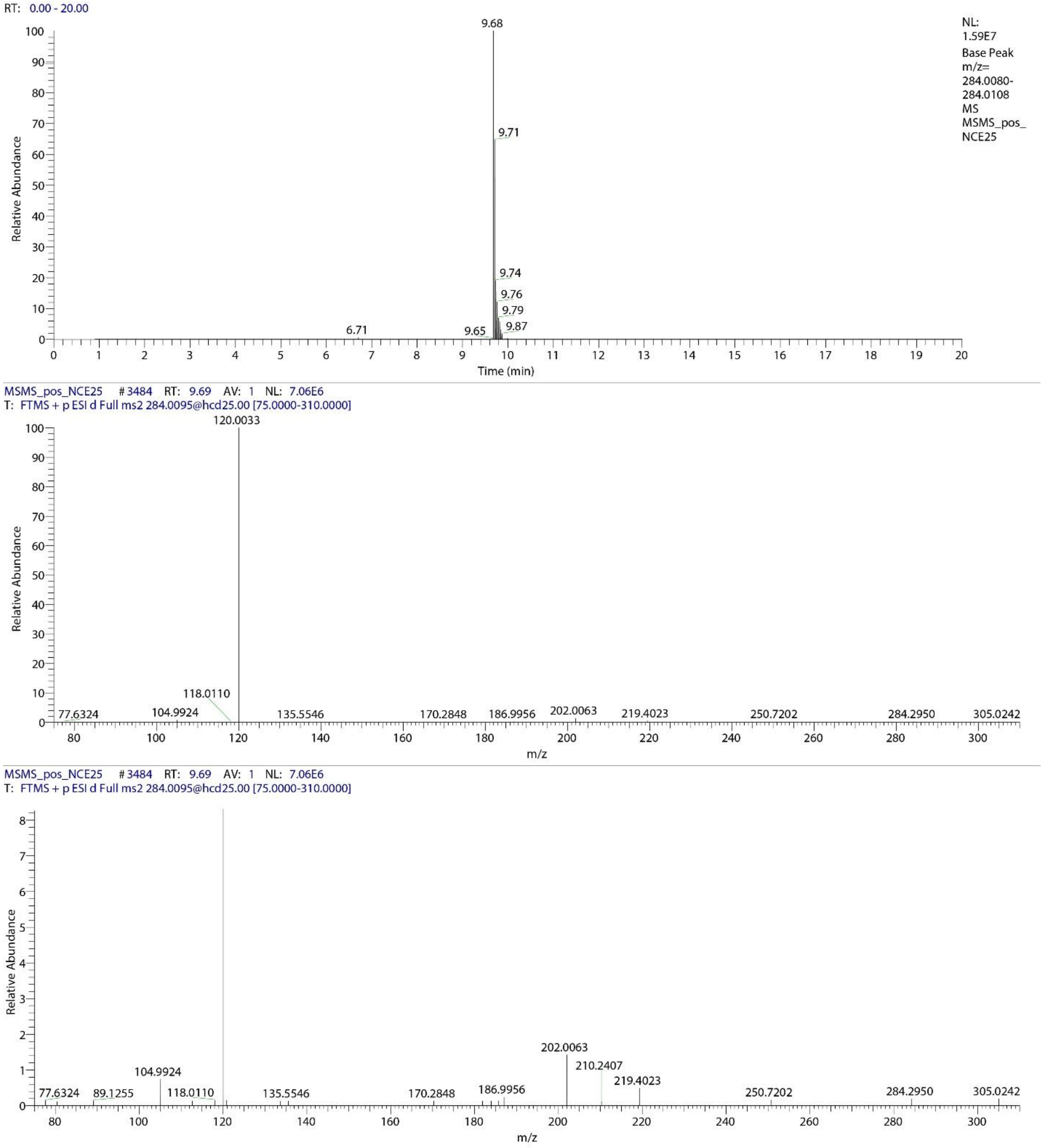

Legend: 1. Of compound C in the metabolomics diagram, the base peak ion chromatogram of an unknown compound of interest (m/z = 284.0095) obtained by positive ion High Performance Liquid Chromatography – Electrospray Ionization – Mass Spectrometry (HPLC-ESI-MS) in Hydrophilic Interaction Liquid Chromatography (HILIC) from a pooled QC sample. Sample was analyzed in MS/MS. **2.** Tandem mass spectrum (MS/MS) of an unknown compound with Normalized Collision Energy (NCE) of 25 at a retention time of 9.69 minutes. **3.** Zoomed in MS/MS spectrum of 2. Relative Abundance in y-axis is zoomed in to observe low abundant fragments.

Supplemental Figure 7B

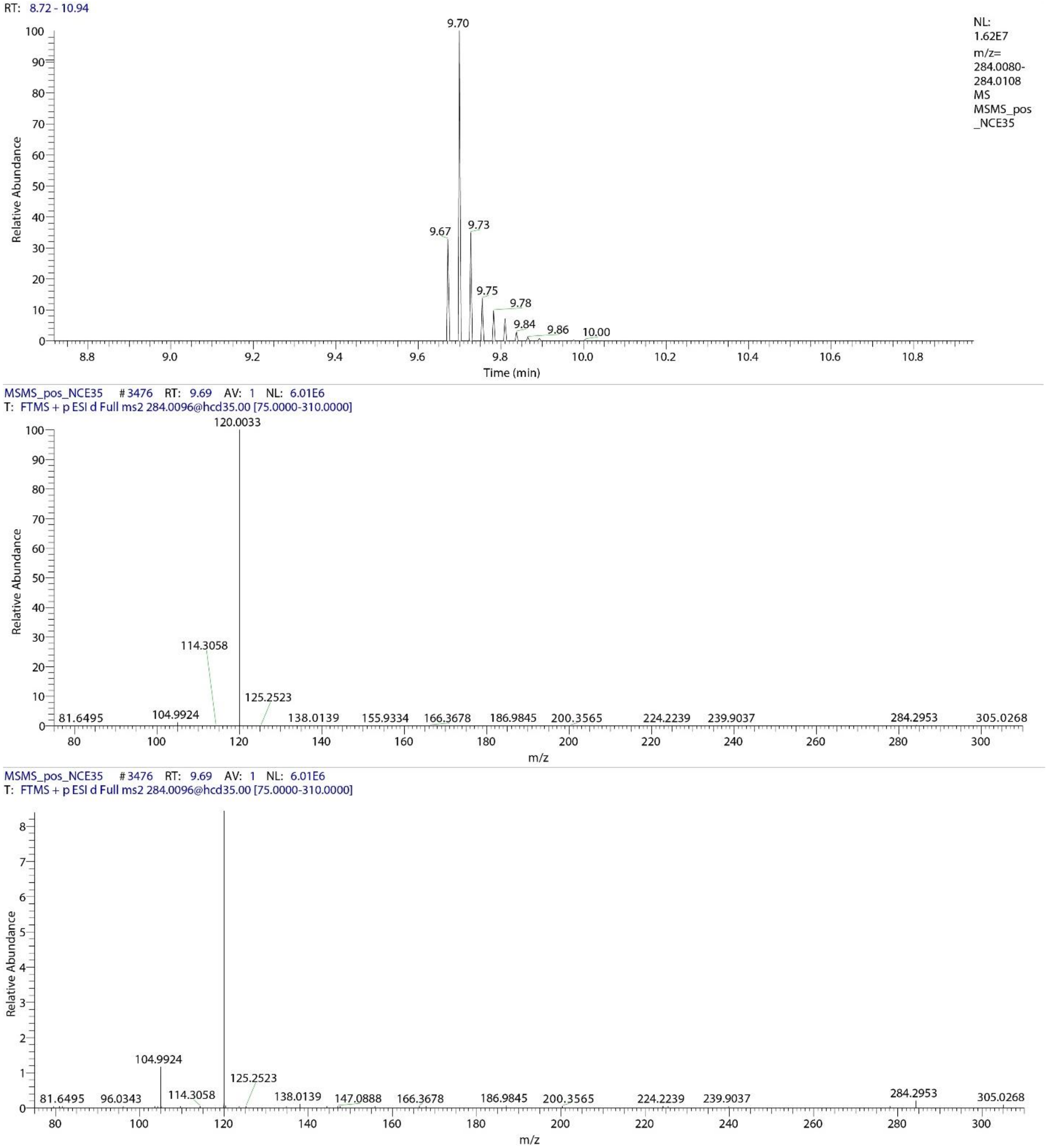

Legend: 1. Of compound C in the metabolomics diagram, the base peak ion chromatogram of an unknown compound of interest (m/z = 284.0096) obtained by positive ion High Performance Liquid Chromatography – Electrospray Ionization – Mass Spectrometry (HPLC-ESI-MS) in Hydrophilic Interaction Liquid Chromatography (HILIC) from a pooled QC sample. Sample was analyzed in MS/MS. **2.** Tandem mass spectrum (MS/MS) of an unknown compound with Normalized Collision Energy (NCE) of 35 at a retention time of 9.69 minutes. **3.** Zoomed in MS/MS spectrum of 2. Relative Abundance in y-axis is zoomed in to observe

low abundant fragments.

Supplemental Figure 7C

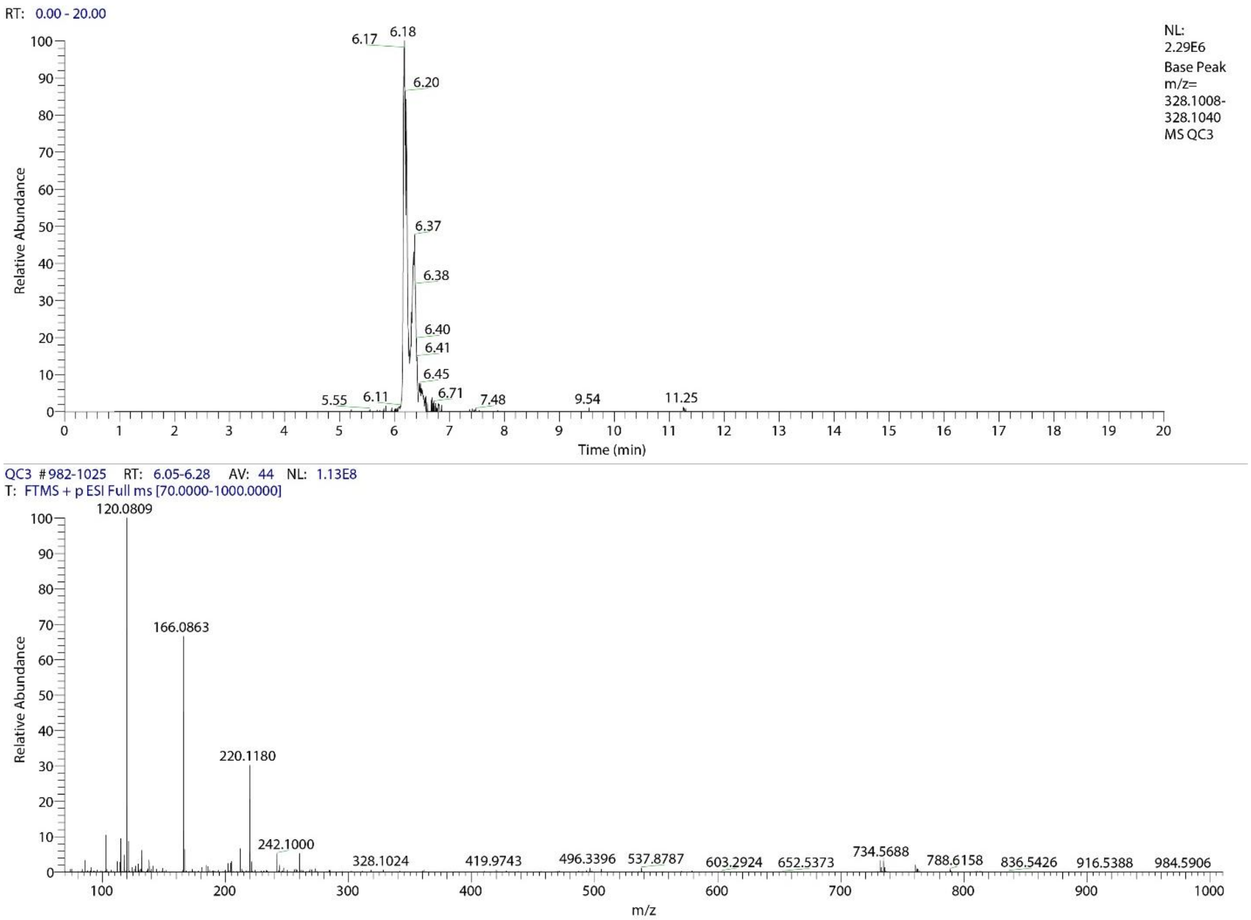

Legend: 1. Of compound B in the metabolomics diagram, the base peak ion chromatogram of an unknown compound of interest (m/z = 328.1024) obtained by positive ion High Performance Liquid Chromatography – Electrospray Ionization – Mass Spectrometry (HPLC-ESI-MS) in Hydrophilic Interaction Liquid Chromatography (HILIC) from a pooled QC sample. Sample was analyzed in full scan MS. **2.** Mass spectrum (MS) between 6.05 – 6.28 minutes with the unknown compound of interest.

Supplemental Figure 7D

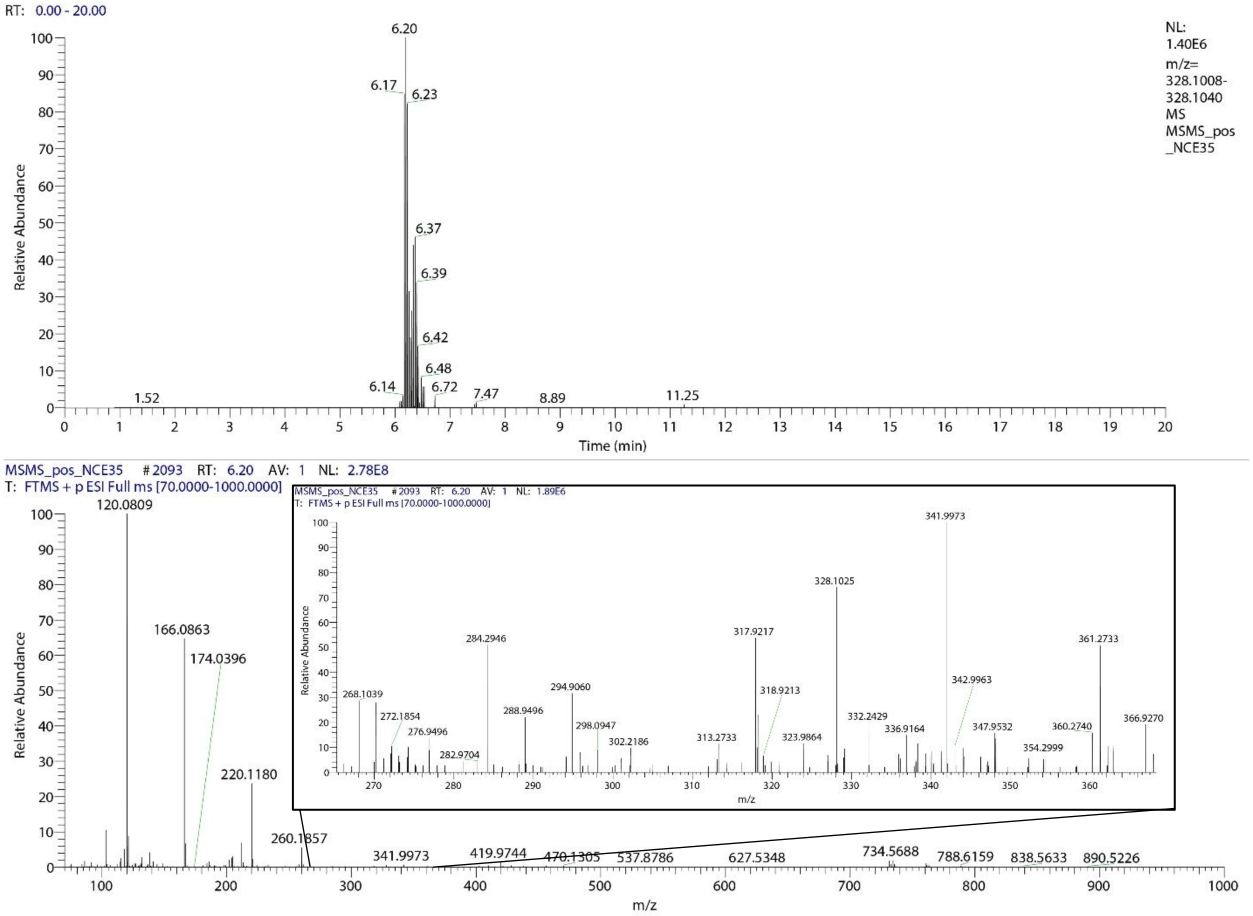

Legend: Of compound B in the metabolomics diagram, the base peak ion chromatogram of an unknown compound of interest (m/z = 328.1024) at 6.20 minutes obtained by positive ion High Performance Liquid Chromatography – Electrospray Ionization – Mass Spectrometry (HPLC-ESI-MS) in Hydrophilic Interaction Liquid Chromatography (HILIC) from a pooled QC sample. Sample was analyzed in MS/MS. **2.** Tandem mass spectrum (MS/MS) of an unknown compound with Normalized Collision Energy (NCE) of 35 at a retention time of 6.20 minutes. **3.** Zoomed in MS/MS spectrum of 2. m/z between 266-368 is zoomed in to observe low abundant fragments.

Supplemental Figure 7E

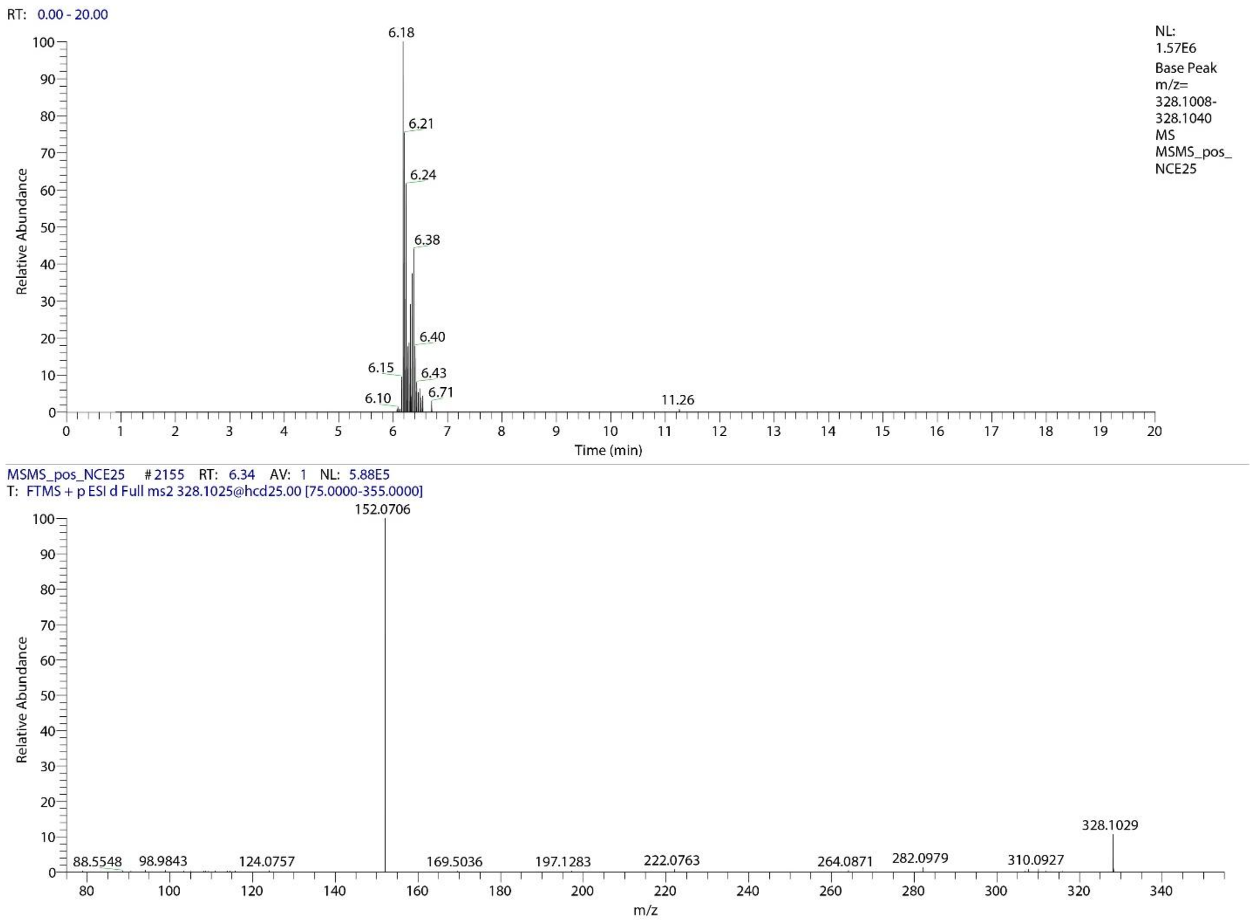

Legend: 1. Of compound A in the metabolomics diagram, the base peak ion chromatogram of an unknown compound of interest (m/z = 328.1025) obtained by positive ion High Performance Liquid Chromatography – Electrospray Ionization – Mass Spectrometry (HPLC-ESI-MS) in Hydrophilic Interaction Liquid Chromatography (HILIC) from a pooled QC sample. Sample was analyzed in MS/MS. **2.** Tandem mass spectrum (MS/MS) of an unknown compound with Normalized Collision Energy (NCE) of 25 at a retention time of 6.35 minutes.

Supplemental Figure 7F

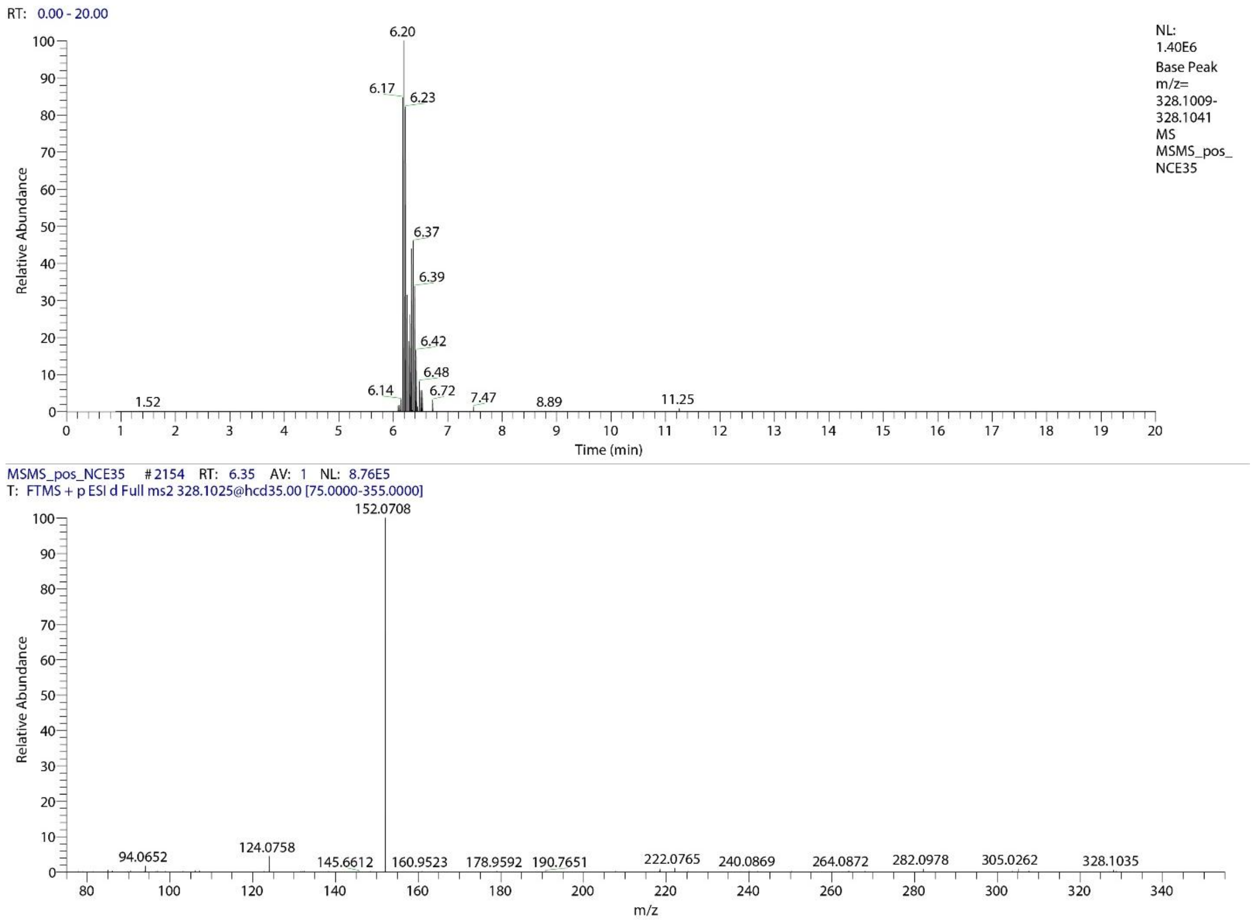

Legend: 1. Of compound A in the metabolomics diagram, the base peak ion chromatogram of an unknown compound of interest (m/z = 328.1025) obtained by positive ion High Performance Liquid Chromatography – Electrospray Ionization – Mass Spectrometry (HPLC-ESI-MS) in Hydrophilic Interaction Liquid Chromatography (HILIC) from a pooled QC sample. Sample was analyzed in MS/MS. **2.** Tandem mass spectrum (MS/MS) of an unknown compound with Normalized Collision Energy (NCE) of 35 at a retention time of 6.35 minutes.

Supplemental Figure 8. SLC6A20 in cortex of J129 mice

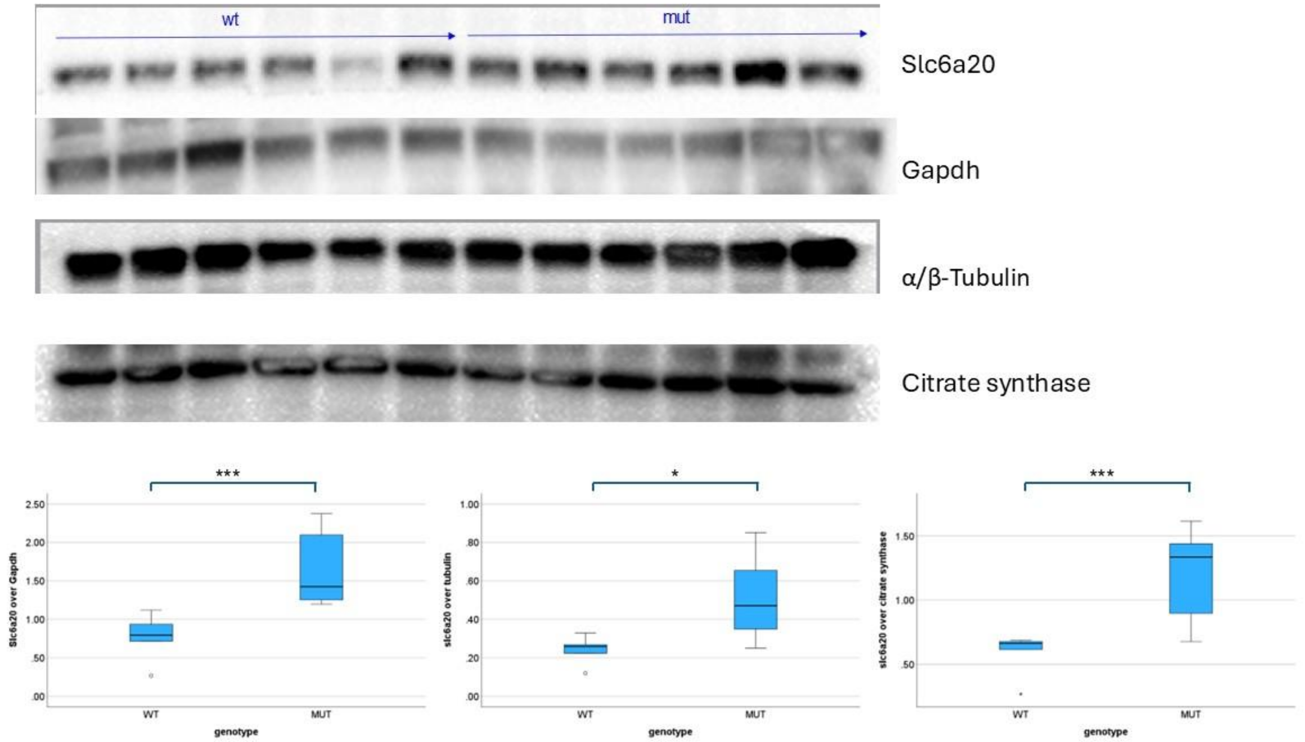

Legend: The abundance of the SLC6A20 carrier protein was increased in MUT mice compared to WT mice using GAPDH as control (WT 0.79 (0.60-0.98) median (IQR), MUT 1.42 (1.24-2.17), p=0.002), or using α/β-tubulin as control (WT 0.243±0.069 (AVG±SD), MUT 0.508±0.219, p=0.03), or using citrate synthase as control (WT 0.66 (0.53-0.68) (median (IQR)) vs. MUT 1.33 (0.84-1.48), p=0.009).

Supplemental Figure 9: DPY30 in nuclear cortex of 5-week old J129 mice

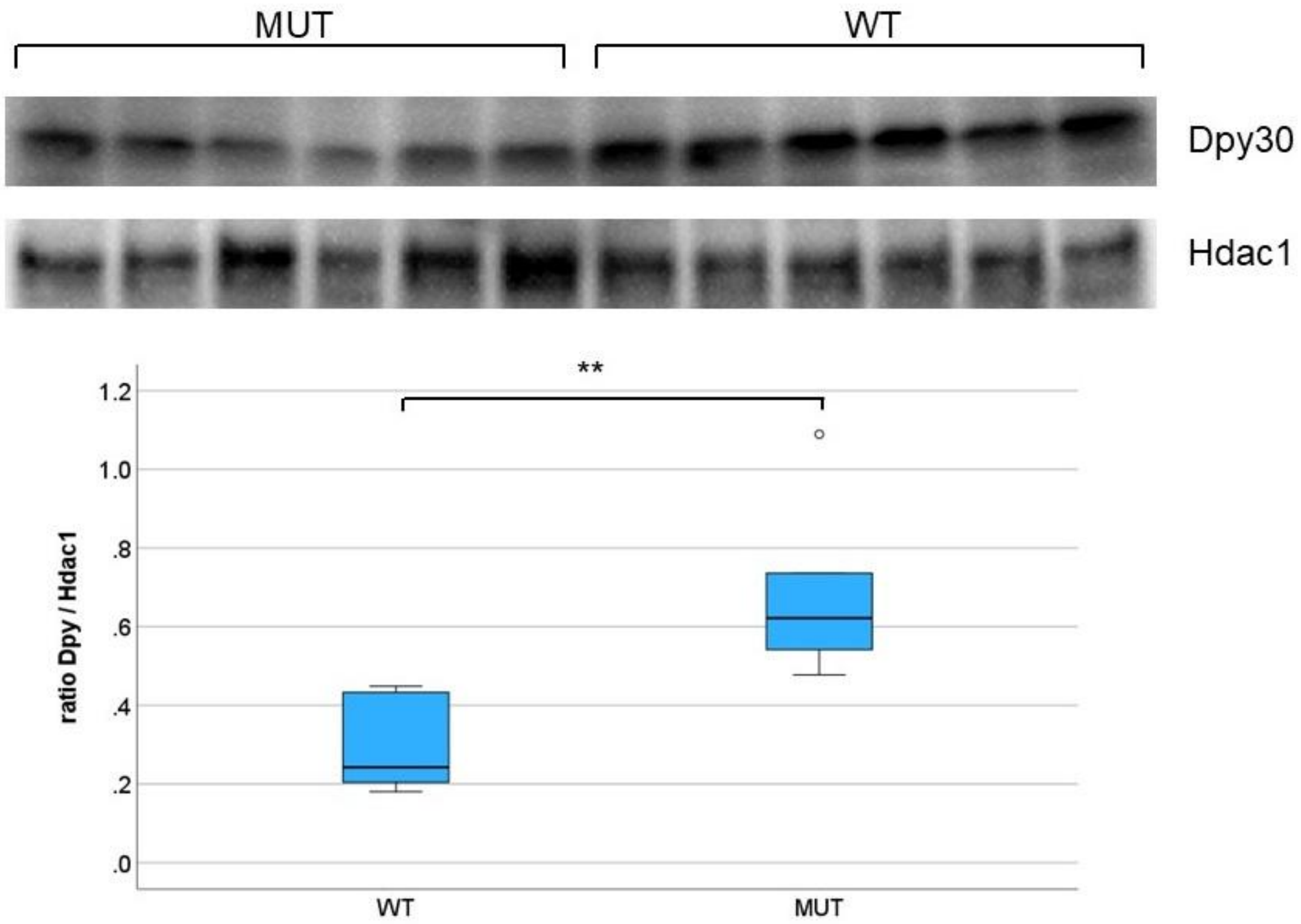

Legend: The amount of DPY30 protein is quantified by western blot using HDAC1 in nuclear extracts of cortex of 5-week-old J129 mice as loading control and comparing MUT mice with WT mice. * = p<0.05, ** = p<0.01, *** p<0.001.

Supplemental figure 10. Proteomics of mouse cortex with identification of mitochondrial proteins

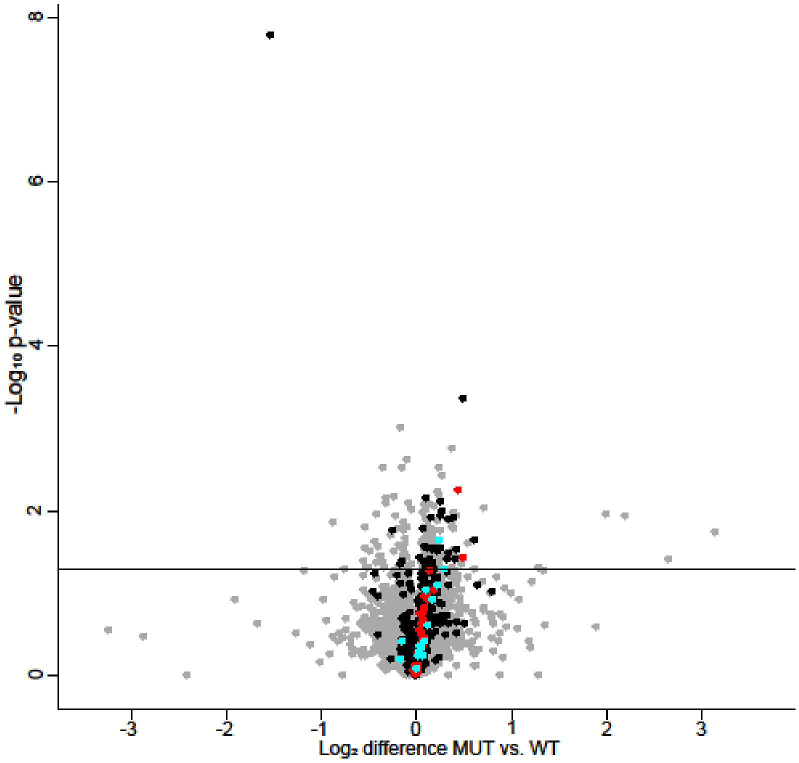

Legend: A volcano plot from whole-cell untargeted proteomics data of cortex in young 5-week-old J129 mice comparing mutant (MUT) versus wild-type (WT) mice. Proteins located in the mitochondrial are derived from the mitocarta 3.0 database and highlighted in black, with subunits of respiratory chain complex I highlighted in blue and proteins of respiratory chain complex IV in red. The tilt of these proteins to the right indicates the upregulation of the most mitochondrial proteins.

Supplemental Figure 11. Respiratory chain complex abundance

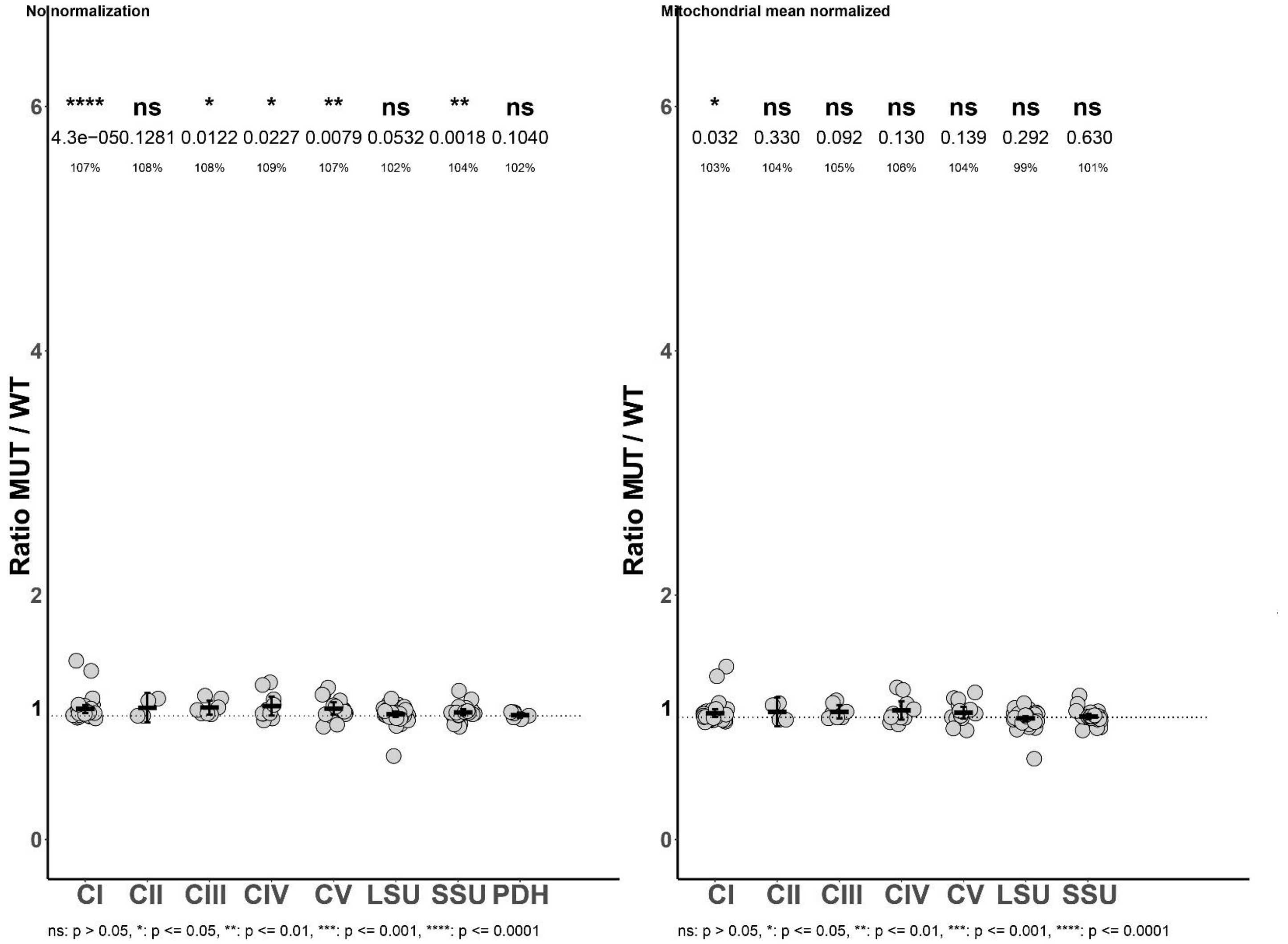

Legend: The relative complex abundances (RCA) for respiratory chain enzyme complexes, mitoribosome subunits, and pyruvate dehydrogenase complex are shown. On the left panel, the RCA is shown without mitochondrial abundance correction and on the right panel they are shown after correction for the mitochondrial abundance. Abbreviations. Complex I = CI; Complex II = CII; Complex III = CIII, complex IV = CIVI; Complex V = CV; large subunit of the mitoribosome = LSU; small subunit of the mitoribosome = SSU; pyruvate dehydrogenase = PDH. The legend of the statistically significant findings is shown in the figure. No significant change in the abundance of these mitochondrial complexes is shown, particularly after correction for the mitochondrial mass.

Supplemental figure 12. Nrf2 protein in the cytosol and nucleus in the cortex of the 5-week-old J129 mice

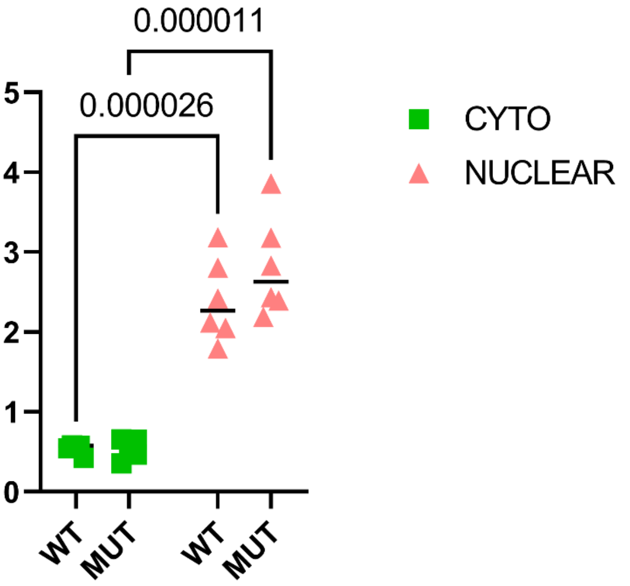

Legend: The amount of the NRF2 protein in the cytosol and the nucleus is shown, measured by ELISA in the cortex of 5-week-old J129 mice. There was a larger fraction of the NRF2 protein in the nucleus than in the cytosol, but there was no significant difference between the wild type (WT) and the mutant (MUT) mice.
